# Human leukocyte antigen variation is associated with cytomegalovirus serostatus in healthy individuals

**DOI:** 10.1016/j.ajhg.2025.02.007

**Published:** 2025-03-05

**Authors:** Juliano A. Boquett, Jürgen Sauter, Alexander H. Schmidt, Martin Maiers, Jill A. Hollenbach

**Affiliations:** 1 Department of Neurology, University of California, San Francisco, San Francisco, CA, USA; 2 DKMS Group, Tübingen, Germany; 3 CIBMTR (Center for International Blood and Marrow Transplant Research), NMDP, Minneapolis, MN, USA; 4 Department of Epidemiology and Biostatistics, University of California, San Francisco, San Francisco, CA, USA

**Keywords:** infection, CMV, HLA, serostatus, immunogenetics, binding pockets

## Abstract

Cytomegalovirus (CMV) is a common β-herpes virus worldwide with an estimated seroprevalence among the general population of 83%. Primary infection is usually benign; however, CMV can cause severe morbidity in newborns in whom it is acquired congenitally, as well as immunocompromised individuals. Understanding the role of immunogenetic variation in risk for CMV infection can provide insight into the immune control of this ubiquitous pathogen. Here, we evaluated the association of human leukocyte antigen (*HLA*) genetic variation with CMV seropositivity in more than 518,000 individuals from two independent cohorts. We found three *HLA* class II alleles (*HLA-DRB1^∗^04:03* with risk; *HLA-DRB1^∗^01:03* and *HLA-DRB1^∗^07:01* with protection) to be significantly associated with CMV serostatus across both cohorts and in multiple population subgroups. Interestingly, *HLA-DRB1^∗^04:03* and *HLA-DRB1^∗^01:03*, the alleles with the strongest observed effect, are relatively rare, while common homologous alleles show no association with CMV. We show that these differences are mediated by changes in charge and volume to two key pockets in the peptide-binding groove of the HLA molecule, providing a structural basis for the observed association. Our results provide population-scale evidence for the role of HLA in mediating infection with this ubiquitous human virus and a framework for understanding immunological conditions necessary for efficient viral control.

## Teaser

Cytomegalovirus (CMV) is a common virus worldwide; while infection is usually benign, it can cause severe illness in newborns and immunocompromised individuals. We evaluated variation in immune genes in risk for CMV in more than 518,000 individuals to better understand how the immune response to the virus differs between individuals.

## Introduction

Cytomegalovirus (CMV) is an ancient β-herpes virus (type 5) that is ubiquitous in all human populations. Infection with CMV is common across the globe, with an estimated seroprevalence among the general population of 83%.^1^ However, the estimated CMV seroprevalence varies among WHO (World Health Organization) regions: 66% in Europe, 75% in the Americas region, 86% in Southeast Asia, 88% in Africa and Western Pacific regions, and 90% in the Eastern Mediterranean.^1^ Active CMV infection can occur in three different ways: (1) primary infection (including congenital infection), (2) reactivation of latent CMV, and (3) reinfection with a new strain of CMV.^2^ Generally, CMV seroprevalence is higher among women, older age groups, those from lower socioeconomic status (SES), and those in developing countries.^3^ CMV infection is typically asymptomatic in healthy individuals, establishing a lifelong latent infection. However, CMV can cause long-term impacts on the health status of immunocompromised individuals, transplant recipients, those with late-stage human immunodeficiency virus (HIV) infection, and the elderly and congenitally infected neonates.^4^^,^^5^

CMV infection and its adverse sequelae are closely tied to social factors. Lower SES is associated with increased mortality from CMV among adults in the US, independent of age.^6^ Globally, CMV seroprevalence is correlated with ancestry, SES, education level,^7^ and increased mortality in older age.^6^ Furthermore, congenital CMV infection is associated with lower SES and young maternal age, is more prevalent among HIV-seropositive patients, and is significantly more common in low- and middle-income countries in comparison to high-income countries.^8^

CMV primary infection initiates with viral replication in the mucosal epithelium, disseminating to monocytic cells of myeloid lineage, where it establishes latent infection.^9^ After the establishment of primary infection, virus particles are processed and presented by antigen-presenting cells, which elicits the antigen-specific immune response. The virus efficiently adapts to the human immune system and can evade it using different pathways, e.g., by downregulating human leukocyte antigen (*HLA*) class I expression on the cell surface,^10^ causing a persistent asymptomatic infection.

*HLA* genes are notably the most polymorphic of the human genome.^11^ This remarkable variation leads to distinctive peptide-binding repertoires, whereby individuals with different *HLA* genotypes may exhibit different abilities in antigen presentation and differentially elicit immune responses, underlying *HLA* associations with human disease, including viral infections.^12^ For example, *HLA* class I variants have been associated with the slow progression of HIV infection,^13^ while *HLA* class II variants are associated with both viral persistence and clearance for hepatitis B (HBV) and C (HCV) viruses.^14^ Our work and that of others has also demonstrated a role for *HLA* in COVID-19 disease outcomes.^15^^,^^16^ Notably, *HLA* associations with infection by herpesvirus other than CMV have been described.^17^ While the role of *HLA* variation has been examined in the context of CMV, studies have largely focused on immunosuppressed patients in the transplant setting.^18^^,^^19^^,^^20^^,^^21^

In addition to their role in infection immunity, *HLA* genes are the primary determinant for tolerance or rejection in solid organ and hematopoietic stem cell transplant (HSCT). *HLA*-matched transplantation between a patient and donor presents the best outcome due to the prevention of immune rejection of foreign tissue and the facilitation of immune reconstitution.^22^ The NMDP (formerly known as the National Marrow Donor Program) is a large and diverse donor registry with over 9 million potential HSCT donors registered in the US. DKMS has registered over 12 million HSCT registry members in at least seven countries, including 1.1 million potential bone marrow donors registered in the US. Registries store demographic data such as gender, age, race/ancestry, zip code of residence, and high-resolution *HLA* genotyping for registered donors. Because CMV reactivation after allogeneic HSCT (allo-HSCT) has been associated with increased transplant-related mortality and end-organ diseases such as pneumonia, hepatitis, and colitis,^23^ and CMV infection is associated with increased risk for graft-versus-host disease (GVHD),^24^^,^^25^ CMV-seropositive HSCT recipients and CMV-seronegative recipients with a CMV-seropositive donor are considered at risk for CMV disease after HSCT.^26^ Thus, these registries also routinely screen potential donors for CMV serostatus, which can be used as a proxy for CMV infection.

Because of their central role in the immune response to pathogens, we hypothesized that variation in *HLA* genes may impact infection with CMV in healthy individuals. Here, we examine *HLA* variation with respect to serostatus for CMV using high-resolution *HLA* genotyping data from more than 518,000 individuals registered as donors with the NMDP and DKMS stem cell donor registries. Our results show that *HLA* alleles associated with CMV serostatus have effects replicating across both registries and multiple population subgroups, revealing, for the first time, a role for *HLA* variation in susceptibility to CMV infection.

## Methods

### NMDP (discovery) cohort

The discovery cohort includes anonymized data from 366,481 individuals who volunteered as potential hematopoietic stem cell donors at NMDP from January 2018 to February 2023, covering all 50 states in the US. The dataset includes information such as gender, age, self-assigned race (as a proxy for ancestry), NDI (neighborhood deprivation index), CMV serostatus, and high-resolution *HLA* genotyping for all classical class I (*HLA-A* [MIM: 142800], *HLA-B* [MIM: 142830], and *HLA-C* [MIM: 142840]) and class II (*HLA-DRB1* [MIM: 142857] and *HLA-DQB1* [MIM: 604305]) loci. Genotyping was performed through next-generation sequencing (NGS), covering the antigen recognition domain (ARD) exons. The PacBio sequencing platform was used to perform full gene-phased sequencing. Any genotyping ambiguities are addressed through a bioinformatic method aimed at resolving these genotypes.^27^ Four major population subgroups according to self-reported ancestry were included in the study as follows: Black or African American (AFA), Asian or Pacific Islander (API), European descent (EUR), and Hispanic or Latino (HIS). The CMV-seropositive group comprised 115,739 individuals, and the CMV-seronegative group comprised 250,742 individuals, distributed in the four different population groups. Detailed sample sizes are presented in Table 1. The data were deidentified, and their use for this study was approved by the NMDP IRB (institutional review board).

**Table 1:** Sociodemographic characteristics

	**NMDP**			**DKMS**		
	**Case**	**Control**	***p* value**	**Case**	**Control**	***p* value**
**Sex, *n* (%)**						

Female	85,649 (74)	169,924 (67.8)	–	46,837 (72.5)	58,069 (66.2)	–
Male	30,090 (26)	80,818 (32.2)	<0.001^a^	17,773 (27.5)	29,656 (33.8)	<0.001^a^
Age, mean (SD)	30 (7)	27.3 (6.8)	<0.001^b^	33.4 (10.6)	30.8 (10.8)	<0.001^b^

**Subgroup, *n* (%)**						

AFA	5,602 (4.3)	11,150 (4.1)	–	3,263 (4.4)	2,365 (2.4)	–
API	12,696 (9.8)	15,879 (5.8)	–	7,334 (9.9)	3,508 (3.5)	–
EUR	78,056 (60.1)	194,446 (70.7)	–	48,304 (65.2)	77,888 (78.8)	–
HIS	19,385 (14.9)	29,267 (10.6)	–	5,709 (7.7)	3,964 (4)	–

AFA, Black or African American; API, Asian or Pacific Islander; EUR, European descent; HIS, Hispanic or Latino.

a Fisher’s exact test.

b Wilcoxon rank-sum test.

### DKMS (replication) cohort

The replication cohort includes a total of 152,335 individuals registered at DKMS living in the US who volunteered as potential hematopoietic stem cell donors from February 2006 to October 2023. The dataset includes the same variables as the NMDP dataset (deidentified records of gender, age, self-assigned ancestry, NDI, CMV serostatus, and high-resolution *HLA* genotyping for *HLA-A*, *HLA-B*, *HLA-C*, *HLA-DRB1*, and *HLA-DQB1*).^28^^,^^29^ Genotyping ambiguities are treated as described in the NDMP cohort.^27^ Again, the four major population groups were included in the study (AFA, API, EUR, and HIS). The CMV-seropositive group comprised 64,610 individuals, and the CMV-seronegative group comprised 87,725 individuals, distributed in the four different population groups. Detailed sample sizes are presented in Table 1.

### Sociodemographic characteristics

The Fisher’s exact test was used for sex comparisons, the Wilcoxon rank-sum test was applied for comparisons of age and NDI means, and the chi-squared test was performed to compare NDI quintiles between groups on SPSS software (v.20.0). We used the NDI as a proxy measure for SES. NDI is a tool created using factor analysis to identify key variables from 13 measures in the dimensions of SES as follows: wealth and income, education, occupation, and housing conditions.^30^^,^^31^

### *HLA* association analysis

We examined the association of five *HLA* loci (*HLA-A*, *HLA-B*, *HLA-C*, *HLA-DRB1*, *HLA-DQB1*) with CMV serostatus (CMV seropositive versus CMV seronegative) in our discovery cohort. *HLA* data included the first two fields of the allele name as described in the *HLA* nomenclature, which defines HLA allotypes. Only alleles with a frequency greater than 1% were included in the analysis (94 alleles for the AFA subgroup and 97, 108, and 85 for API, HIS, and EUR, respectively). *HLA*-association testing was performed through logistic regression by the glm base package on RStudio^32^ (v.2024.4.2.764), adjusting for sex, age, and NDI for each population subgroup separately. The corrected *p* value for the *HLA* allele association applied for the logistic regression in our discovery dataset was calculated considering the total number of alleles with a frequency greater than 1% for *HLA-A* (14), *HLA-B* (23), and *HLA-DRB1* (21) in our largest population subgroup (EUR) (α = 0.05/58 = 8.62E−04), which considers the strong linkage disequilibrium between some of the loci tested. We repeated the association analysis for alleles significant at the corrected *p* value in the discovery cohort only (55 alleles among all the four population subgroups together) in our replication cohort, considering the significance level at α = 0.05 after applying the Benjamini-Hochberg (FDR [false discovery rate]) correction.

Trans-population meta-analysis was performed for the alleles that were significantly associated with CMV in both the NMDP and DKMS datasets in at least two different populations using the common effect model with the meta package (v.6.5-0)^33^ on RStudio. The meta-analysis was performed using NMDP data. Volcano plots were made using the ggplot2 package (v.3.4.4)^34^ on RStudio.

The analysis of individual *HLA-DRB1* amino acids with CMV serostatus for the four population subgroups was accomplished with the BIGDAWG package (v.3.0.3)^35^ on RStudio.

### Peptide-binding prediction

Peptide-binding prediction analysis included the *HLA-DRB1* alleles showing the strongest protective and risk effects for CMV (*HLA-DRB1^∗^01:03* and *HLA-DRB1^∗^04:03*, respectively) and their more common related alleles that showed no association with the virus (*HLA-DRB1^∗^01:01* and *HLA-DRB1^∗^04:01*). The prediction analysis was performed for the three most immunogenic CMV proteins (pp65, IE-1, and IE-2)^36^ with these four *HLA-DRB1* alleles using NetMHCIIpan 4.1 for HLA class II.^37^ The parameters were set as default, where the peptide is identified as a strong binder (SB) if it is found among the top 2% predicted peptides for the eluted ligand likelihood prediction method. CMV protein sequences were retrieved from the following accession numbers: pp65 (GenBank: AAA45994.1), IE-1 (GenBank: UBQ34175.1), and IE-2 (GenBank: CAG7582958.1).

### Protein structure modeling prediction

The protein structure modeling prediction included the α (*HLA-DRA*) and β (*HLA-DRB1*) chains and the CMV pp65 peptides with the biggest elution ligand (EL) score difference between *HLA-DRB1^∗^01:01* and *HLA-DRB1^∗^01:03* as well as *HLA-DRB1^∗^04:01* and *HLA-DRB1^∗^04:03*. The *HLA-DRB1^∗^01:01*, *HLA-DRB1^∗^01:03*, *HLA-DRB1^∗^04:01*, and *HLA-DRB1^∗^04:03* alleles were included for β chain modeling prediction, which was performed on AlphaFold 3^38^ with the parameters set as default. The electrostatic potential for contacts between α and β chains with the CMV peptide was performed using USCF ChimeraX.^39^

## Results

### CMV seropositivity is correlated with SES and demographics in large HSCT donor registries

Because of the previously documented association of CMV infection/seropositivity with socioeconomic and demographic factors, we first considered these in our analyses. SES can be measured through the NDI, a tool created using factor analysis to identify key variables from 13 measures of the dimensions of SES.^30^^,^^31^ The sociodemographic characteristics of the cohorts can be found in Table 1. As expected, age and the number of females were significantly higher in the case group in both our discovery (NMDP, *N* = 366,481) and replication (DKMS, *N* = 152,335) cohorts. Furthermore, the case group presented a higher frequency in most NDI quintiles than the control group in each of the four population subgroups (AFA, API, EUR, and HIS) tested in both cohorts (Table S1; Figure S1). The AFA subgroup presented higher NDIs in both datasets, while API presented lower NDIs in both cohorts. Thus, we confirmed the importance of these factors in the present cohorts of healthy donors in the US.

### Three *HLA* variants show consistent association with risk or protection in CMV infection

**Figure 1:**
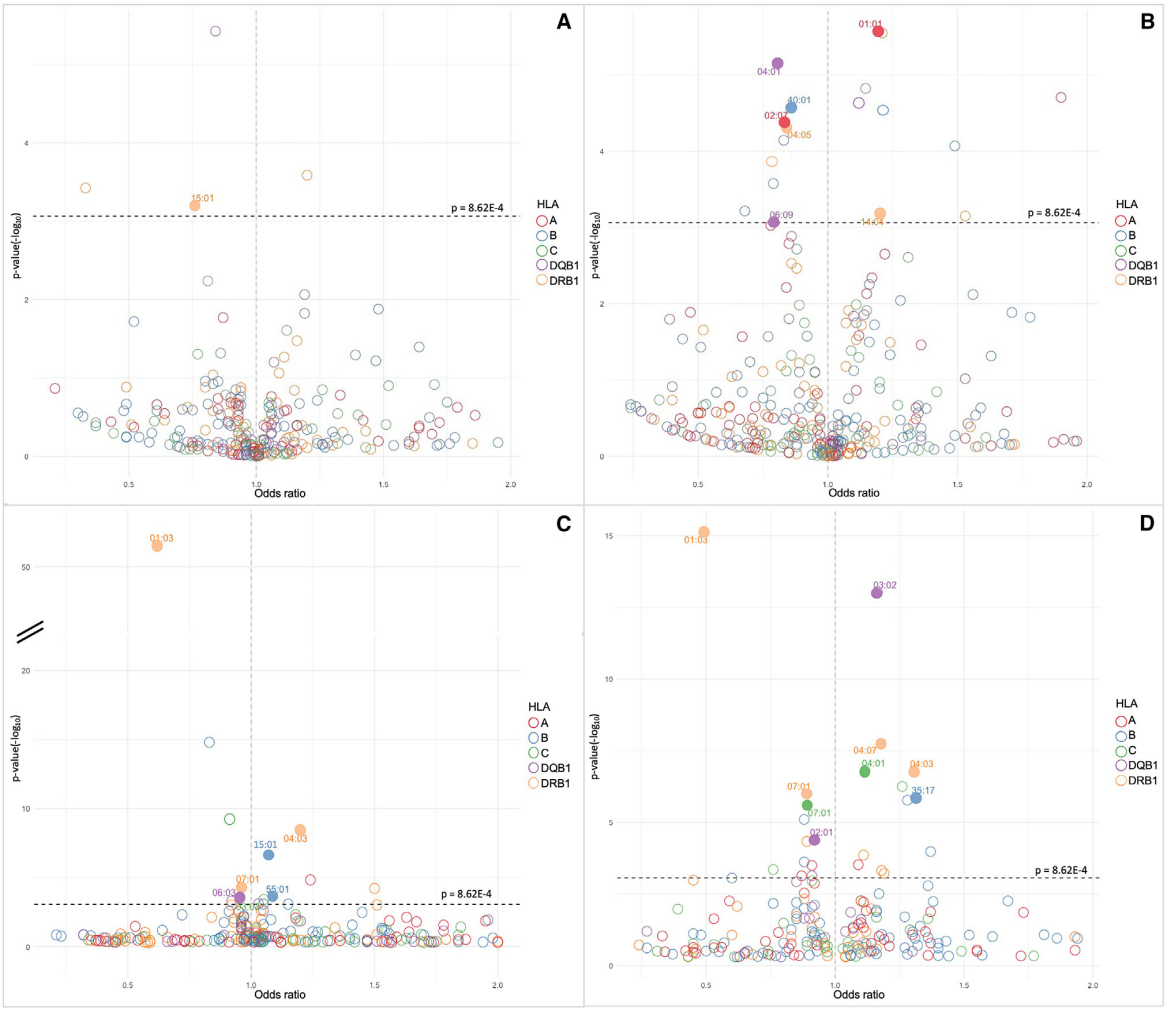
*HLA* alleles associated with CMV in the NMDP cohort Filled circles: *HLA* alleles that significantly replicate in DKMS cohort. *p* value = 8.62E-04 (A) Black or African American. (B) Asian or Pacific Islander. (C) European descent. (D) Hispanic or Latino.

**Figure 2:**
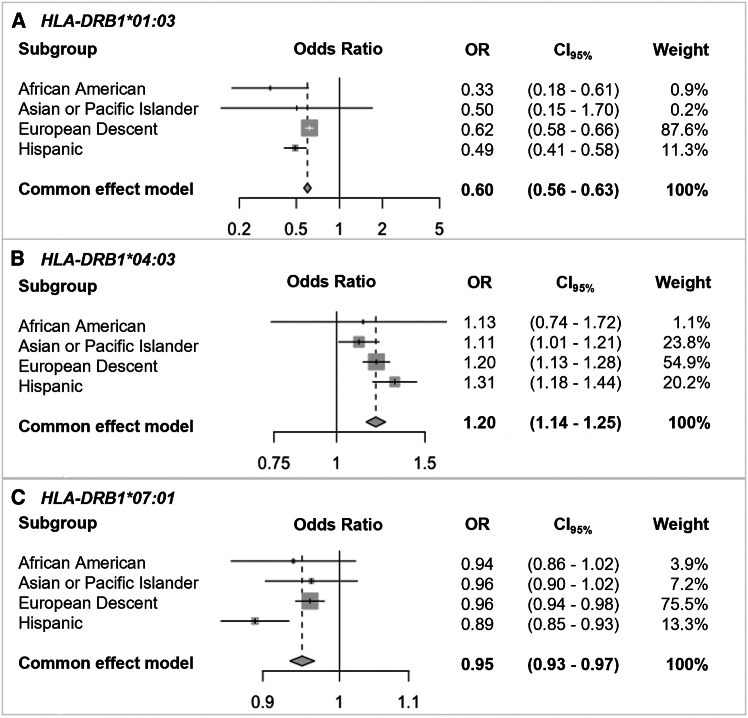
Trans-population meta-analysis forest plot Common effect model *p* value < 0.0001 (A) *HLA-DRB1^∗^01:03*. (B) *HLA-DRB1^∗^04:03*. (C) *HLA-DRB1^∗^07:01*.

We next examined the association of all *HLA* class I and class II alleles with CMV seropositivity through logistic regression controlling for sex, age, and NDI in the four population subgroups separately for both the discovery and replication cohorts. A substantial number of alleles with a frequency > 1% were found to be associated with CMV seropositivity in our discovery (NMDP) cohort that replicated in the DKMS cohort (Tables 2 and S2; Figure 1). Of these, three alleles (*HLA-DRB1^∗^01:03*, *HLA-DRB1^∗^04:03*, and *HLA-DRB1^∗^07:01*) were significantly associated with CMV seropositivity in at least two different population subgroups, and while not reaching statistical significance in other groups, we observed consistent effect sizes across all subgroups considered (Tables 3 and S2). Among those alleles, one was significantly associated with risk (*HLA-DRB1^∗^04:03* in EUR and HIS) and two with protection from CMV infection (*HLA-DRB1^∗^01:03* and *HLA-DRB1^∗^07:01* in EUR and HIS). A trans-population meta-analysis revealed statistically significant associations for all three alleles tested (*p* < 0.001) (Table 4; Figure 2). Alleles *HLA-DRB1^∗^04:03* (odds ratio [OR]: 1.20, 95% confidence interval [95% CI]: 1.14–1.25) and *HLA-DRB1^∗^01:03* (OR: 0.60, 95% CI: 0.56–0.63) showed the strongest common effect sizes for risk and protection, respectively, while the allele *HLA-DRB1^∗^07:01* (OR: 0.95, 95% CI: 0.93–0.97) presented a weaker effect size.

**Table 2:** Significant *HLA* alleles associated with CMV in NMDP that replicate in the DKMS cohort in a single population subgroup

	**NMDP**			**DKMS**		
***HLA* allele**	**OR**	**CI_97.5%_**	***p* value**^a^	**OR**	**CI_97.5%_**	***p* value**^b^
**Black or African American**						

*DRB1^∗^15:01*	0.759	(0.646–0.887)	6.28E−04	0.676	(0.532–0.857)	4.95E−03

**Asian or Pacific Islander**						

*A^∗^01:01*	1.196	(1.110–1.289)	2.66E−06	1.239	(1.106–1.388)	9.99E−04
*A^∗^02:07*	0.833	(0.763–0.909)	4.15E−05	0.749	(0.638–0.880)	1.74E−03
*B^∗^40:01*	0.861	(0.804–0.923)	2.65E−05	0.65	(0.572–0.739)	1.34E−09
*DRB1^∗^14:04*	1.202	(1.081–1.337)	6.68E−04	1.378	(1.177–1.619)	4.52E−04
*DRB1^∗^04:05*	0.841	(0.773–0.914)	4.83E−05	0.636	(0.542–0.746)	3.07E−07
*DQB1^∗^04:01*	0.804	(0.731–0.884)	7.11E−06	0.649	(0.541–0.779)	3.12E−05
*DQB1^∗^06:09*	0.791	(0.689–0.907)	8.36E−04	0.721	(0.573–0.909)	1.62E−02

**European descent**						

*B^∗^55:01*	1.087	(1.040–1.135)	2.29E−04	1.099	(1.035–1.166)	7.15E-03
*B^∗^15:01*	1.072	(1.044–1.100)	2.32E−07	1.076	(1.038–1.115)	3.47E-04
*C^∗^03:03*	1.051	(1.023–1.081)	3.94E−04	1.122	(1.082–1.165)	1.71E−08
*DQB1^∗^06:03*	0.953	(0.928–0.978)	3.18E−04	1.052	(1.015–1.089)	1.62E−02

**Hispanic or Latino**						

*B^∗^35:17*	1.312	(1.175–1.465)	1.37E−06	1.369	(1.080–1.744)	2.79E−02
*C^∗^04:01*	1.116	(1.071–1.163)	1.54E−07	1.118	(1.022–1.224)	3.68E−02
*C^∗^07:01*	0.892	(0.851–0.936)	2.47E−06	0.802	(0.723–0.891)	2.77E−04
*DRB1^∗^04:07*	1.178	(1.112–1.246)	1.78E−08	1.193	(1.058–1.347)	1.43E−02
*DQB1^∗^03:02*	1.164	(1.118–1.211)	9.14E−14	1.195	(1.096–1.303)	3.47E−04
*DQB1^∗^02:01*	0.919	(0.882–0.957)	4.01E−05	0.899	(0.821–0.984)	4.65E−02

Alleles with a frequency higher than 1% are shown. Logistic regression. OR, odds ratio; CI, confidence interval.

a α = 8.62E−04.

b Remained significant after Benjamini-Hochberg (FDR) correction (α = 5.0E−02).

**Table 3:** Significant *HLA* alleles associated with CMV that replicate in at least two population subgroups in both cohorts

	**NMDP**			**DKMS**		
***HLA* allele**	**OR**	**CI_97.5%_**	***p* value**^a^	**OR**	**CI_97.5%_**	***p* value**^b^
***DRB1^∗^01:03***						

AFA	0.328	(0.170–0.587)	3.77E−04^c^	0.702	(0.336–1.477)	1.69E−01
API	0.503	(0.133–1.620)	2.68E−01	0.398	(0.051–2.463)	3.21E−01
EUR	0.618	(0.581–0.657)	2.81E−52^c^	0.669	(0.618–0.725)	3.19E−21^c^^,^^d^
HIS	0.491	(0.412–0.583)	7.26E−16^c^	0.602	(0.419–0.862)	1.62E−02^c^^,^^d^

***DRB1^∗^04:03***						

AFA	1.129	(0.734–1.710)	5.73E−01	4.093	(1.875–10.267)	9.73E−04^c^
API	1.108	(1.010–1.214)	2.96E−02	1.123	(0.958–1.319)	1.56E−01
EUR	1.2	(1.129–1.275)	3.67E−09^c^	1.292	(1.189–1.404)	2.13E−08^c^^,^^d^
HIS	1.306	(1.182–1.443)	1.61E−07^c^	1.316	(1.063–1.636)	3.25E−02^c^^,^^d^

***DRB1^∗^07:01***						

AFA	0.939	(0.862–1.022)	1.46E−01	0.997	(0.864–1.151)	9.64E−01
API	0.962	(0.903–1.025)	2.32E−01	1.055	(0.953–1.169)	3.00E−01
EUR	0.96	(0.942–0.979)	5.05E−05^c^	0.968	(0.942–0.994)	3.85E−02^c^^,^^d^
HIS	0.89	(0.850–0.933)	1.01E−06^c^	0.819	(0.738–0.909)	8.48E−04^c^^,^^d^

Logistic regression. OR, odds ratio; CI, confidence interval; AFA, Black or African American; API, Asian or Pacific Islander; EUR, European descent; HIS, Hispanic or Latino.

a α = 8.62E−04.

b α = 5.0E−02.

c Significant values.

d Remained significant after Benjamini-Hochberg (FDR) correction.

**Table 4:** Trans-population meta-analysis

	**Common effect model**				**Weight (%)**				**Heterogeneity**		
	**OR**	**CI_95%_**	**z**	***p* value**	**AFA**	**API**	**EUR**	**HIS**	***I*2 (%)**	**t2**	***p* value**
*HLA-DRB1^∗^01:03*	0.599	0.565–0.634	−17.34	<0.0001	0.90	0.20	87.60	11.30	69.40	0.033	0.02
*HLA-DRB1^∗^04:03*	1.197	1.144–1.252	7.85	<0.0001	1.10	23.80	54.90	20.20	47.70	0.003	0.125
*HLA-DRB1^∗^07:01*	0.95	0.934–0.966	−5.92	<0.0001	3.90	7.20	75.50	13.30	66.20	0.001	0.031

OR, odds ratio; CI, confidence interval; AFA, Black or African American; API, Asian or Pacific Islander; EUR, European descent; HIS, Hispanic or Latino.

Further, we observed a strong dose effect for these alleles (Table S3). For example, the risk associated with two copies of *HLA-DRB1^∗^04:03* was almost three times higher in EUR (OR = 3.083, 97.5% CI = 1.642–5.874) than a single copy. Likewise, *HLA-DRB1^∗^01:03* homozygosity showed a protective effect almost three times higher than heterozygotes in the EUR subgroup (OR = 0.259, 97.5% CI = 0.076–0.661).

In summary, we observed clear evidence of *HLA* involvement in both risk and protection in CMV infection, with results replicating across independent registries and subpopulations.

### Numerous *HLA* alleles show ancestry-specific association with CMV seropositivity

In addition to the three alleles replicating in multiple population subgroups, we observed a number of other alleles associated with CMV seropositivity in only a single population subgroup in our discovery (NMDP) cohort that replicated in the DKMS cohort (Table 2; Figure 1). The API subgroup presented the highest number of alleles associated with CMV (seven alleles, two risk), with the allele *HLA-DRB1^∗^14:04* showing the largest risk effect size (OR = 1.202, 97.5% CI = 1.081–1.337, *p* = 6.68E−04) and the allele *HLA-DQB1^∗^06:09* showing the largest protection effect size (OR = 0.791, 97.5% CI = 0.689–0.907, *p* = 8.36E−04). However, neither of these alleles were found to be associated with CMV serostatus in the other population subgroups evaluated. The HIS subgroup presented six associated alleles (four risk), as the biggest effect sizes were observed in the alleles *HLA-B^∗^35:17* (OR = 1.312, 97.5% CI = 1.175–1.465, *p* = 1.37E−06) and *HLA-C^∗^07:01* (OR = 0.892, 97.5% CI = 0.851–0.936, *p* = 2.47E−07) for risk and protection, respectively. In the EUR subgroup, four alleles were found to be associated with CMV serostatus (three risk), where *HLA-B^∗^55:01* (OR = 1.087, 97.5% CI = 1.040–1.135, *p* = 2.29E−04) presented the largest risk effect size and *HLA-DQB1^∗^06:03* (OR = 0.953, 97.5% CI = 0.928–0.978, *p* = 3.18E−04) presented the largest protective effect. Interestingly, only *HLA-DRB1^∗^15:01* (OR = 0.759, 97.5% CI = 0.646–0.887, *p* = 6.28E−04) was associated with CMV serostatus in the AFA subgroup.

In summary, beyond the four HLA alleles that showed consistent results across ancestries, we find extensive evidence of additional ancestry-specific *HLA* involvement in CMV serostatus in healthy individuals, which was replicated in independent cohorts.

### *HLA* alleles associated with CMV seropositivity are predicted to have distinct peptide binding repertoires

**Figure 3:**
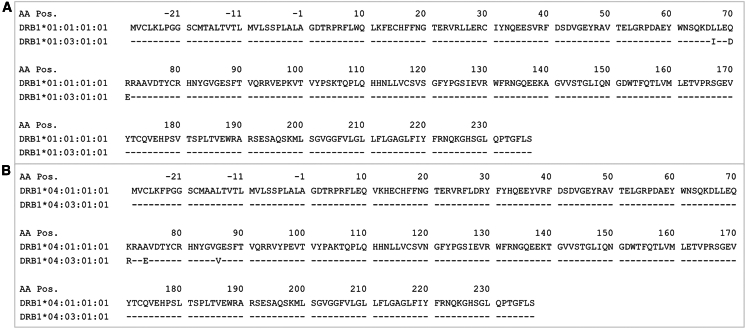
*HLA-DRB1* protein sequence alignment (A) *HLA-DRB1^∗^01:01* and *HLA-DRB1^∗^01:03*. Differences in residues 67, 70, and 71 are shown. (B) *HLA-DRB1^∗^04:01* and *HLA-DRB1^∗^04:03*. Differences in amino acid residues 71, 74, and 86 are shown. aa, amino acid.

We next sought to examine whether predicted patterns of peptide binding might explain some of the observed associations of *HLA* variation with CMV serostatus. Because *HLA-DRB1^∗^01:03* and *HLA-DRB1^∗^04:03* showed the strongest protective and risk effects, respectively, and replicated across population subgroups, we chose to focus on these alleles. These two alleles with opposite effects are members of different allelic families of *HLA-DRB1* and differ at 20 amino acid residues in the mature protein.^40^ Interestingly, despite having a highly significant effect, each of these alleles is found at a low frequency (∼1%) in our US EUR populations. Meanwhile, their highly homologous counterparts, *HLA-DRB1^∗^01:01* and *HLA-DRB1^∗^04:01*, are relatively common, each with frequencies of around 8% in the same population. Of note, there are only three amino acid differences between the mature proteins of *HLA-DRB1^∗^01:01* and *HLA-DRB1^∗^01:03* (residues 67, 70, and 71) and, likewise, *HLA-DRB1^∗^04:01* and *HLA-DRB1^∗^04:03* (residues 71, 74, and 86) (Figure 3).

To clarify the role of these individual amino acid differences in CMV infection, we examined the association of *HLA-DRB1* amino acids with CMV serostatus for the four population subgroups. Numerous *HLA-DRB1* amino acid residues showed an association with CMV seropositivity in the four population subgroups in our discovery cohort (Table S4). However, no individual amino acid demonstrated stronger effect sizes than those observed for the *HLA* allotypes, pointing to structural differences determined by the combination of these amino acid differences.

Thus, we were interested in understanding differential peptide binding between allotypes encoded by these relatively rare alleles that were strongly associated with CMV and those encoded by their closely related, more common alleles that showed no association with the virus. We predicted peptide binding for the three most immunogenic CMV proteins (pp65, IE-1, and IE-2)^36^ with these four *HLA-DRB1* alleles using NetMHCIIpan 4.1 for *HLA* class II.^37^ Notably, despite their overall similarity, the *HLA* allotypes associated with CMV seropositivity had peptide-binding predictions that were distinct from the closely related, non-associated allotypes (Table S5). For example, considering only the SB peptides that are not shared with the other *HLA* alleles included in this comparison, four pp65 peptides were predicted as SBs specifically for the *HLA-DRB1^∗^01:03* allotype, but *HLA-DRB1^∗^01:01* showed no SB peptides for this CMV protein. On the other hand, two unique IE-1 SB peptides were predicted for *HLA-DRB1^∗^01:01* but none for *HLA-DRB1^∗^01:03*, while for protein IE-2, there were three and two distinct SB peptides predicted for *HLA-DRB1^∗^01:01* and *HLA-DRB1^∗^01:03*, respectively. Therefore, *HLA-DRB1^∗^01:03* and *HLA-DRB1^∗^01:01* do not share SB peptides for pp65 and IE-1 CMV proteins. In turn, *HLA-DRB1^∗^04:03* showed ten, nine, and eight exclusive SB peptides for pp65, IE-1, and IE-2 CMV proteins, respectively, while *HLA-DRB1^∗^04:03* presented only three unique SB peptides for pp65 only and non-unique SBs for IE-1 and IE-2 CMV proteins. Strikingly, despite their similarity, for each protein considered, the number of overlapping SBs for each pair of alleles was always fewer than those that were specific to one allele or the other (Figure S2). Thus, it is clear that despite only three amino acid differences, the CMV-associate allotypes are predicted to have largely divergent peptide binding profiles relative to their more common counterparts for immunodominant CMV proteins.

### Polymorphisms in *HLA-DRB1* binding pockets P4 and P7 are associated with CMV seropositivity

**Figure 4:**
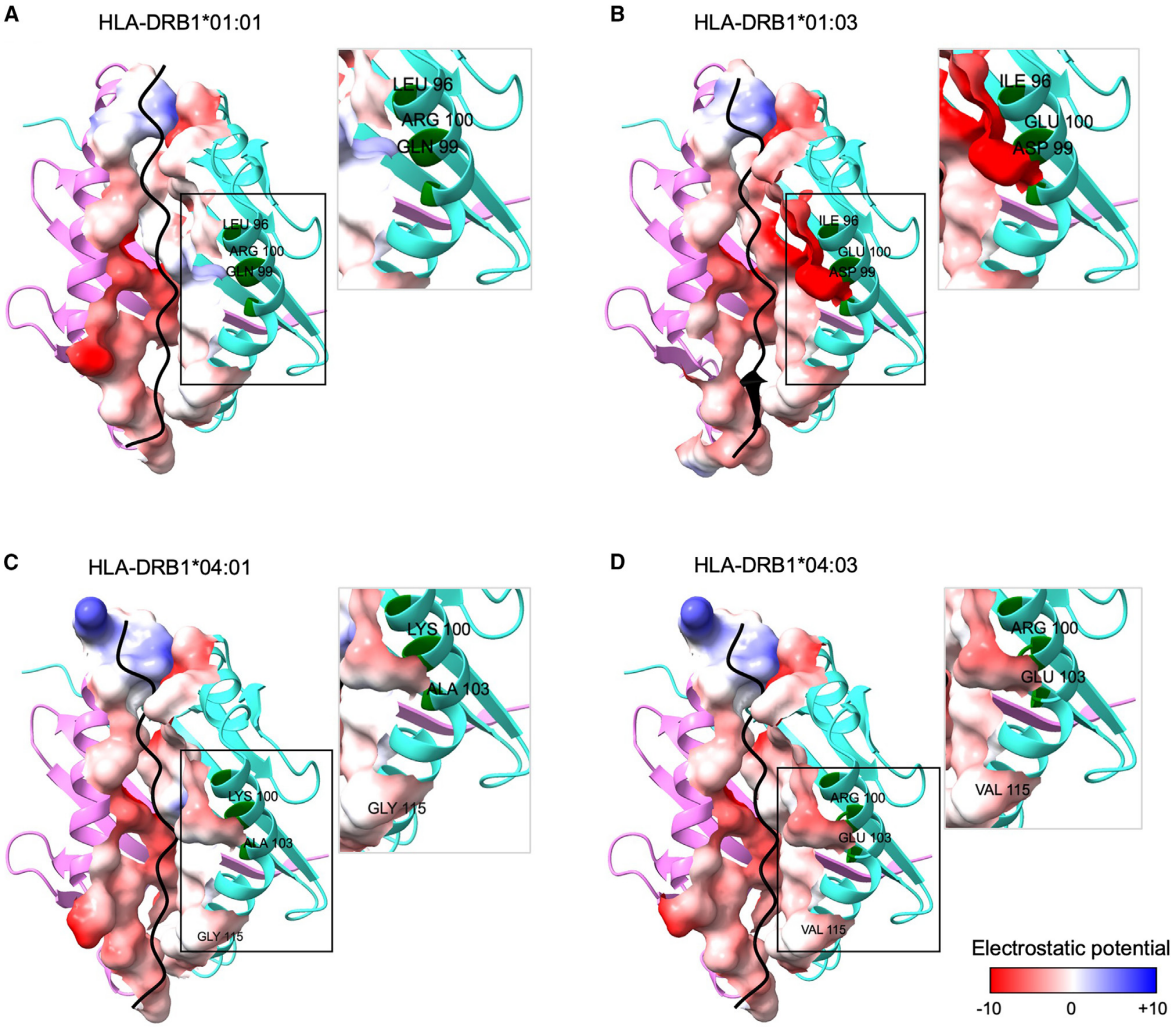
*HLA-DRB1* binding groove *HLA-DRB1* binding groove and CMV pp65 DTPVLPHETRLLQTG peptide for *HLA-DRB1^∗^01:01* and *HLA-DRB1^∗^01:03* (A and B) and pp65 VSQYTPDSTPCHRGD peptide for *HLA-DRB1^∗^04:01* and *HLA-DRB1^∗^04:03* (C and D). (A) *HLA-DRB1^∗^01:01*. (B) *HLA-DRB1^∗^01:03*. (C) *HLA-DRB1^∗^04:01*. (D) *HLA-DRB1^∗^04:03*. α chain: violet. β chain: turquoise. CMV pp65 peptides: black. Blue: positive charges. White: neutral charges. Red: negative charges. Variant peptides between *HLA-DRB1^∗^01:01* and *HLA-DRB1^∗^01:03* as well as *HLA-DRB1^∗^04:01* and *HLA-DRB1^∗^04:03* are highlighted in green and labeled.

We next sought to model these closely related (yet discordant with respect to their association with infection) pairs of *HLA-DRB1* alleles binding to CMV peptides to understand how their amino acid differences impact the electrostatic potential in the peptide-binding groove. Our modeling predictions included the α (*HLA-DRA*) and β (*HLA-DRB1*) chains and the CMV pp65 peptides with the biggest EL score difference between *HLA-DRB1^∗^01:01* and *HLA-DRB1^∗^01:03* as well as *HLA-DRB1^∗^04:01* and *HLA-DRB1^∗^04:03* (Figure 4; Table S5). The electrostatic potential for contacts between α and β chains with the CMV peptides is different for the four aforementioned *HLA-DRB1* alleles. The three residue differences between *HLA-DRB1^∗^01:03* and *HLA-DRB1^∗^01:01* (positions 67, 70, and 71) lie in the hypervariable region 3^41^ (HVR3; ranging from 67 to 74 residues) of the *HLA-DRB1* gene, while two out of the three different amino acid residues between *HLA-DRB1^∗^04:03* and *HLA-DRB1^∗^04:01* (71 and 74) lie in HVR3 (Figures 3 and 4). In antigen presentation to T cells, the peptide is bound within a groove on the surface of the HLA protein. The peptide-binding groove of *HLA* class II molecules consists of nine different structural pockets (P1–P9), which accommodate the antigen peptide side chains.^42^^,^^43^ The amino acid differences between *HLA-DRB1^∗^01:01* and *HLA-DRB1^∗^01:03* occur on binding pockets P4 (residues 70 and 71)^43^ and P7 (residues 67 and 71).^43^ Notably, for alleles *HLA-DRB1^∗^04:03* and *HLA-DRB1^∗^04:01*, the differences occur in the same P4 (residues 71 and 74)^43^ and P7 (residue 74)^43^ pockets.

These differences impact the charge and/or the volume of the molecule. For instance, the *HLA-DRB1^∗^01:01* allele codes for glutamine at residue 70 (uncharged, medium volume of 143.8 Å^3^),^44^^,^^45^ while the *HLA-DRB1^∗^01:03* allele codes for aspartic acid at residue 70 (negative charge, small volume of 111.1 Å^3^).^44^^,^^45^ For residue 71, *HLA-DRB1^∗^01:01* codes for arginine (positive charge, large volume of 173.4 Å^3^),^44^^,^^45^ while *HLA-DRB1^∗^01:03* codes for glutamic acid (negative charge, medium volume of 138.3 Å^3^).^44^^,^^45^ Likewise, for the *HLA-DRB1^∗^04:03* and *HLA-DRB1^∗^04:01* alleles, residue 74 presents the biggest difference in terms of charge and volume, coding for alanine (uncharged, very small volume of 88.6 Å^3^)^44^^,^^45^ in *HLA-DRB1^∗^04:01* and for glutamic acid (negative charge, medium volume of 138.3 Å^3^)^44^^,^^45^ in *HLA-DRB1^∗^04:03*. Overall, only *HLA-DRB1^∗^01:03* results in a negative charge at residue 71, while all other alleles tested here present a positive charge (Table S6), impacting in the electrostatic potential in the binding pockets P4 and P7 (Figure 4B).

In conclusion, we find support for structural differences between rare CMV-associated and common non-associated *HLA* allotypes that may explain differences in binding to CMV-derived peptides.

## Discussion

Uncontrolled CMV replication in immunocompromised patients can lead to end-organ diseases such as pneumonitis, retinitis, hepatitis, or gastroenteritis. CMV infection in solid organ transplant patients can lead to either direct effects, such as end-organ disease, or indirect effects, including bacterial and fungal coinfections^24^^,^^46^ and GVHD, in HSCT patients.^25^ In addition, CMV is associated with poor transplantation outcomes that include higher mortality.^23^ Furthermore, congenital CMV infection is the most common congenital infection in industrialized countries.^47^^,^^48^ Congenital CMV infection is neglected mainly because most maternal and newborn infections are asymptomatic, and sequelae from congenital CMV infection are frequently delayed in onset, making retrospective diagnosis difficult.^5^ The socioeconomic impact of CMV as the commonest nongenetic cause of childhood hearing loss and a significant cause of neurodevelopmental delay is underappreciated.^48^ Symptomatic neonates can develop hearing loss and cognitive and motor delay as permanent sequelae.^49^ As CMV is the most common intrauterine infection, effective vaccine development is a high priority for the protection of both newborns and transplant recipients.^49^^,^^50^^,^^51^

Understanding the genetic and immunological underpinnings of CMV infection is important for public health measures, vaccine design, and therapeutic development. Leveraging two extremely large cohorts, we were able to identify *HLA* alleles associated with both risk and protection for CMV serostatus among four different broad US population subgroups. Revealing *HLA* alleles associated with CMV can provide important insights into the immunological response to CMV infection. To the best of our knowledge, this retrospective case-control study represents the most well-powered examination of *HLA* and CMV to date, as well as one of the few in an immunocompetent population, including high-resolution *HLA* genotyping and CMV serostatus data, as well as important demographic and socioeconomic variables, from more than a half-million individuals.

Twenty-one *HLA* alleles were significantly associated with CMV serostatus in both our discovery and replication cohorts, most with relatively small effect sizes. Focusing on those alleles that were significantly associated with CMV in at least two different population subgroups, we highlight the role of three class II alleles (*HLA-DRB1^∗^01:03*, *HLA-DRB1^∗^04:03*, and *HLA-DRB1^∗^07:01*). A common effect trans-population meta-analysis showed effect sizes consistently higher for the highlighted alleles than those that did not replicate across multiple ancestries, where *HLA-DRB1^∗^01:03* showed the strongest protective effect size and *HLA-DRB1^∗^04:03* presented the strongest risk effect, with evidence for dose effects. Of particular interest is the fact that while both alleles are found at a low frequency in our largest population subgroup (EUR), their closely related counterparts *HLA-DRB1^∗^01:01* and *HLA-DRB1^∗^04:01* are quite common and yet show no association with CMV serostatus.

All three different amino acid residues between *HLA-DRB1^∗^01:03* and *HLA-DRB1^∗^01:01* lie in the HVR3 of the *HLA-DRB1* gene, while two out of the three different amino acid residues lie in HVR3 in relation to *HLA-DRB1^∗^04:03* and *HLA-DRB1^∗^04:01* alleles. Differences in these polymorphic residues can impact the charge and/or the volume of the molecule as is shown in Figure 4. The polymorphic residue 71 is part of both pockets P4 and P7 and codes for a negatively charged amino acid (glutamic acid) in the *HLA-DRB1^∗^01:03* allele, while the other three *HLA-DRB1* alleles code for a positively charged amino acid. The structural pockets of the *HLA* class II binding groove exert a major influence on peptide binding and its recognition by T cells.^52^ The polymorphic residues 70, 71, and 74 of the *HLA-DRB1* protein, located in pocket P4 of the β chain, play a central role in the recognition of the *HLA-DRB1*/peptide complex by the CD4^+^ helper T cell.^53^ Thus, it seems that particular features of these alleles, which differ by only three amino acids from their close relatives, are responsible for their relationship with CMV infection.

Rovito et al. evaluated the CMV viral load in children affected by congenital CMV. In their study, the median viral load was significantly lower in the children carrying *HLA-DRB1^∗^04* compared to the *HLA-DRB1^∗^04*-negative children, indicating a protective role against CMV.^54^ However, the genotyping resolution in that study would not have detected the differential association between *HLA-DRB1^∗^04:01* (neutral) and *HLA-DRB1^∗^04:03* (risk) reported here. In other disease contexts, different associations of these closely related alleles have been shown. For example, *HLA-DRB1^∗^04:01* confers risk for type 1 diabetes (MIM: 222100), while *HLA-DRB1^∗^04:03* confers protection.^55^ Our own work has shown that *HLA-DRB1^∗^04:01* is protective in Parkinson disease (MIM: 168600), while *HLA-DRB1^∗^04:03* is not,^56^ as part of a pattern related to the *HLA-DRB1* “shared epitope” (SE). The SE was first recognized due to an association between a five-amino-acid sequence motif in residues 70–74 of *HLA-DRB1* alleles with severe rheumatoid arthritis (RA [MIM: 180300]),^57^^,^^58^ with the *HLA-DRB1^∗^04* allelic group representing the most common SE-coding alleles.^59^ Interestingly, two out the three amino acid differences (residues 71 and 74) between *HLA-DRB1^∗^04:01* (QKRAA, SE positive) and *HLA-DRB1^∗^04:03* (QRRAE, SE negative) lie in the SE region. While CMV infection has not been related as a trigger for RA, there is a possible association between CMV and the pathophysiology of RA, being an aggravating factor of inflammation in RA while protecting from bone erosion.^60^ Interaction between SE and low levels of both anti-EBV (Epstein-Barr virus) and anti-B19 (parvovirus B19) antibodies has also been demonstrated.^61^

Similarly, two amino acid differences (residues 70 and 71) between *HLA-DRB1^∗^01:01* (QRRAA, SE positive) and *HLA-DRB1^∗^01:03* (DERAA, SE negative) alleles lie in the SE region. *HLA-DRB1^∗^01:03* has been shown to be associated with risk for Crohn disease (MIM: 266600) and ulcerative colitis (MIM: 191390) in several large studies of individuals with inflammatory bowel disease (IBD).^62^ The authors suggest that this allele is critically involved in determining the colonic immune response to local flora.^62^ Here, we demonstrated, for the first time, a protective effect of the *HLA-DRB1^∗^01:03* allele against CMV infection. While there is no clear evidence for a role of CMV infection across autoimmune diseases, it has been suggested that CMV can trigger systemic lupus erythematosus (SLE [MIM: 152700])^63^^,^^64^ or aggravate the disease.^65^ However, it is not clear whether CMV infection triggers SLE or occurs simultaneously with or after SLE onset.^66^ Nevertheless, there is some evidence for an intriguing interplay between adaptive immune responses to CMV and manifestations of certain autoimmune disorders, for example, in RA and SLE.^67^

The CMV proteins pp65, IE-1, and IE-2 are the major targets of the cellular immune response.^36^ In our peptide-binding prediction analysis, IE-2 showed a higher number of SB peptides among the CMV proteins tested with the four *HLA-DRB1* allotypes, and IE-1 presented the lowest number. Interestingly, the strongest protective *HLA-DRB1^∗^01:03* allele in our analysis showed no SB peptides for IE-1 protein. The peptides IE-2_408–422_ (KGIQIIYTRNHEVKS) and IE-2_438–452_ (ALSTPFLMEHTMPVT), previously validated for *HLA-DRB1^∗^07:01* and *HLA-DRB1^∗^01:01* restriction, respectively,^68^ were predicted as SBs for *HLA-DRB1^∗^01:03* in our analysis. Slezak et al. characterized several pp65 peptides in 20 healthy CMV-seropositive subjects,^69^ but none of the peptides overlap with the SB peptides predicted in our analysis. The subjects included in their study presented diverse *HLA-DRB1* allotypes, which in part explain the different peptides predicted in our study. In their study, no IE-1 class II epitopes were identified,^69^ while in our prediction analysis, IE-1 presented the lowest number of SBs—and no SBs for *HLA-DRB1^∗^01:03*. The lack of naturally occurring CD4^+^ T cell responses to IE-1 suggests that its epitopes may not be good candidates to trigger immune response.^69^ In another study including individuals with diverse *HLA* alleles, CMV pp65 peptide-specific T cell lines selectively respond to a restricted number of CMV pp65 epitopes presented by a limited number of prevalent *HLA* alleles.^70^ For instance, the epitopes presented by *HLA-B^∗^07:02* and *HLA-A^∗^02:01* alleles consistently elicit immunodominant CMV pp65 peptide-specific T-cells.^70^ Yet, all immunodominant CMV pp65 peptide-specific T cells exhibiting *HLA*-restricted cytotoxicity against epitope-loaded targets, except those responding to epitopes presented by *HLA-B^∗^35* alleles, were ineffective in controlling CMV infections.^70^ Identifying immunodominant epitopes in major immunogenic CMV proteins and their *HLA* allelic restrictions could be useful for adoptive immune therapy and vaccine development.^71^

As a potential limitation of the study, we acknowledge that our reliance on self-reported ancestry may be less ideal than using genome-wide data to assess population substructure, for instance. However, we also note that we have addressed the question of the accuracy of self-reported ancestry in the NMDP cohort (discovery) in four published studies^72^^,^^73^^,^^74^^,^^75^ that included consideration of genome-wide markers. While imperfect, we found that self-reported identification is a reasonable proxy for *HLA* genetic variation in this cohort, and these self-reported categories are fundamental to the matching algorithms used to locate suitable *HLA* matches for patients from among the millions of registered donors. We also note that we only report associations that replicated in a completely independent cohort from a second donor registry with different recruitment processes and demographic makeups, which we feel minimizes the likelihood that the reported associations are spurious or related to underlying population substructures.

An additional limitation is the lower rates of CMV seropositivity in our cohorts relative to the general US population, which may limit its generalizability. We note that several factors in donor recruitment may be responsible for this differential: donors are, on average, younger, healthier, and have higher SES status than the general population. Overall, recruitment is skewed toward young males (and European ancestry, despite efforts to improve diversity), who are known to have a much lower CMV-positive rate in all three dimensions (age, sex, and ancestry).^76^

In summary, we present highly significant associations of *HLA* class I and class II alleles with CMV serostatus in two large cohorts of healthy people. Our results contribute to our understanding of the role of *HLA* variation in CMV infection and may provide a basis for vaccine development or therapeutic targets in the future.

## Data and code availability

The full raw data that support the findings of this study are available from the corresponding author upon reasonable request. Data are located in controlled access data storage at the University of California, San Francisco.

## Acknowledgments

The authors wish to thank Rachel Rutihauser for helpful discussions. We also thank the volunteer donors in the NMDP and DKMS registries. This work was supported by 10.13039/100000002NIH R01AI158861 (J.A.H.).

## Author contributions

J.A.H. and M.M. conceived this work; J.A.B. and J.A.H. undertook the formal analysis and investigation and wrote the original draft; J.S., A.H.S., and M.M. undertook the dataset collection and curation; J.A.H. obtained resources, conducted project administration, and supervised the study; and all authors reviewed and edited the final manuscript.

## Declaration of interests

The authors declare no competing interests.

## Web resources

GenBank, https://www.ncbi.nlm.nih.gov/genbank/
OMIM, https://www.omim.org

## Supplemental information

Document S1. Figures S1 and S2 and Tables S1–S6

Document S2. Article plus supplemental information
