# Supplementary material for: Human leukocyte antigen variation is associated with cytomegalovirus serostatus in healthy individuals
Source: Am J Hum Genet. 2025 Mar 5;112(4):913–26. doi: 10.1016/j.ajhg.2025.02.007 (PMC12081270; doi:10.1016/j.ajhg.2025.02.007)
Supplement: Document S1. Figures S1 and S2 and Tables S1–S6 [file mmc1.pdf]

The American Journal of Human Genetics, Volume *112*

Supplemental information

Human leukocyte antigen variation is associated with cytomegalovirus serostatus in healthy individuals

Juliano A. Boquett, Jürgen Sauter, Alexander H. Schmidt, Martin Maiers, and Jill A. Hollenbach

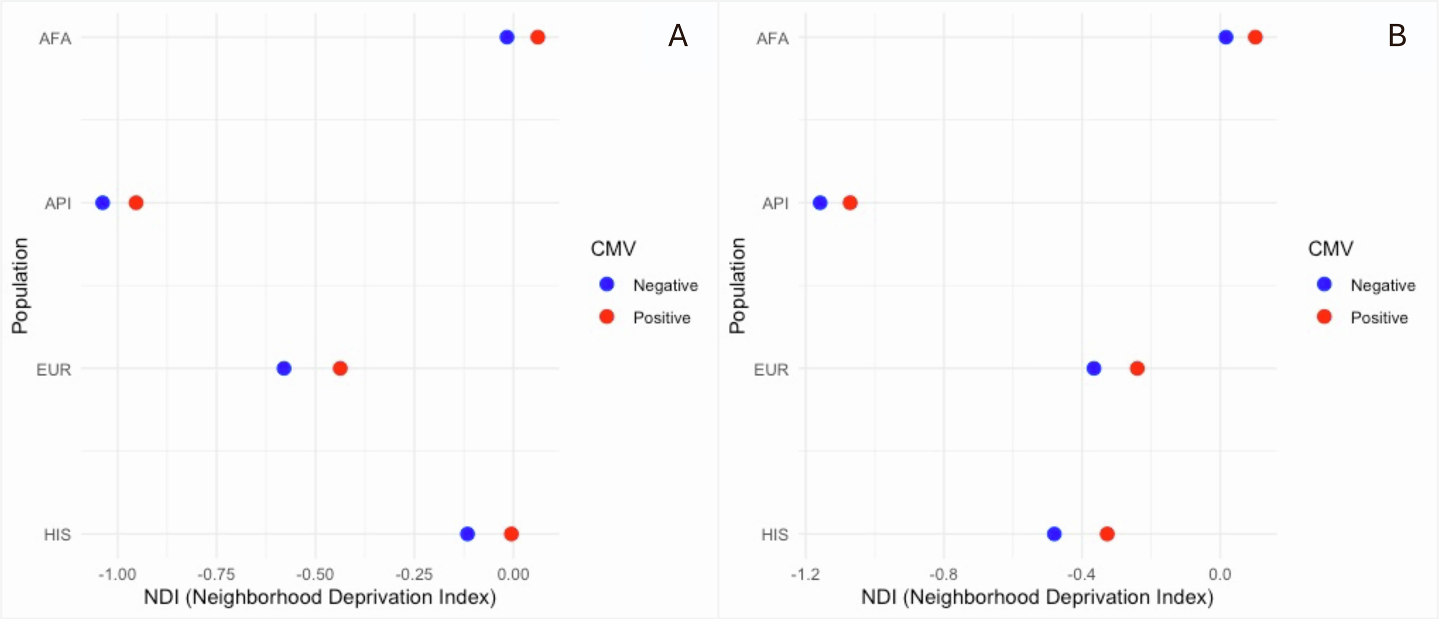

Figure S1. NDI (neighborhood deprivation index) mean among population subgroups. **A)** NMDP cohort. **B)** DKMS cohort. AFA: Black or African American; API: Asian or Pacific Islander; EUR: European descent; HIS: Hispanic or Latino. p-value < 0.05 in each population subgroup in both cohorts (Wilcoxon Rank Sum test).

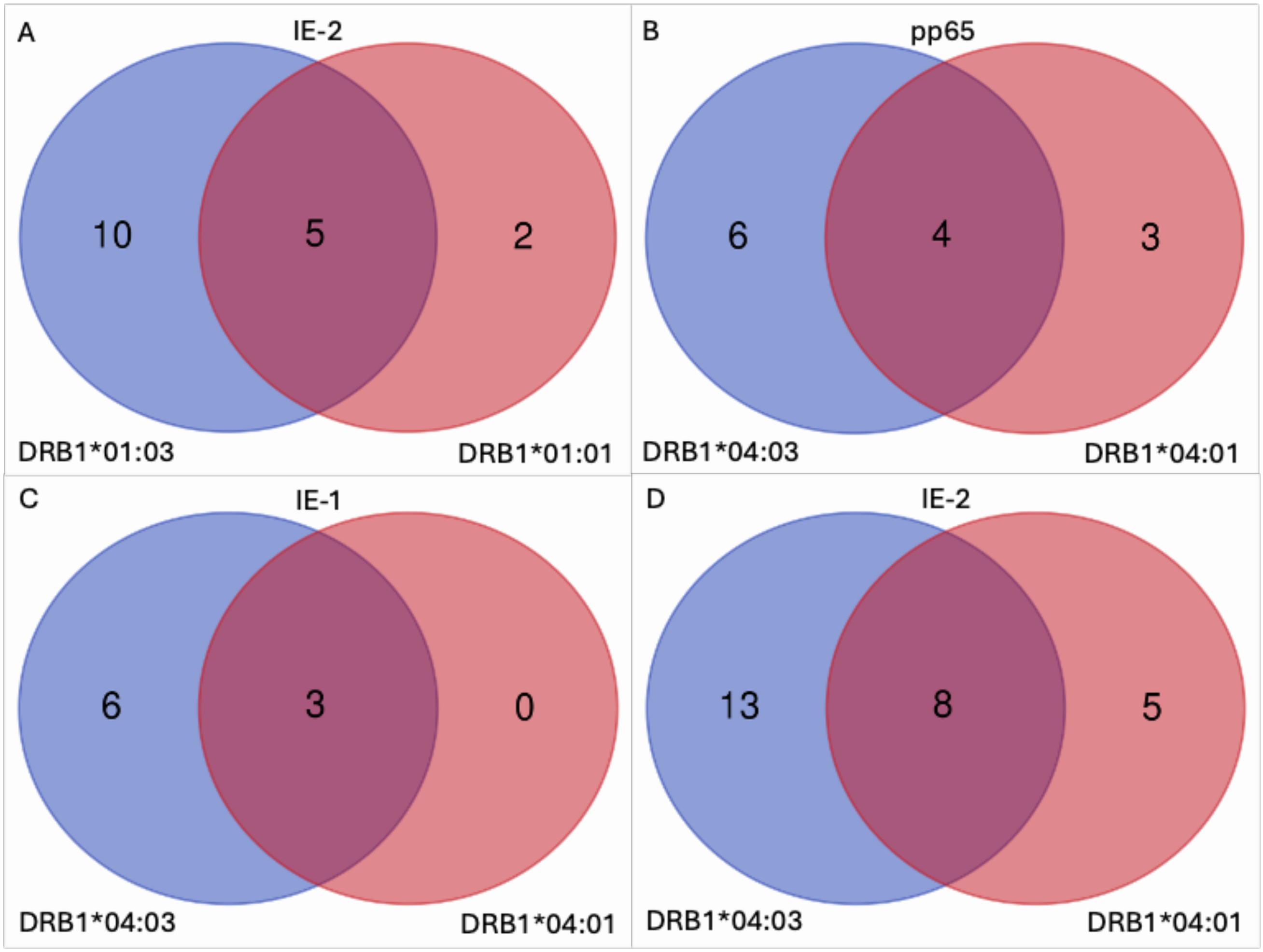

Figure S2. Venn diagrams for stronger binder (SB) peptides shared between *HLA-DRB1* alleles and CMV proteins. **A)** Number of SB for IE-2 protein and *HLA-DRB1*01:03* and *HLA-DRB1*01:01* alleles. **B)** Number of SB for pp65 protein and *HLA-DRB1*04:03* and *HLA-DRB1*04:01* alleles. **C)** Number of SB for IE-1 protein and *HLA-DRB1*04:03* and *HLA-DRB1*04:01* alleles. **D)** Number of SB for IE-2 protein and *HLA-DRB1*04:03* and *HLA-DRB1*04:01* alleles. *HLA-DRB1*01:01* showed no SB for pp65 protein and *HLA-DRB1*01:03* showed no SB for IE-1 protein.

Table S1. NDI quintiles distribution among population subgroups

NDI quintile	NMDP			DKMS		
	**Case** [n (%)]	**Control** [n (%)]	**p-value***	**Case** [n (%)]	**Control** [n (%)]	**p-value***
Black or African American						
1-Least deprivation	998 (17.8)	2208 (19.8)		280 (8.6)	247 (10.4)	
2-Below avg deprivation	1201 (21.4)	2476 (22.2)		479 (14.7)	373 (15.8)	
3-Average deprivation	1067 (19)	2259 (20.3)		581 (17.8)	423 (17.9)	
4-Above avg deprivation	1130 (20.2)	2086 (18.7)		738 (22.6)	535 (22.6)	
5-Most deprivation	1206 (21.5)	2121 (19)	<0.001	1185 (36.3)	787 (33.3)	<0.001
Asian or Pacific Islander						
1-Least deprivation	6919 (54.5)	9110 (57.4)		3246 (44.3)	1658 (47.3)	
2-Below avg deprivation	2929 (23.1)	3590 (22.6)		1724 (23.5)	835 (23.8)	
3-Average deprivation	1587 (12.5)	1767 (11.1)		1133 (15.4)	473 (13.5)	
4-Above avg deprivation	816 (6.4)	955 (6)		741 (10.1)	343 (9.8)	
5-Most deprivation	445 (3.5)	457 (2.9)	<0.001	490 (6.7)	199 (5.7)	<0.001
European descent						
1-Least deprivation	25306 (32.4)	73037 (37.6)		8128 (16.8)	16319 (21)	
2-Below avg deprivation	19881 (25.5)	50919 (26.2)		9349 (19.4)	16628 (21.3)	
3-Average deprivation	15353 (19.7)	35149 (18.1)		9839 (20.4)	16055 (20.6)	
4-Above avg deprivation	11439 (14.7)	24001 (12.3)		10321 (21.4)	15418 (19.8)	
5-Most deprivation	6077 (7.8)	11340 (5.8)	<0.001	10667 (22.1)	13468 (17.3)	<0.001
Hispanic or Latino						
1-Least deprivation	4010 (20.7)	7105 (24.3)		500 (8.8)	524 (13.2)	
2-Below avg deprivation	4073 (21)	6589 (22.5)		759 (13.3)	628 (15.8)	
3-Average deprivation	3677 (19)	5310 (18.1)		987 (17.3)	746 (18.8)	
4-Above avg deprivation	3495 (18)	4647 (15.9)		1314 (23)	832 (21)	
5-Most deprivation	4130 (21.3)	5616 (19.2)	<0.001	2149 (37.6)	1234 (31.1)	<0.001

*Chi-square test.

Table S2. Frequency of significant *HLA* alleles associated with CMV in the NMDP cohort that replicates in the DKMS cohort

	**NMDP - N (Freq%)**		**DKMS - N (Freq %)**	
***HLA* Allele**	**Control**	**Case**	**Control**	**Case**
Black or African American				
*DRB1*15:01*	643 (2.9)	248 (2.2)	162 (3.4)	157 (2.4)
Asian or Pacific Islander				
*A*01:01*	1890 (6)	1662 (6.5)	562 (8)	1425 (9.7)
*A*02:07*	1464 (4.6)	1023 (4)	297 (4.2)	428 (2.9)
*B*40:01*	2473 (7.8)	1768 (7)	511 (7.3)	683 (4.7)
*DRB1*14:04*	834 (2.6)	745 (2.9)	236 (3.4)	687 (4.7)
*DRB1*04:05*	1591 (5)	1132 (4.5)	304 (4.3)	420 (2.9)
*DQB1*04:01*	1243 (3.9)	835 (3.3)	230 (3.3)	317 (2.2)
*DQB1*06:09*	572 (1.8)	368 (1.4)	129 (1.8)	201 (1.4)
European descent				
*B*55:01*	7144 (1.8)	3065 (2)	2892 (1.9)	1960 (2)
*B*15:01*	22465 (5.8)	9706 (6.2)	9047 (5.8)	6017 (6.2)
*C*03:03*	20308 (5.2)	8568 (5.5)	8172 (5.2)	5640 (5.8)
*DRB1*01:03*	5238 (1.3)	1321 (0.8)	2138 (1.4)	903 (0.9)
*DRB1*04:03*	3534 (0.9)	1652 (1.1)	1355 (0.9)	1046 (1.1)
*DRB1*07:01*	53101 (13.7)	20634 (13.2)	21114 (13.6)	12725 (13.2)
*DQB1*06:03*	23942 (6.2)	9228 (5.9)	9571 (6.1)	6176 (6.4)
Hispanic or Latino				
*B*35:17*	763 (1.3)	651 (1.7)	108 (1.4)	214 (1.9)
*C*04:01*	9056 (15.5)	6554 (16.9)	1258 (15.9)	1963 (17.2)
*C*07:01*	6329 (10.8)	3835 (9.9)	879 (11.1)	1053 (9.2)
*DRB1*01:03*	565 (1)	182 (0.5)	66 (0.8)	60 (0.5)
*DRB1*04:03*	950 (1.6)	787 (2)	137 (1.7)	263 (2.3)
*DRB1*07:01*	6662 (11.4)	4057 (10.5)	869 (11)	1090 (9.5)
*DRB1*04:07*	3562 (6.1)	2668 (6.9)	518 (6.5)	893 (7.8)
*DQB1*03:02*	10266 (17.5)	7529 (19.4)	1465 (18.5)	2399 (21)
*DQB1*02:01*	10578 (18.1)	6695 (17.3)	1333 (16.8)	1803 (15.8)

Table S3. Significant associated *HLA* alleles in homozygosis

***HLA* Allele**	**NMDP**		
	**OR**	**$CI_{97.5\%}$**	**p-value**
*DRB1*01:03/01:03*			
European descent	0.259	0.076 – 0.661	**1.18E-02**
*DRB1*04:03/04:03*			
Asian or Pacific Islander	1.778	1.041 – 3.079	**3.63E-02**
European descent	3.083	1.642 – 5.874	**4.85E-04**
Hispanic or Latino	0.643	0.192 – 1.875	0.44
*DRB1*07:01/07:01*			
Black or African American	0.764	0.528 – 1.086	0.143
Asian or Pacific Islander	0.864	0.687 – 1.082	0.206
European descent	0.903	0.844 – 0.964	**2.58E-03**
Hispanic or Latino	0.818	0.684 – 0.975	**2.67E-02**

Logistic regression; OR: odds ratio; CI: confidence interval. Bold: significant values (α = 5.0E-02). There was not enough homozygous count for *HLA-DRB1*01:03* for population subgroups AFA, API and HIS, and for *HLA-DRB1*04:03* AFA population subgroup.

Table S4. *HLA-DRB1* amino acid association analysis with CMV serostatus

Black or African American					Asian or Pacific Islander					European descent					Hispanic or Latino				
Position	Residue	OR	CI$_{(95\%)}$	p-value	Position	Residue	OR	CI$_{(95\%)}$	p-value	Position	Residue	OR	CI$_{(95\%)}$	p-value	Position	Residue	OR	CI$_{(95\%)}$	p-value
Pos.-25	K	0.9	0.86 - 0.94	6.71E-06	Pos.-25	K	0.95	0.92 - 0.99	4.52E-03	Pos.-24	F	1.06	1.05 - 1.08	1.56E-14	Pos.-24	F	1.1	1.07 - 1.14	1.59E-09
Pos.-25	R	1.11	1.06 - 1.17	6.71E-06	Pos.-25	R	1.05	1.01 - 1.08	4.52E-03	Pos.-24	L	0.94	0.93 - 0.96	1.56E-14	Pos.-24	L	0.91	0.88 - 0.94	1.59E-09
Pos.-17	A	1.13	1.07 - 1.2	5.48E-06	Pos.-16	A	0.95	0.92 - 0.99	4.52E-03	Pos.-17	A	1.03	1.02 - 1.05	7.18E-07	Pos.-17	A	1.05	1.02 - 1.09	1.87E-03
Pos.-17	T	0.88	0.83 - 0.93	5.48E-06	Pos.-16	V	1.05	1.01 - 1.08	4.52E-03	Pos.-17	T	0.97	0.95 - 0.98	7.18E-07	Pos.-17	T	0.95	0.92 - 0.98	1.87E-03
Pos.-16	A	0.9	0.86 - 0.94	6.71E-06	Pos.-1	A	0.94	0.9 - 0.98	5.81E-03	Pos.4	Q	0.96	0.95 - 0.98	1.64E-05	Pos.-1	A	1.08	1.03 - 1.13	9.19E-04
Pos.-16	V	1.11	1.06 - 1.17	6.71E-06	Pos.-1	S	1.06	1.02 - 1.11	5.81E-03	Pos.4	R	1.04	1.02 - 1.06	1.64E-05	Pos.-1	S	0.93	0.88 - 0.97	9.19E-04
Pos.-1	A	1.14	1.06 - 1.21	1.37E-04	Pos.4	Q	0.94	0.9 - 0.98	3.19E-03	Pos.13	F	0.97	0.95 - 0.98	2.25E-04	Pos.4	Q	0.93	0.89 - 0.96	1.18E-04
Pos.-1	S	0.88	0.82 - 0.94	1.37E-04	Pos.4	R	1.07	1.02 - 1.12	3.19E-03	Pos.13	H	1.06	1.05 - 1.08	1.67E-14	Pos.4	R	1.08	1.04 - 1.12	1.18E-04
Pos.9	E	1.12	1.07 - 1.18	2.43E-06	Pos.9	E	1.03	1 - 1.07	4.92E-02	Pos.13	R	0.98	0.96 - 1	1.55E-02	Pos.9	E	1.08	1.05 - 1.11	9.72E-08
Pos.9	W	0.88	0.84 - 0.93	5.79E-07	Pos.10	Q	0.95	0.92 - 0.99	4.82E-03	Pos.13	S	1.01	1 - 1.02	4.40E-02	Pos.9	W	0.92	0.9 - 0.95	1.12E-08
Pos.10	Q	0.9	0.86 - 0.94	7.23E-06	Pos.10	Y	1.06	1.02 - 1.09	1.65E-03	Pos.13	Y	0.96	0.95 - 0.98	2.21E-05	Pos.11	G	0.91	0.87 - 0.95	7.48E-06
Pos.10	Y	1.1	1.05 - 1.16	2.36E-05	Pos.11	G	0.92	0.87 - 0.98	6.19E-03	Pos.14	E	1.04	1.02 - 1.06	2.21E-05	Pos.11	P	0.96	0.92 - 1	3.00E-02
Pos.11	P	0.87	0.82 - 0.93	2.49E-05	Pos.11	L	0.87	0.78 - 0.98	1.58E-02	Pos.14	K	0.96	0.95 - 0.98	2.21E-05	Pos.11	V	1.1	1.06 - 1.13	3.04E-09
Pos.11	S	1.1	1.05 - 1.16	2.36E-05	Pos.11	S	1.06	1.02 - 1.09	1.65E-03	Pos.25	Q	0.96	0.95 - 0.98	2.20E-05	Pos.14	E	1.1	1.05 - 1.15	7.48E-06
Pos.12	K	0.91	0.86 - 0.95	2.36E-05	Pos.12	K	0.95	0.92 - 0.98	1.65E-03	Pos.25	R	1.04	1.02 - 1.06	2.20E-05	Pos.14	K	0.91	0.87 - 0.95	7.48E-06
Pos.12	T	1.1	1.05 - 1.16	2.36E-05	Pos.12	T	1.06	1.02 - 1.09	1.65E-03	Pos.26	L	0.97	0.95 - 0.99	4.71E-04	Pos.25	Q	0.91	0.87 - 0.95	7.48E-06
Pos.13	R	0.87	0.82 - 0.93	2.49E-05	Pos.13	F	0.94	0.9 - 0.99	1.63E-02	Pos.26	Y	1.02	1 - 1.04	2.87E-02	Pos.25	R	1.1	1.05 - 1.15	7.48E-06
Pos.13	S	1.09	1.04 - 1.14	3.55E-04	Pos.13	S	1.06	1.02 - 1.1	4.33E-03	Pos.28	D	1.04	1.03 - 1.05	4.79E-09	Pos.26	L	0.96	0.92 - 1	4.89E-02
Pos.26	Y	1.15	1.06 - 1.24	3.67E-04	Pos.13	Y	0.92	0.87 - 0.98	6.19E-03	Pos.28	E	0.96	0.95 - 0.97	6.96E-09	Pos.28	D	1.04	1.01 - 1.07	3.86E-03
Pos.30	H	0.93	0.87 - 0.99	1.53E-02	Pos.14	E	1.08	1.02 - 1.15	6.19E-03	Pos.30	C	0.96	0.94 - 0.98	2.84E-05	Pos.28	E	0.95	0.93 - 0.98	1.09E-03
Pos.30	Y	1.08	1.03 - 1.13	2.25E-03	Pos.14	K	0.92	0.87 - 0.98	6.19E-03	Pos.30	L	0.96	0.95 - 0.98	2.21E-05	Pos.30	L	0.91	0.87 - 0.95	7.48E-06
Pos.32	H	1.07	1.02 - 1.12	5.27E-03	Pos.25	Q	0.92	0.87 - 0.98	6.19E-03	Pos.30	Y	1.04	1.03 - 1.05	5.20E-09	Pos.30	Y	1.06	1.03 - 1.09	8.02E-05
Pos.32	Y	0.93	0.89 - 0.98	5.27E-03	Pos.25	R	1.08	1.02 - 1.15	6.19E-03	Pos.31	F	1.03	1.02 - 1.05	2.25E-04	Pos.33	H	1.1	1.07 - 1.14	1.79E-09
Pos.37	N	1.08	1.03 - 1.14	2.34E-03	Pos.30	C	0.87	0.78 - 0.98	1.58E-02	Pos.31	I	0.96	0.94 - 0.98	2.63E-05	Pos.33	N	0.91	0.88 - 0.94	1.79E-09
Pos.37	S	0.88	0.84 - 0.93	6.56E-06	Pos.30	H	1.06	1.01 - 1.12	2.45E-02	Pos.32	H	1.01	1 - 1.03	4.40E-02	Pos.37	F	0.9	0.86 - 0.93	4.21E-08
Pos.37	Y	1.06	1.01 - 1.11	2.27E-02	Pos.30	L	0.92	0.87 - 0.98	6.19E-03	Pos.32	Y	0.99	0.97 - 1	4.40E-02	Pos.37	S	0.95	0.92 - 0.98	9.10E-04
Pos.67	I	0.93	0.89 - 0.97	1.94E-03	Pos.31	F	1.06	1.01 - 1.11	1.63E-02	Pos.33	H	1.06	1.05 - 1.08	1.67E-14	Pos.37	Y	1.09	1.07 - 1.12	9.60E-12
Pos.67	L	1.06	1.01 - 1.12	1.27E-02	Pos.31	I	0.94	0.89 - 0.99	2.50E-02	Pos.33	N	0.94	0.93 - 0.96	1.67E-14	Pos.47	F	0.94	0.92 - 0.97	3.48E-05
Pos.71	A	0.88	0.82 - 0.94	1.41E-04	Pos.32	H	1.04	1.01 - 1.08	2.04E-02	Pos.37	F	0.97	0.96 - 0.99	6.58E-04	Pos.47	Y	1.06	1.03 - 1.09	3.48E-05
Pos.71	K	1.12	1.06 - 1.19	7.60E-05	Pos.32	Y	0.96	0.93 - 0.99	2.04E-02	Pos.37	S	0.97	0.95 - 0.98	7.76E-07	Pos.57	A	0.87	0.8 - 0.95	2.62E-03
Pos.73	A	0.95	0.9 - 1	4.16E-02	Pos.37	L	1.06	1.01 - 1.12	2.28E-02	Pos.37	Y	1.04	1.03 - 1.05	3.46E-10	Pos.57	D	1.04	1.01 - 1.07	1.76E-02
Pos.73	G	1.06	1 - 1.12	4.16E-02	Pos.47	F	1.1	1.07 - 1.14	8.61E-09	Pos.57	D	1.03	1.01 - 1.04	2.84E-04	Pos.57	S	1.08	1.03 - 1.13	2.68E-03
Pos.74	A	0.93	0.89 - 0.97	2.40E-03	Pos.47	Y	0.91	0.88 - 0.94	8.61E-09	Pos.57	V	0.97	0.95 - 0.98	3.48E-05	Pos.57	V	0.93	0.89 - 0.96	1.06E-04
Pos.74	R	1.14	1.07 - 1.22	1.06E-04	Pos.50	A	1.33	1.04 - 1.7	1.89E-02	Pos.71	E	0.94	0.93 - 0.96	1.32E-11	Pos.60	H	0.87	0.8 - 0.96	2.86E-03
Pos.77	N	1.14	1.07 - 1.22	1.06E-04	Pos.50	V	0.75	0.59 - 0.96	1.89E-02	Pos.71	K	1.06	1.05 - 1.08	<2.22E-16	Pos.60	S	0.93	0.89 - 0.96	7.21E-05
Pos.77	T	0.88	0.82 - 0.94	1.06E-04	Pos.58	A	0.86	0.8 - 0.92	1.13E-05	Pos.85	A	0.96	0.92 - 0.99	9.80E-03	Pos.60	Y	1.09	1.06 - 1.13	8.33E-07
Pos.96	H	1.1	1.05 - 1.16	1.44E-04	Pos.58	E	1.16	1.09 - 1.24	1.13E-05	Pos.85	V	1.05	1.01 - 1.08	9.80E-03	Pos.67	I	0.9	0.87 - 0.92	8.02E-15
Pos.96	Q	0.89	0.84 - 0.95	1.90E-04	Pos.60	H	1.08	1 - 1.16	4.24E-02	Pos.96	E	0.96	0.94 - 0.98	2.96E-05	Pos.67	L	1.08	1.06 - 1.11	7.01E-10
Pos.133	L	0.87	0.82 - 0.93	2.43E-05	Pos.67	F	1.07	1.03 - 1.11	6.86E-04	Pos.96	Q	0.98	0.97 - 1	4.36E-02	Pos.70	D	0.95	0.93 - 0.98	1.91E-04
Pos.133	R	1.14	1.07 - 1.22	2.43E-05	Pos.71	A	1.06	1.02 - 1.1	6.48E-03	Pos.96	Y	1.06	1.05 - 1.08	1.81E-14	Pos.70	Q	1.06	1.03 - 1.09	1.97E-05
Pos.140	A	0.89	0.85 - 0.94	2.31E-06	Pos.71	R	0.96	0.93 - 1	2.55E-02	Pos.98	E	1.02	1.01 - 1.03	4.36E-03	Pos.71	A	0.92	0.88 - 0.97	7.34E-04

Pos.140	T	1.12	1.07 - 1.18	2.31E-06	Pos.74	L	0.89	0.82 - 0.96	1.31E-03	Pos.98	K	0.98	0.97 - 0.99	4.36E-03	Pos.71	E	0.92	0.89 - 0.96	6.08E-05
Pos.142	M	0.87	0.82 - 0.93	2.43E-05	Pos.74	Q	0.92	0.87 - 0.98	6.19E-03	Pos.104	A	1.02	1.01 - 1.03	4.36E-03	Pos.71	R	1.08	1.05 - 1.11	9.14E-09
Pos.142	V	1.14	1.07 - 1.22	2.43E-05	Pos.78	V	0.94	0.9 - 0.98	3.12E-03	Pos.104	S	0.98	0.97 - 0.99	4.36E-03	Pos.73	A	1.06	1.03 - 1.1	2.72E-04
Pos.149	H	1.1	1.05 - 1.16	2.23E-05	Pos.78	Y	1.07	1.02 - 1.12	3.12E-03	Pos.120	N	1.06	1.05 - 1.08	4.68E-15	Pos.73	G	0.94	0.91 - 0.97	2.72E-04
Pos.149	Q	0.91	0.86 - 0.95	2.23E-05	Pos.85	A	1.05	1 - 1.11	4.87E-02	Pos.120	S	0.94	0.93 - 0.95	4.68E-15	Pos.74	A	0.97	0.94 - 0.99	7.44E-03
Pos.233	R	1.08	1.03 - 1.13	7.70E-04	Pos.85	V	0.95	0.9 - 1	4.87E-02	Pos.133	L	0.98	0.96 - 1	1.56E-02	Pos.74	E	1.11	1.07 - 1.16	1.38E-08
Pos.233	T	0.92	0.88 - 0.97	7.70E-04	Pos.86	G	0.94	0.91 - 0.98	6.88E-04	Pos.133	R	1.02	1 - 1.04	1.56E-02	Pos.74	L	1.07	1.02 - 1.11	4.71E-03
					Pos.86	V	1.06	1.02 - 1.1	6.88E-04	Pos.140	A	0.96	0.94 - 0.97	5.46E-14	Pos.74	Q	0.91	0.87 - 0.95	7.20E-06
					Pos.96	E	0.87	0.78 - 0.98	1.60E-02	Pos.140	T	1.05	1.03 - 1.06	5.46E-14	Pos.78	V	0.93	0.89 - 0.96	1.32E-04
					Pos.98	E	0.94	0.91 - 0.97	5.92E-04	Pos.142	M	0.98	0.96 - 1	1.56E-02	Pos.78	Y	1.08	1.04 - 1.12	1.32E-04
					Pos.98	K	1.07	1.03 - 1.1	5.92E-04	Pos.142	V	1.02	1 - 1.04	1.56E-02	Pos.96	H	0.97	0.94 - 0.99	1.28E-02
					Pos.104	A	0.94	0.91 - 0.97	5.92E-04	Pos.180	L	1.06	1.05 - 1.08	1.81E-14	Pos.96	Q	0.96	0.93 - 1	4.71E-02
					Pos.104	S	1.07	1.03 - 1.1	5.92E-04	Pos.180	V	0.94	0.93 - 0.96	1.81E-14	Pos.96	Y	1.1	1.07 - 1.14	1.60E-09
					Pos.149	H	1.06	1.02 - 1.09	1.62E-03	Pos.181	M	0.97	0.95 - 0.98	1.22E-04	Pos.98	E	1.04	1.01 - 1.06	1.08E-02
					Pos.149	Q	0.95	0.92 - 0.98	1.62E-03	Pos.181	T	1.03	1.02 - 1.05	1.22E-04	Pos.98	K	0.97	0.94 - 0.99	1.08E-02
					Pos.181	M	0.94	0.9 - 0.98	2.03E-03						Pos.104	A	1.04	1.01 - 1.06	1.08E-02
					Pos.181	T	1.07	1.02 - 1.11	2.03E-03						Pos.104	S	0.97	0.94 - 0.99	1.08E-02
					Pos.189	R	1.13	1.05 - 1.22	1.01E-03						Pos.120	N	1.1	1.06 - 1.13	2.63E-09
					Pos.189	S	0.88	0.82 - 0.95	1.01E-03						Pos.120	S	0.91	0.88 - 0.94	2.63E-09
					Pos.233	R	1.09	1.05 - 1.12	2.08E-06						Pos.133	L	0.96	0.92 - 1	3.02E-02
					Pos.233	T	0.92	0.89 - 0.95	2.08E-06						Pos.133	R	1.05	1 - 1.09	3.02E-02
															Pos.140	A	0.93	0.9 - 0.95	8.26E-08
															Pos.140	T	1.08	1.05 - 1.11	8.26E-08
															Pos.142	M	0.96	0.92 - 1	3.02E-02
															Pos.142	V	1.05	1 - 1.09	3.02E-02
															Pos.180	L	1.1	1.07 - 1.14	1.60E-09
															Pos.180	V	0.91	0.88 - 0.94	1.60E-09
															Pos.181	M	0.93	0.9 - 0.97	3.17E-04
															Pos.181	T	1.07	1.03 - 1.11	3.17E-04
															Pos.189	R	0.94	0.9 - 0.98	4.32E-03
															Pos.189	S	1.07	1.02 - 1.12	4.32E-03

Highlighted in red: residues that lies in the binding pocket P4 (residues 71 and 74) and P7 (residues 67 and 71).

Table S5. Peptide binding prediction among the most immunogenic CMV proteins and *HLA* class II associated with CMV in this study.

CMV protein	Peptide	*HLA* Allele	Score_EL	%Rank_EL	Bind Level	*HLA* Allele	Score_EL	%Rank_EL	Bind Level
pp65	DTPVLPHETRLLQTG	*DRB1*01:03*	0.545284	1.41	1 SB	*DRB1*01:01*	0.07773	12.44	52
pp65	ASVLGPISGHVLKAV	*DRB1*01:03*	0.528303	1.51	2 SB	*DRB1*01:01*	0.488896	2.78	3 WB
pp65	PKNMIIKPGKISHIM	*DRB1*01:03*	0.504027	1.68	3 SB	*DRB1*01:01*	0.039001	18.18	81
pp65	CPKNMIIKPGKISHI	*DRB1*01:03*	0.481895	1.87	4 SB	*DRB1*01:01*	0.033076	19.79	94
pp65	RGRLKAESTVAPEED	*DRB1*04:01*	0.828983	0.43	1 SB	*DRB1*04:03*	0.743531	0.77	3 SB
pp65	VSQYTPDSTPCHRGD	*DRB1*04:01*	0.820568	0.46	2 SB	*DRB1*04:03*	0.231153	9.44	52 WB
pp65	TRGRLKAESTVAPEE	*DRB1*04:01*	0.750475	0.74	3 SB	*DRB1*04:03*	0.663426	1.38	6 SB
pp65	LVSQYTPDSTPCHRG	*DRB1*04:01*	0.72639	0.87	4 SB	*DRB1*04:03*	0.15702	13.47	73
pp65	GGAMAGASTSAGRKR	*DRB1*04:01*	0.671587	1.19	5 SB	*DRB1*04:03*	0.836002	0.3	1 SB
pp65	SQYTPDSTPCHRGDN	*DRB1*04:01*	0.574326	1.89	6 SB	*DRB1*04:03*	0.085767	20.69	108
pp65	GGGAMAGASTSAGRK	*DRB1*04:01*	0.56122	1.99	7 SB	*DRB1*04:03*	0.78398	0.55	2 SB
IE1	LSSVIVAENSDQEES	*DRB1*04:01*	0.757261	0.71	1 SB	*DRB1*04:03*	0.873976	0.16	1 SB
IE1	SSVIVAENSDQEESE	*DRB1*04:01*	0.743833	0.78	2 SB	*DRB1*04:03*	0.845421	0.26	2 SB
IE1	PLSSVIVAENSDQEE	*DRB1*04:01*	0.608957	1.6	3 SB	*DRB1*04:03*	0.816709	0.39	3 SB
IE2	GIQIIYTRNHEVKSE	*DRB1*01:03*	0.859046	0.17	1 SB	*DRB1*01:01*	0.685628	1.47	5 SB
IE2	STPFLMEHTMPVTHP	*DRB1*01:03*	0.853592	0.19	2 SB	*DRB1*01:01*	0.95804	0.13	1 SB
IE2	KGIQIIYTRNHEVKS	*DRB1*01:03*	0.801849	0.32	3 SB	*DRB1*01:01*	0.561059	2.22	8 WB
IE2	LSTPFLMEHTMPVTH	*DRB1*01:03*	0.755569	0.46	4 SB	*DRB1*01:01*	0.897276	0.41	2 SB
IE2	IQIIYTRNHEVKSEV	*DRB1*01:03*	0.691367	0.69	5 SB	*DRB1*01:01*	0.357264	4.06	17 WB
IE2	DYRNMIIHAATPVDL	*DRB1*01:03*	0.683552	0.72	6 SB	*DRB1*01:01*	0.263396	5.36	28 WB
IE2	SDYRNMIIHAATPVD	*DRB1*01:03*	0.666465	0.79	7 SB	*DRB1*01:01*	0.304748	4.72	21WB
IE2	YRNMIIHAATPVDLL	*DRB1*01:03*	0.658343	0.82	8 SB	*DRB1*01:01*	0.272926	5.2	25 WB
IE2	TPFLMEHTMPVTHPP	*DRB1*01:03*	0.654846	0.83	9 SB	*DRB1*01:01*	0.798646	0.88	3 SB
IE2	NKGIQIIYTRNHEVK	*DRB1*01:03*	0.650678	0.85	10 SB	*DRB1*01:01*	0.333038	4.35	18 WB
IE2	SSDYRNMIIHAATPV	*DRB1*01:03*	0.617826	0.99	11 SB	*DRB1*01:01*	0.534337	2.41	10 WB
IE2	RSSDYRNMIIHAATP	*DRB1*01:03*	0.554302	1.35	12 SB	*DRB1*01:01*	0.507004	2.63	11 WB
IE2	ALSTPFLMEHTMPVT	*DRB1*01:03*	0.53811	1.45	13 SB	*DRB1*01:01*	0.715405	1.3	4 SB
IE2	RNMIIHAATPVDLLG	*DRB1*01:03*	0.494868	1.76	14 SB	*DRB1*01:01*	0.3818	3.78	14 WB
IE2	TRPFKVIIKPPVPPA	*DRB1*01:03*	0.492674	1.78	15 SB	*DRB1*01:01*	0.283	5.03	23 WB
IE2	LGDILAQAVNHAGID	*DRB1*04:01*	0.847132	0.36	1 SB	*DRB1*04:03*	0.882893	0.13	1 SB
IE2	STPFLMEHTMPVTHP	*DRB1*04:01*	0.84588	0.36	2 SB	*DRB1*04:03*	0.523165	2.76	26 WB
IE2	GDILAQAVNHAGIDS	*DRB1*04:01*	0.776221	0.63	3 SB	*DRB1*04:03*	0.840081	0.28	6 SB
IE2	ELGDILAQAVNHAGI	*DRB1*04:01*	0.755582	0.72	4 SB	*DRB1*04:03*	0.813539	0.41	7 SB
IE2	LSTPFLMEHTMPVTH	*DRB1*04:01*	0.740686	0.79	5 SB	*DRB1*04:03*	0.406763	4.5	45 WB
IE2	SSDYRNMIIHAATPV	*DRB1*04:01*	0.715173	0.93	6 SB	*DRB1*04:03*	0.467666	3.52	32 WB
IE2	RSSDYRNMIIHAATP	*DRB1*04:01*	0.709733	0.97	7 SB	*DRB1*04:03*	0.466951	3.53	33 WB
IE2	KQVMVRIFSTNQGGF	*DRB1*04:01*	0.68533	1.11	8 SB	*DRB1*04:03*	0.875435	0.16	2 SB
IE2	QVMVRIFSTNQGGFM	*DRB1*04:01*	0.655529	1.28	9 SB	*DRB1*04:03*	0.85674	0.22	3 SB
IE2	AELGDILAQAVNHAG	*DRB1*04:01*	0.65254	1.3	10 SB	*DRB1*04:03*	0.72912	0.85	13 SB
IE2	NKGIQIIYTRNHEVK	*DRB1*04:01*	0.613346	1.57	11 SB	*DRB1*04:03*	0.797372	0.49	8 SB
IE2	PKQVMVRIFSTNQGG	*DRB1*04:01*	0.57969	1.85	12 SB	*DRB1*04:03*	0.849283	0.25	4 SB
IE2	TPFLMEHTMPVTHPP	*DRB1*04:01*	0.561179	1.99	13 SB	*DRB1*04:03*	0.263329	8.17	61 WB

EL: Eluted Ligand mass spectrometry; SB: strong binder; WB: weak binder; Threshold for strong binder (% Rank): 2; Threshold for weak binder (% Rank): 10

Table S6. Charge and volume of the polymorphic residues in the HVR3 for *HLA-DRB1*01:01*, *HLA-DRB1*01:03*, *HLA-DRB1*04:01* and *HLA-DRB1*04:03*

	*HLA-DRB1*01:01*	*HLA-DRB1*01:03*	*HLA-DRB1*04:01*	*HLA-DRB1*04:03*
**Residue 67**				
Amino acid	L - leucine	I - isoleucine	L - leucine	L - leucine
Charge	uncharged	uncharged	uncharged	uncharged
Volume Å3	large 166.7	large 166.7	large 166.7	large 166.7
**Residue 70**				
Amino acid	Q - glutamine	D - aspartic acid	Q - glutamine	Q - glutamine
Charge	uncharged	negative	uncharged	uncharged
Volume Å3	medium 143.8	small 111.1	medium 143.8	medium 143.8
**Residue 71**				
Amino acid	R - arginine	E - glutamic acid	K - lysine	R - arginine
Charge	positive	negative	positive	positive
Volume Å3	large 173.4	medium 138.4	large 168.6	large 173.4
**Residue 74**				
Amino acid	A - alanine	A - alanine	A - alanine	E - glutamic acid
Charge	uncharged	uncharged	uncharged	negative
Volume Å3	very small 88.6	very small 88.6	very small 88.6	medium 138.4
**Residue 86**				
Amino acid	G - glycine	G - glycine	G - glycine	V - valine
Charge	uncharged	uncharged	uncharged	uncharged
Volume Å3	very small 60.1	very small 60.1	very small 60.1	medium 140

Blue: denotes a change in the amino acid residue;
Red: denotes changes in charge and volume.
