# Supplementary material for: Human leukocyte antigen variation is associated with cytomegalovirus serostatus in healthy individuals
Source: Am J Hum Genet. 2025 Mar 5;112(4):913–26. doi: 10.1016/j.ajhg.2025.02.007 (PMC12081270; doi:10.1016/j.ajhg.2025.02.007)
Supplement: Document S2. Article plus supplemental information [file mmc2.pdf]

Human leukocyte antigen variation is associated with cytomegalovirus serostatus in healthy individuals

Authors

Juliano A. Boquett, Jürgen Sauter, Alexander H. Schmidt, Martin Maiers, Jill A. Hollenbach

Correspondence

jill.hollenbach@ucsf.edu

Cytomegalovirus (CMV) is a common virus worldwide; while infection is usually benign, it can cause severe illness in newborns and immunocompromised individuals. We evaluated variation in immune genes in risk for CMV in more than 518,000 individuals to better understand how the immune response to the virus differs between individuals.

Boquett et al., 2025, The American Journal of Human Genetics *112*, 913–926
April 3, 2025

© 2025 The Author(s). Published by Elsevier Inc. on behalf of American Society of Human Genetics.
https://doi.org/10.1016/j.ajhg.2025.02.007

Human leukocyte antigen variation is associated with cytomegalovirus serostatus in healthy individuals

Juliano A. Boquett,[1] Jürgen Sauter,[2] Alexander H. Schmidt,[2] Martin Maiers,[3] and Jill A. Hollenbach[1,4,*]

Summary

Cytomegalovirus (CMV) is a common β-herpes virus worldwide with an estimated seroprevalence among the general population of 83%. Primary infection is usually benign; however, CMV can cause severe morbidity in newborns in whom it is acquired congenitally, as well as immunocompromised individuals. Understanding the role of immunogenetic variation in risk for CMV infection can provide insight into the immune control of this ubiquitous pathogen. Here, we evaluated the association of human leukocyte antigen (*HLA*) genetic variation with CMV seropositivity in more than 518,000 individuals from two independent cohorts. We found three *HLA* class II alleles (*HLA-DRB1*04:03* with risk; *HLA-DRB1*01:03* and *HLA-DRB1*07:01* with protection) to be significantly associated with CMV serostatus across both cohorts and in multiple population subgroups. Interestingly, *HLA-DRB1*04:03* and *HLA-DRB1*01:03*, the alleles with the strongest observed effect, are relatively rare, while common homologous alleles show no association with CMV. We show that these differences are mediated by changes in charge and volume to two key pockets in the peptide-binding groove of the HLA molecule, providing a structural basis for the observed association. Our results provide population-scale evidence for the role of HLA in mediating infection with this ubiquitous human virus and a framework for understanding immunological conditions necessary for efficient viral control.

Introduction

Cytomegalovirus (CMV) is an ancient β-herpes virus (type 5) that is ubiquitous in all human populations. Infection with CMV is common across the globe, with an estimated seroprevalence among the general population of 83%.[1] However, the estimated CMV seroprevalence varies among WHO (World Health Organization) regions: 66% in Europe, 75% in the Americas region, 86% in Southeast Asia, 88% in Africa and Western Pacific regions, and 90% in the Eastern Mediterranean.[1] Active CMV infection can occur in three different ways: (1) primary infection (including congenital infection), (2) reactivation of latent CMV, and (3) reinfection with a new strain of CMV.[2] Generally, CMV seroprevalence is higher among women, older age groups, those from lower socioeconomic status (SES), and those in developing countries.[3] CMV infection is typically asymptomatic in healthy individuals, establishing a lifelong latent infection. However, CMV can cause long-term impacts on the health status of immunocompromised individuals, transplant recipients, those with late-stage human immunodeficiency virus (HIV) infection, and the elderly and congenitally infected neonates.[4,5]

CMV infection and its adverse sequelae are closely tied to social factors. Lower SES is associated with increased mortality from CMV among adults in the US, independent of age.[6] Globally, CMV seroprevalence is correlated with ancestry, SES, education level,[7] and increased mortality in older age.[6] Furthermore, congenital CMV infection is associated with lower SES and young maternal age, is more prevalent among HIV-seropositive patients, and is significantly more common in low- and middle-income countries in comparison to high-income countries.[8]

CMV primary infection initiates with viral replication in the mucosal epithelium, disseminating to monocytic cells of myeloid lineage, where it establishes latent infection.[9] After the establishment of primary infection, virus particles are processed and presented by antigen-presenting cells, which elicits the antigen-specific immune response. The virus efficiently adapts to the human immune system and can evade it using different pathways, e.g., by downregulating human leukocyte antigen (*HLA*) class I expression on the cell surface,[10] causing a persistent asymptomatic infection.

HLA genes are notably the most polymorphic of the human genome.[11] This remarkable variation leads to distinctive peptide-binding repertoires, whereby individuals with different *HLA* genotypes may exhibit different abilities in antigen presentation and differentially elicit immune responses, underlying *HLA* associations with human disease, including viral infections.[12] For example, *HLA* class I variants have been associated with the slow progression of HIV infection,[13] while *HLA* class II variants are associated with both viral persistence and clearance for hepatitis B (HBV) and C (HCV) viruses.[14] Our work and that of others has also demonstrated a role for *HLA* in COVID-19 disease outcomes.[15,16] Notably, *HLA* associations with infection by herpesvirus other than CMV have been described.[17] While the role of *HLA* variation has been examined in the context of CMV, studies have largely focused on immunosuppressed patients in the transplant setting.[18–21]

[1]Department of Neurology, University of California, San Francisco, San Francisco, CA, USA; [2]DKMS Group, Tübingen, Germany; [3]CIBMTR (Center for International Blood and Marrow Transplant Research), NMDP, Minneapolis, MN, USA; [4]Department of Epidemiology and Biostatistics, University of California, San Francisco, San Francisco, CA, USA
*Correspondence: jill.hollenbach@ucsf.edu
https://doi.org/10.1016/j.ajhg.2025.02.007.
© 2025 The Author(s). Published by Elsevier Inc. on behalf of American Society of Human Genetics.
This is an open access article under the CC BY license (http://creativecommons.org/licenses/by/4.0/).

In addition to their role in infection immunity, *HLA* genes are the primary determinant for tolerance or rejection in solid organ and hematopoietic stem cell transplant (HSCT). *HLA*-matched transplantation between a patient and donor presents the best outcome due to the prevention of immune rejection of foreign tissue and the facilitation of immune reconstitution.[22] The NMDP (formerly known as the National Marrow Donor Program) is a large and diverse donor registry with over 9 million potential HSCT donors registered in the US. DKMS has registered over 12 million HSCT registry members in at least seven countries, including 1.1 million potential bone marrow donors registered in the US. Registries store demographic data such as gender, age, race/ancestry, zip code of residence, and high-resolution *HLA* genotyping for registered donors. Because CMV reactivation after allogeneic HSCT (allo-HSCT) has been associated with increased transplant-related mortality and end-organ diseases such as pneumonia, hepatitis, and colitis,[23] and CMV infection is associated with increased risk for graft-versus-host disease (GVHD),[24,25] CMV-seropositive HSCT recipients and CMV-seronegative recipients with a CMV-seropositive donor are considered at risk for CMV disease after HSCT.[26] Thus, these registries also routinely screen potential donors for CMV serostatus, which can be used as a proxy for CMV infection.

Because of their central role in the immune response to pathogens, we hypothesized that variation in *HLA* genes may impact infection with CMV in healthy individuals. Here, we examine *HLA* variation with respect to serostatus for CMV using high-resolution *HLA* genotyping data from more than 518,000 individuals registered as donors with the NMDP and DKMS stem cell donor registries. Our results show that *HLA* alleles associated with CMV serostatus have effects replicating across both registries and multiple population subgroups, revealing, for the first time, a role for *HLA* variation in susceptibility to CMV infection.

Methods

NMDP (discovery) cohort

The discovery cohort includes anonymized data from 366,481 individuals who volunteered as potential hematopoietic stem cell donors at NMDP from January 2018 to February 2023, covering all 50 states in the US. The dataset includes information such as gender, age, self-assigned race (as a proxy for ancestry), NDI (neighborhood deprivation index), CMV serostatus, and high-resolution *HLA* genotyping for all classical class I (*HLA-A* [MIM: 142800], *HLA-B* [MIM: 142830], and *HLA-C* [MIM: 142840]) and class II (*HLA-DRB1* [MIM: 142857] and *HLA-DQB1* [MIM: 604305]) loci. Genotyping was performed through next-generation sequencing (NGS), covering the antigen recognition domain (ARD) exons. The PacBio sequencing platform was used to perform full gene-phased sequencing. Any genotyping ambiguities are addressed through a bioinformatic method aimed at resolving these genotypes.[27] Four major population subgroups according to self-reported ancestry were included in the study as follows: Black or African American (AFA), Asian or Pacific Islander (API), European descent (EUR), and Hispanic or Latino (HIS). The CMV-seropositive group comprised 115,739 individuals, and the CMV-seronegative group comprised 250,742 individuals, distributed in the four different population groups. Detailed sample sizes are presented in Table 1. The data were deidentified, and their use for this study was approved by the NMDP IRB (institutional review board).

DKMS (replication) cohort

The replication cohort includes a total of 152,335 individuals registered at DKMS living in the US who volunteered as potential hematopoietic stem cell donors from February 2006 to October 2023. The dataset includes the same variables as the NMDP dataset (deidentified records of gender, age, self-assigned ancestry, NDI, CMV serostatus, and high-resolution *HLA* genotyping for *HLA-A*, *HLA-B*, *HLA-C*, *HLA-DRB1*, and *HLA-DQB1*).[28,29] Genotyping ambiguities are treated as described in the NDMP cohort.[27] Again, the four major population groups were included in the study (AFA, API, EUR, and HIS). The CMV-seropositive group comprised 64,610 individuals, and the CMV-seronegative group comprised 87,725 individuals, distributed in the four different population groups. Detailed sample sizes are presented in Table 1.

Sociodemographic characteristics

The Fisher's exact test was used for sex comparisons, the Wilcoxon rank-sum test was applied for comparisons of age and NDI means, and the chi-squared test was performed to compare NDI quintiles between groups on SPSS software (v.20.0). We used the NDI as a proxy measure for SES. NDI is a tool created using factor analysis to identify key variables from 13 measures in the dimensions of SES as follows: wealth and income, education, occupation, and housing conditions.[30,31]

HLA association analysis

We examined the association of five *HLA* loci (*HLA-A*, *HLA-B*, *HLA-C*, *HLA-DRB1*, *HLA-DQB1*) with CMV serostatus (CMV seropositive versus CMV seronegative) in our discovery cohort. *HLA* data included the first two fields of the allele name as described in the *HLA* nomenclature, which defines HLA allotypes. Only alleles with a frequency greater than 1% were included in the analysis (94 alleles for the AFA subgroup and 97, 108, and 85 for API, HIS, and EUR, respectively). *HLA*-association testing was performed through logistic regression by the glm base package on RStudio[32] (v.2024.4.2.764), adjusting for sex, age, and NDI for each population subgroup separately. The corrected p value for the *HLA* allele association applied for the logistic regression in our discovery dataset was calculated considering the total number of alleles with a frequency greater than 1% for *HLA-A* (14), *HLA-B* (23), and *HLA-DRB1* (21) in our largest population subgroup (EUR) ($\alpha = 0.05/58 = 8.62E-04$), which considers the strong linkage disequilibrium between some of the loci tested. We repeated the association analysis for alleles significant at the corrected p value in the discovery cohort only (55 alleles among all the four population subgroups together) in our replication cohort, considering the significance level at $\alpha = 0.05$ after applying the Benjamini-Hochberg (FDR [false discovery rate]) correction.

Trans-population meta-analysis was performed for the alleles that were significantly associated with CMV in both the NMDP and DKMS datasets in at least two different populations using the common effect model with the meta package (v.6.5-0)[33] on RStudio. The meta-analysis was performed using NMDP data.

Table 1. Sociodemographic characteristics

	NMDP			DKMS		
	Case	Control	p value	Case	Control	p value
**Sex, n (%)**						
Female	85,649 (74)	169,924 (67.8)	–	46,837 (72.5)	58,069 (66.2)	–
Male	30,090 (26)	80,818 (32.2)	<0.001[a]	17,773 (27.5)	29,656 (33.8)	<0.001[a]
Age, mean (SD)	30 (7)	27.3 (6.8)	<0.001[b]	33.4 (10.6)	30.8 (10.8)	<0.001[b]
**Subgroup, n (%)**						
AFA	5,602 (4.3)	11,150 (4.1)	–	3,263 (4.4)	2,365 (2.4)	–
API	12,696 (9.8)	15,879 (5.8)	–	7,334 (9.9)	3,508 (3.5)	–
EUR	78,056 (60.1)	194,446 (70.7)	–	48,304 (65.2)	77,888 (78.8)	–
HIS	19,385 (14.9)	29,267 (10.6)	–	5,709 (7.7)	3,964 (4)	–

AFA, Black or African American; API, Asian or Pacific Islander; EUR, European descent; HIS, Hispanic or Latino.
[a]Fisher's exact test.
[b]Wilcoxon rank-sum test.

Volcano plots were made using the ggplot2 package (v.3.4.4)[34] on RStudio.

The analysis of individual *HLA-DRB1* amino acids with CMV serostatus for the four population subgroups was accomplished with the BIGDAWG package (v.3.0.3)[35] on RStudio.

Peptide-binding prediction

Peptide-binding prediction analysis included the *HLA-DRB1* alleles showing the strongest protective and risk effects for CMV (*HLA-DRB1*01:03* and *HLA-DRB1*04:03*, respectively) and their more common related alleles that showed no association with the virus (*HLA-DRB1*01:01* and *HLA-DRB1*04:01*). The prediction analysis was performed for the three most immunogenic CMV proteins (pp65, IE-1, and IE-2)[36] with these four *HLA-DRB1* alleles using NetMHCIIpan 4.1 for HLA class II.[37] The parameters were set as default, where the peptide is identified as a strong binder (SB) if it is found among the top 2% predicted peptides for the eluted ligand likelihood prediction method. CMV protein sequences were retrieved from the following accession numbers: pp65 (GenBank: AAA45994.1), IE-1 (GenBank: UBQ34175.1), and IE-2 (GenBank: CAG7582958.1).

Protein structure modeling prediction

The protein structure modeling prediction included the α (*HLA-DRA*) and β (*HLA-DRB1*) chains and the CMV pp65 peptides with the biggest elution ligand (EL) score difference between *HLA-DRB1*01:01* and *HLA-DRB1*01:03* as well as *HLA-DRB1*04:01* and *HLA-DRB1*04:03*. The *HLA-DRB1*01:01*, *HLA-DRB1*01:03*, *HLA-DRB1*04:01*, and *HLA-DRB1*04:03* alleles were included for β chain modeling prediction, which was performed on AlphaFold 3[38] with the parameters set as default. The electrostatic potential for contacts between α and β chains with the CMV peptide was performed using USCF ChimeraX.[39]

Results

CMV seropositivity is correlated with SES and demographics in large HSCT donor registries

Because of the previously documented association of CMV infection/seropositivity with socioeconomic and demographic factors, we first considered these in our analyses. SES can be measured through the NDI, a tool created using factor analysis to identify key variables from 13 measures of the dimensions of SES.[30,31] The sociodemographic characteristics of the cohorts can be found in Table 1. As expected, age and the number of females were significantly higher in the case group in both our discovery (NMDP, $N = 366{,}481$) and replication (DKMS, $N = 152{,}335$) cohorts. Furthermore, the case group presented a higher frequency in most NDI quintiles than the control group in each of the four population subgroups (AFA, API, EUR, and HIS) tested in both cohorts (Table S1; Figure S1). The AFA subgroup presented higher NDIs in both datasets, while API presented lower NDIs in both cohorts. Thus, we confirmed the importance of these factors in the present cohorts of healthy donors in the US.

Three *HLA* variants show consistent association with risk or protection in CMV infection

We next examined the association of all *HLA* class I and class II alleles with CMV seropositivity through logistic regression controlling for sex, age, and NDI in the four population subgroups separately for both the discovery and replication cohorts. A substantial number of alleles with a frequency > 1% were found to be associated with CMV seropositivity in our discovery (NMDP) cohort that replicated in the DKMS cohort (Tables 2 and S2; Figure 1). Of these, three alleles (*HLA-DRB1*01:03*, *HLA-DRB1*04:03*, and *HLA-DRB1*07:01*) were significantly associated with CMV seropositivity in at least two different population subgroups, and while not reaching statistical significance in other groups, we observed consistent effect sizes across all subgroups considered (Tables 3 and S2). Among those alleles, one was significantly associated with risk (*HLA-DRB1*04:03* in EUR and HIS) and two with protection from CMV infection (*HLA-DRB1*01:03* and *HLA-DRB1*07:01* in EUR and HIS). A trans-population

Table 2. Significant *HLA* alleles associated with CMV in NMDP that replicate in the DKMS cohort in a single population subgroup

	NMDP			DKMS		
HLA allele	OR	$CI_{97.5\%}$	*p* value[a]	OR	$CI_{97.5\%}$	*p* value[b]
**Black or African American**						
*DRB1*15:01*	0.759	(0.646–0.887)	6.28E−04	0.676	(0.532–0.857)	4.95E−03
**Asian or Pacific Islander**						
*A*01:01*	1.196	(1.110–1.289)	2.66E−06	1.239	(1.106–1.388)	9.99E−04
*A*02:07*	0.833	(0.763–0.909)	4.15E−05	0.749	(0.638–0.880)	1.74E−03
*B*40:01*	0.861	(0.804–0.923)	2.65E−05	0.65	(0.572–0.739)	1.34E−09
*DRB1*14:04*	1.202	(1.081–1.337)	6.68E−04	1.378	(1.177–1.619)	4.52E−04
*DRB1*04:05*	0.841	(0.773–0.914)	4.83E−05	0.636	(0.542–0.746)	3.07E−07
*DQB1*04:01*	0.804	(0.731–0.884)	7.11E−06	0.649	(0.541–0.779)	3.12E−05
*DQB1*06:09*	0.791	(0.689–0.907)	8.36E−04	0.721	(0.573–0.909)	1.62E−02
**European descent**						
*B*55:01*	1.087	(1.040–1.135)	2.29E−04	1.099	(1.035–1.166)	7.15E-03
*B*15:01*	1.072	(1.044–1.100)	2.32E−07	1.076	(1.038–1.115)	3.47E-04
*C*03:03*	1.051	(1.023–1.081)	3.94E−04	1.122	(1.082–1.165)	1.71E−08
*DQB1*06:03*	0.953	(0.928–0.978)	3.18E−04	1.052	(1.015–1.089)	1.62E−02
**Hispanic or Latino**						
*B*35:17*	1.312	(1.175–1.465)	1.37E−06	1.369	(1.080–1.744)	2.79E−02
*C*04:01*	1.116	(1.071–1.163)	1.54E−07	1.118	(1.022–1.224)	3.68E−02
*C*07:01*	0.892	(0.851–0.936)	2.47E−06	0.802	(0.723–0.891)	2.77E−04
*DRB1*04:07*	1.178	(1.112–1.246)	1.78E−08	1.193	(1.058–1.347)	1.43E−02
*DQB1*03:02*	1.164	(1.118–1.211)	9.14E−14	1.195	(1.096–1.303)	3.47E−04
*DQB1*02:01*	0.919	(0.882–0.957)	4.01E−05	0.899	(0.821–0.984)	4.65E−02

Alleles with a frequency higher than 1% are shown. Logistic regression. OR, odds ratio; CI, confidence interval.
[a] $\alpha = 8.62E-04$.
[b] Remained significant after Benjamini-Hochberg (FDR) correction ($\alpha = 5.0E-02$).

meta-analysis revealed statistically significant associations for all three alleles tested ($p < 0.001$) (Table 4; Figure 2). Alleles *HLA-DRB1*04:03* (odds ratio [OR]: 1.20, 95% confidence interval [95% CI]: 1.14–1.25) and *HLA-DRB1*01:03* (OR: 0.60, 95% CI: 0.56–0.63) showed the strongest common effect sizes for risk and protection, respectively, while the allele *HLA-DRB1*07:01* (OR: 0.95, 95% CI: 0.93–0.97) presented a weaker effect size.

Further, we observed a strong dose effect for these alleles (Table S3). For example, the risk associated with two copies of *HLA-DRB1*04:03* was almost three times higher in EUR (OR = 3.083, 97.5% CI = 1.642–5.874) than a single copy. Likewise, *HLA-DRB1*01:03* homozygosity showed a protective effect almost three times higher than heterozygotes in the EUR subgroup (OR = 0.259, 97.5% CI = 0.076–0.661).

In summary, we observed clear evidence of *HLA* involvement in both risk and protection in CMV infection, with results replicating across independent registries and subpopulations.

Numerous *HLA* alleles show ancestry-specific association with CMV seropositivity

In addition to the three alleles replicating in multiple population subgroups, we observed a number of other alleles associated with CMV seropositivity in only a single population subgroup in our discovery (NMDP) cohort that replicated in the DKMS cohort (Table 2; Figure 1). The API subgroup presented the highest number of alleles associated with CMV (seven alleles, two risk), with the allele *HLA-DRB1*14:04* showing the largest risk effect size (OR = 1.202, 97.5% CI = 1.081–1.337, p = 6.68E−04) and the allele *HLA-DQB1*06:09* showing the largest protection effect size (OR = 0.791, 97.5% CI = 0.689–0.907, p = 8.36E−04). However, neither of these alleles were found to be associated with CMV serostatus in the other population subgroups evaluated. The HIS subgroup presented six associated alleles (four risk), as the biggest effect sizes were observed in the alleles *HLA-B*35:17* (OR = 1.312, 97.5% CI = 1.175–1.465, p = 1.37E−06) and *HLA-C*07:01* (OR = 0.892,

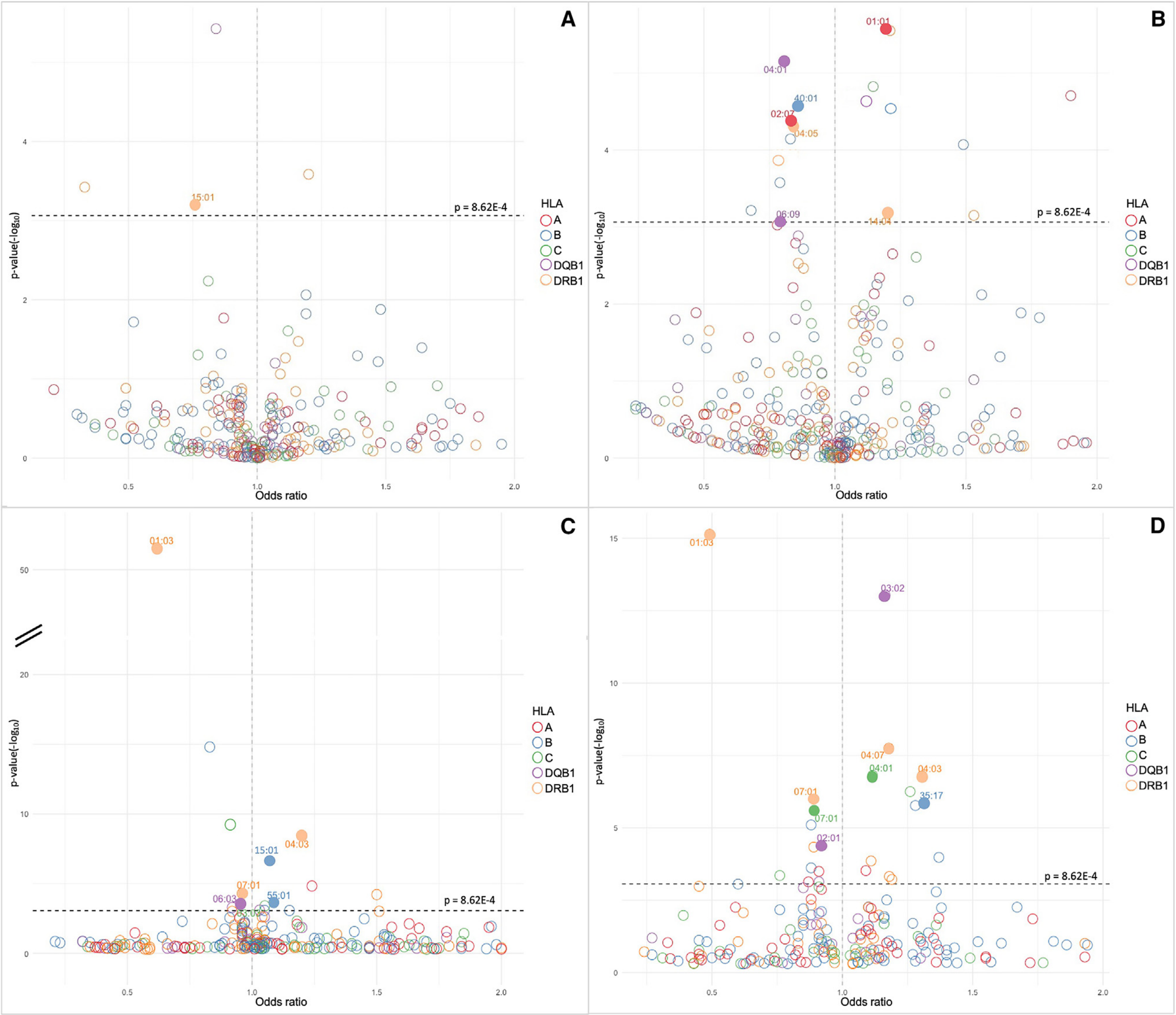

Figure 1. ***HLA*** **alleles associated with CMV in the NMDP cohort**
Filled circles: *HLA* alleles that significantly replicate in DKMS cohort. *p* value = 8.62E-04
(A) Black or African American.
(B) Asian or Pacific Islander.
(C) European descent.
(D) Hispanic or Latino.

97.5% CI = 0.851–0.936, p = 2.47E−07) for risk and protection, respectively. In the EUR subgroup, four alleles were found to be associated with CMV serostatus (three risk), where *HLA-B*55:01* (OR = 1.087, 97.5% CI = 1.040–1.135, p = 2.29E−04) presented the largest risk effect size and *HLA-DQB1*06:03* (OR = 0.953, 97.5% CI = 0.928–0.978, p = 3.18E−04) presented the largest protective effect. Interestingly, only *HLA-DRB1*15:01* (OR = 0.759, 97.5% CI = 0.646–0.887, p = 6.28E−04) was associated with CMV serostatus in the AFA subgroup.

In summary, beyond the four HLA alleles that showed consistent results across ancestries, we find extensive evidence of additional ancestry-specific *HLA* involvement in CMV serostatus in healthy individuals, which was replicated in independent cohorts.

HLA alleles associated with CMV seropositivity are predicted to have distinct peptide binding repertoires

We next sought to examine whether predicted patterns of peptide binding might explain some of the observed associations of *HLA* variation with CMV serostatus. Because *HLA-DRB1*01:03* and *HLA-DRB1*04:03* showed the strongest protective and risk effects, respectively, and replicated across population subgroups, we chose to focus on these alleles. These two alleles with opposite effects are members of different allelic families of *HLA-DRB1* and differ at 20 amino acid residues in the mature protein.[40] Interestingly, despite having a highly significant effect, each of these alleles is found at a low frequency (∼1%) in our US EUR populations. Meanwhile, their highly homologous counterparts, *HLA-DRB1*01:01* and *HLA-DRB1*04:01*, are

Table 3. **Significant *HLA* alleles associated with CMV that replicate in at least two population subgroups in both cohorts**

	NMDP			DKMS		
HLA allele	OR	$CI_{97.5\%}$	*p* value[a]	OR	$CI_{97.5\%}$	*p* value[b]
DRB1*01:03						
AFA	0.328	(0.170–0.587)	3.77E−04[c]	0.702	(0.336–1.477)	1.69E−01
API	0.503	(0.133–1.620)	2.68E−01	0.398	(0.051–2.463)	3.21E−01
EUR	0.618	(0.581–0.657)	2.81E−52[c]	0.669	(0.618–0.725)	3.19E−21[c,d]
HIS	0.491	(0.412–0.583)	7.26E−16[c]	0.602	(0.419–0.862)	1.62E−02[c,d]
DRB1*04:03						
AFA	1.129	(0.734–1.710)	5.73E−01	4.093	(1.875–10.267)	9.73E−04[c]
API	1.108	(1.010–1.214)	2.96E−02	1.123	(0.958–1.319)	1.56E−01
EUR	1.2	(1.129–1.275)	3.67E−09[c]	1.292	(1.189–1.404)	2.13E−08[c,d]
HIS	1.306	(1.182–1.443)	1.61E−07[c]	1.316	(1.063–1.636)	3.25E−02[c,d]
DRB1*07:01						
AFA	0.939	(0.862–1.022)	1.46E−01	0.997	(0.864–1.151)	9.64E−01
API	0.962	(0.903–1.025)	2.32E−01	1.055	(0.953–1.169)	3.00E−01
EUR	0.96	(0.942–0.979)	5.05E−05[c]	0.968	(0.942–0.994)	3.85E−02[c,d]
HIS	0.89	(0.850–0.933)	1.01E−06[c]	0.819	(0.738–0.909)	8.48E−04[c,d]

Logistic regression. OR, odds ratio; CI, confidence interval; AFA, Black or African American; API, Asian or Pacific Islander; EUR, European descent; HIS, Hispanic or Latino.
[a]$\alpha = 8.62E-04$.
[b]$\alpha = 5.0E-02$.
[c]Significant values.
[d]Remained significant after Benjamini-Hochberg (FDR) correction.

relatively common, each with frequencies of around 8% in the same population. Of note, there are only three amino acid differences between the mature proteins of *HLA-DRB1*01:01* and *HLA-DRB1*01:03* (residues 67, 70, and 71) and, likewise, *HLA-DRB1*04:01* and *HLA-DRB1*04:03* (residues 71, 74, and 86) (Figure 3).

To clarify the role of these individual amino acid differences in CMV infection, we examined the association of *HLA-DRB1* amino acids with CMV serostatus for the four population subgroups. Numerous *HLA-DRB1* amino acid residues showed an association with CMV seropositivity in the four population subgroups in our discovery cohort (Table S4). However, no individual amino acid demonstrated stronger effect sizes than those observed for the *HLA* allotypes, pointing to structural differences determined by the combination of these amino acid differences.

Thus, we were interested in understanding differential peptide binding between allotypes encoded by these relatively rare alleles that were strongly associated with CMV and those encoded by their closely related, more common alleles that showed no association with the virus. We predicted peptide binding for the three most immunogenic CMV proteins (pp65, IE-1, and IE-2)[36] with these four *HLA-DRB1* alleles using NetMHCIIpan 4.1 for *HLA* class II.[37] Notably, despite their overall similarity, the *HLA* allotypes associated with CMV seropositivity had peptide-binding predictions that were distinct from the closely related, non-associated allotypes (Table S5). For example, considering only the SB peptides that are not shared with the other *HLA* alleles included in this comparison, four pp65 peptides were predicted as SBs specifically for the *HLA-DRB1*01:03* allotype, but *HLA-DRB1*01:01* showed no SB peptides for this CMV protein. On the other hand, two unique IE-1 SB peptides were predicted for *HLA-DRB1*01:01* but none for *HLA-DRB1*01:03*, while for protein IE-2, there were three and two distinct SB peptides predicted for *HLA-DRB1*01:01* and *HLA-DRB1*01:03*, respectively. Therefore, *HLA-DRB1*01:03* and *HLA-DRB1*01:01* do not share SB peptides for pp65 and IE-1 CMV proteins. In turn, *HLA-DRB1*04:03* showed ten, nine, and eight exclusive SB peptides for pp65, IE-1, and IE-2 CMV proteins, respectively, while *HLA-DRB1*04:03* presented only three unique SB peptides for pp65 only and non-unique SBs for IE-1 and IE-2 CMV proteins. Strikingly, despite their similarity, for each protein considered, the number of overlapping SBs for each pair of alleles was always fewer than those that were specific to one allele or the other (Figure S2). Thus, it is clear that despite only three amino acid differences, the CMV-associate allotypes are predicted to have largely divergent peptide binding profiles relative to their more common counterparts for immunodominant CMV proteins.

Table 4. Trans-population meta-analysis

	Common effect model				Weight (%)				Heterogeneity		
	OR	$CI_{95\%}$	z	p value	AFA	API	EUR	HIS	I2 (%)	t2	p value
*HLA-DRB1*01:03*	0.599	0.565–0.634	−17.34	<0.0001	0.90	0.20	87.60	11.30	69.40	0.033	0.02
*HLA-DRB1*04:03*	1.197	1.144–1.252	7.85	<0.0001	1.10	23.80	54.90	20.20	47.70	0.003	0.125
*HLA-DRB1*07:01*	0.95	0.934–0.966	−5.92	<0.0001	3.90	7.20	75.50	13.30	66.20	0.001	0.031

OR, odds ratio; CI, confidence interval; AFA, Black or African American; API, Asian or Pacific Islander; EUR, European descent; HIS, Hispanic or Latino.

Polymorphisms in *HLA-DRB1* binding pockets P4 and P7 are associated with CMV seropositivity

We next sought to model these closely related (yet discordant with respect to their association with infection) pairs of *HLA-DRB1* alleles binding to CMV peptides to understand how their amino acid differences impact the electrostatic potential in the peptide-binding groove. Our modeling predictions included the α (*HLA-DRA*) and β (*HLA-DRB1*) chains and the CMV pp65 peptides with the biggest EL score difference between *HLA-DRB1*01:01* and *HLA-DRB1*01:03* as well as *HLA-DRB1*04:01* and *HLA-DRB1*04:03* (Figure 4; Table S5). The electrostatic potential for contacts between α and β chains with the CMV peptides is different for the four aforementioned *HLA-DRB1* alleles. The three residue differences between *HLA-DRB1*01:03* and *HLA-DRB1*01:01* (positions 67, 70, and 71) lie in the hypervariable region 3[41] (HVR3; ranging from 67 to 74 residues) of the *HLA-DRB1* gene, while two out of the three different amino acid residues between *HLA-DRB1*04:03* and *HLA-DRB1*04:01* (71 and 74) lie in HVR3 (Figures 3 and 4). In antigen presentation to T cells, the peptide is bound within a groove on the surface of the HLA protein. The peptide-binding groove of *HLA* class II molecules consists of nine different structural pockets (P1–P9), which accommodate the antigen peptide side chains.[42,43] The amino acid differences between *HLA-DRB1*01:01* and *HLA-DRB1*01:03* occur on binding pockets P4 (residues 70 and 71)[43] and P7 (residues 67 and 71).[43] Notably, for alleles *HLA-DRB1*04:03* and *HLA-DRB1*04:01*, the differences occur in the same P4 (residues 71 and 74)[43] and P7 (residue 74)[43] pockets.

These differences impact the charge and/or the volume of the molecule. For instance, the *HLA-DRB1*01:01* allele codes for glutamine at residue 70 (uncharged, medium volume of 143.8 Å^3),[44,45] while the *HLA-DRB1*01:03* allele codes for aspartic acid at residue 70 (negative charge, small volume of 111.1 Å^3).[44,45] For residue 71, *HLA-DRB1*01:01* codes for arginine (positive charge, large volume of 173.4 Å^3),[44,45] while *HLA-DRB1*01:03* codes for glutamic acid (negative charge, medium volume of 138.3 Å^3).[44,45] Likewise, for the *HLA-DRB1*04:03* and *HLA-DRB1*04:01* alleles, residue 74 presents the biggest difference in terms of charge and volume, coding for alanine (uncharged, very small volume of 88.6 Å^3)[44,45] in *HLA-DRB1*04:01* and for glutamic acid (negative charge, medium volume of 138.3 Å^3)[44,45] in *HLA-DRB1*04:03*. Overall, only *HLA-DRB1*01:03* results in a negative charge at residue 71, while all other alleles tested here present a positive charge (Table S6), impacting in the electrostatic potential in the binding pockets P4 and P7 (Figure 4B).

In conclusion, we find support for structural differences between rare CMV-associated and common non-associated *HLA* allotypes that may explain differences in binding to CMV-derived peptides.

Discussion

Uncontrolled CMV replication in immunocompromised patients can lead to end-organ diseases such as pneumonitis, retinitis, hepatitis, or gastroenteritis. CMV infection in solid organ transplant patients can lead to either direct effects, such as end-organ disease, or indirect effects, including bacterial and fungal coinfections[24,46] and GVHD, in HSCT patients.[25] In addition, CMV is associated with poor transplantation outcomes that include higher mortality.[23] Furthermore, congenital CMV infection is the most common congenital infection in industrialized countries.[47,48] Congenital CMV infection is neglected mainly because most maternal and newborn infections are asymptomatic, and sequelae from congenital CMV infection are frequently delayed in onset, making retrospective diagnosis difficult.[5] The socioeconomic impact of CMV as the commonest nongenetic cause of childhood hearing loss and a significant cause of neurodevelopmental delay is underappreciated.[48] Symptomatic neonates can develop hearing loss and cognitive and motor delay as permanent sequelae.[49] As CMV is the most common intrauterine infection, effective vaccine development is a high priority for the protection of both newborns and transplant recipients.[49–51]

Understanding the genetic and immunological underpinnings of CMV infection is important for public health measures, vaccine design, and therapeutic development. Leveraging two extremely large cohorts, we were able to identify *HLA* alleles associated with both risk and protection for CMV serostatus among four different broad US population subgroups. Revealing *HLA* alleles associated with CMV can provide important insights into the immunological response to CMV infection. To the best of our knowledge, this retrospective case-control study represents the most well-powered examination of *HLA* and CMV to date, as well as one of the few in an immunocompetent population, including high-resolution *HLA* genotyping

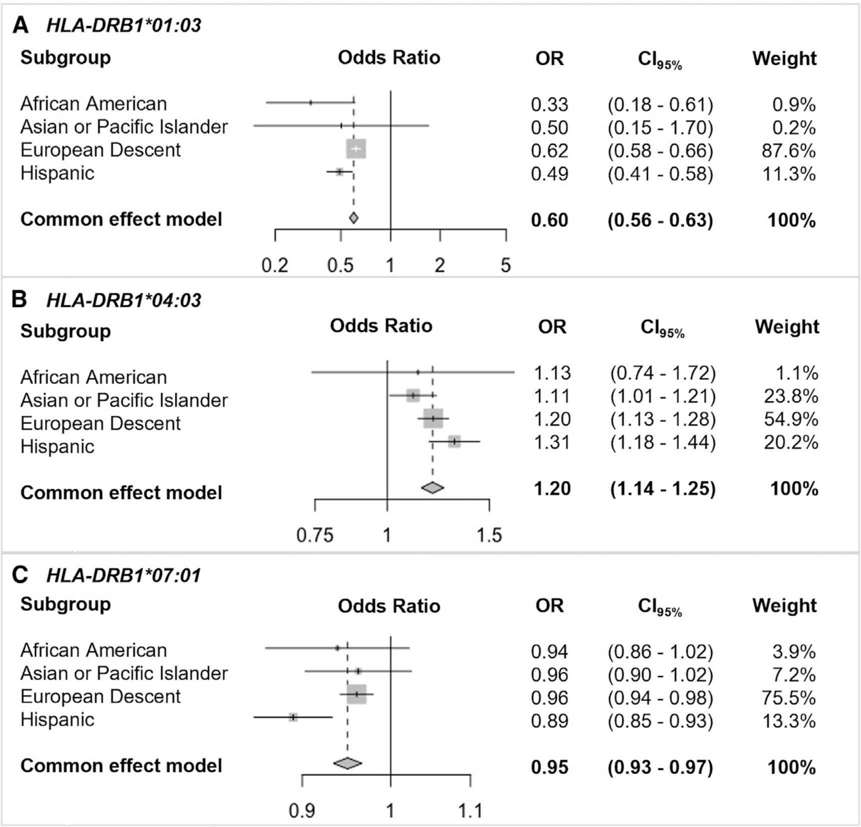

Figure 2. Trans-population meta-analysis forest plot
Common effect model p value < 0.0001
(A) *HLA-DRB1*01:03*.
(B) *HLA-DRB1*04:03*.
(C) *HLA-DRB1*07:01*.

and CMV serostatus data, as well as important demographic and socioeconomic variables, from more than a half-million individuals.

Twenty-one *HLA* alleles were significantly associated with CMV serostatus in both our discovery and replication cohorts, most with relatively small effect sizes. Focusing on those alleles that were significantly associated with CMV in at least two different population subgroups, we highlight the role of three class II alleles (*HLA-DRB1*01:03*, *HLA-DRB1*04:03*, and *HLA-DRB1*07:01*). A common effect trans-population meta-analysis showed effect sizes consistently higher for the highlighted alleles than those that did not replicate across multiple ancestries, where *HLA-DRB1*01:03* showed the strongest protective effect size and *HLA-DRB1*04:03* presented the strongest risk effect, with evidence for dose effects. Of particular interest is the fact that while both alleles are found at a low frequency in our largest population subgroup (EUR), their closely related counterparts *HLA-DRB1*01:01* and *HLA-DRB1*04:01* are quite common and yet show no association with CMV serostatus.

All three different amino acid residues between *HLA-DRB1*01:03* and *HLA-DRB1*01:01* lie in the HVR3 of the *HLA-DRB1* gene, while two out of the three different amino acid residues lie in HVR3 in relation to *HLA-DRB1*04:03* and *HLA-DRB1*04:01* alleles. Differences in these polymorphic residues can impact the charge and/or the volume of the molecule as is shown in Figure 4. The polymorphic residue 71 is part of both pockets P4 and P7 and codes for a negatively charged amino acid (glutamic acid) in the *HLA-DRB1*01:03* allele, while the other three *HLA-DRB1* alleles code for a positively charged amino acid. The structural pockets of the *HLA* class II binding groove exert a major influence on peptide binding and its recognition by T cells.[52] The polymorphic residues 70, 71, and 74 of the *HLA-DRB1* protein, located in pocket P4 of the β chain, play a central role in the recognition of the *HLA-DRB1*/peptide complex by the $CD4^+$ helper T cell.[53] Thus, it seems that particular features of these alleles, which differ by only three amino acids from their close relatives, are responsible for their relationship with CMV infection.

Rovito et al. evaluated the CMV viral load in children affected by congenital CMV. In their study, the median viral load was significantly lower in the children carrying *HLA-DRB1*04* compared to the *HLA-DRB1*04*-negative children, indicating a protective role against CMV.[54] However, the genotyping resolution in that study would not have detected the differential association between *HLA-DRB1*04:01* (neutral) and *HLA-DRB1*04:03* (risk) reported here. In other disease contexts, different associations of these closely related alleles have been shown. For example, *HLA-DRB1*04:01* confers risk for type 1 diabetes (MIM: 222100), while *HLA-DRB1*04:03* confers protection.[55] Our own work has shown that *HLA-DRB1*04:01* is protective in Parkinson disease (MIM: 168600), while *HLA-DRB1*04:03* is not,[56] as part of a pattern related to the *HLA-DRB1* "shared epitope" (SE). The SE was first recognized due to an association between a five-amino-acid sequence motif in residues 70–74 of *HLA-DRB1* alleles with severe rheumatoid arthritis (RA [MIM: 180300]),[57,58] with the *HLA-DRB1*04* allelic group representing the most common SE-coding alleles.[59] Interestingly, two out the three amino acid differences (residues 71 and 74) between *HLA-DRB1*04:01* (QKRAA, SE positive) and *HLA-DRB1*04:03* (QRRAE, SE negative) lie in the SE region. While CMV infection has not been related as a trigger for RA, there is a possible association between CMV and the pathophysiology of RA, being an aggravating factor of inflammation in RA while protecting from bone erosion.[60] Interaction between SE and low levels of both anti-EBV (Epstein-Barr virus) and anti-B19 (parvovirus B19) antibodies has also been demonstrated.[61]

A

```
AA Pos.                    -21        -11         -1         10         20         30         40         50         60         70
DRB1*01:01:01:01     MVCLKLPGG SCMTALTVTL MVLSSPLALA GDTRPRFLWQ LKFECHFFNG TERVRLLERC IYNQEESVRF DSDVGEYRAV TELGRPDAEY WNSQKDLLEQ
DRB1*01:03:01:01     --------- ---------- ---------- ---------- ---------- ---------- ---------- ---------- ---------- ------I--D

AA Pos.                     80         90        100        110        120        130        140        150        160        170
DRB1*01:01:01:01    RRAAVDTYCR HNYGVGESFT VQRRVEPKVT VYPSKTQPLQ HHNLLVCSVS GFYPGSIEVR WFRNGQEEKA GVVSTGLIQN GDWTFQTLVM LETVPRSGEV
DRB1*01:03:01:01    E--------- ---------- ---------- ---------- ---------- ---------- ---------- ---------- ---------- ----------

AA Pos.                    180        190        200        210        220        230
DRB1*01:01:01:01    YTCQVEHPSV TSPLTVEWRA RSESAQSKML SGVGGFVLGL LFLGAGLFIY FRNQKGHSGL QPTGFLS
DRB1*01:03:01:01    ---------- ---------- ---------- ---------- ---------- ---------- -------
```

B

```
AA Pos.                    -21        -11         -1         10         20         30         40         50         60         70
DRB1*04:01:01:01     MVCLKFPGG SCMAALTVTL MVLSSPLALA GDTRPRFLEQ VKHECHFFNG TERVRFLDRY FYHQEEYVRF DSDVGEYRAV TELGRPDAEY WNSQKDLLEQ
DRB1*04:03:01:01     --------- ---------- ---------- ---------- ---------- ---------- ---------- ---------- ---------- ----------

AA Pos.                     80         90        100        110        120        130        140        150        160        170
DRB1*04:01:01:01    KRAAVDTYCR HNYGVGESFT VQRRVYPEVT VYPAKTQPLQ HHNLLVCSVN GFYPGSIEVR WFRNGQEEKT GVVSTGLIQN GDWTFQTLVM LETVPRSGEV
DRB1*04:03:01:01    R--E------ -----V---- ---------- ---------- ---------- ---------- ---------- ---------- ---------- ----------

AA Pos.                    180        190        200        210        220        230
DRB1*04:01:01:01    YTCQVEHPSL TSPLTVEWRA RSESAQSKML SGVGGFVLGL LFLGAGLFIY FRNQKGHSGL QPTGFLS
DRB1*04:03:01:01    ---------- ---------- ---------- ---------- ---------- ---------- -------
```

Figure 3. ***HLA-DRB1*** **protein sequence alignment**
(A) *HLA-DRB1*01:01* and *HLA-DRB1*01:03*. Differences in residues 67, 70, and 71 are shown.
(B) *HLA-DRB1*04:01* and *HLA-DRB1*04:03*. Differences in amino acid residues 71, 74, and 86 are shown.
aa, amino acid.

Similarly, two amino acid differences (residues 70 and 71) between *HLA-DRB1*01:01* (QRRAA, SE positive) and *HLA-DRB1*01:03* (DERAA, SE negative) alleles lie in the SE region. *HLA-DRB1*01:03* has been shown to be associated with risk for Crohn disease (MIM: 266600) and ulcerative colitis (MIM: 191390) in several large studies of individuals with inflammatory bowel disease (IBD).[62] The authors suggest that this allele is critically involved in determining the colonic immune response to local flora.[62] Here, we demonstrated, for the first time, a protective effect of the *HLA-DRB1*01:03* allele against CMV infection. While there is no clear evidence for a role of CMV infection across autoimmune diseases, it has been suggested that CMV can trigger systemic lupus erythematosus (SLE [MIM: 152700])[63,64] or aggravate the disease.[65] However, it is not clear whether CMV infection triggers SLE or occurs simultaneously with or after SLE onset.[66] Nevertheless, there is some evidence for an intriguing interplay between adaptive immune responses to CMV and manifestations of certain autoimmune disorders, for example, in RA and SLE.[67]

The CMV proteins pp65, IE-1, and IE-2 are the major targets of the cellular immune response.[36] In our peptide-binding prediction analysis, IE-2 showed a higher number of SB peptides among the CMV proteins tested with the four *HLA-DRB1* allotypes, and IE-1 presented the lowest number. Interestingly, the strongest protective *HLA-DRB1*01:03* allele in our analysis showed no SB peptides for IE-1 protein. The peptides IE-$2_{408–422}$ (KGIQIIYTRNHEVKS) and IE-$2_{438–452}$ (ALSTPFLMEHTMPVT), previously validated for *HLA-DRB1*07:01* and *HLA-DRB1*01:01* restriction, respectively,[68] were predicted as SBs for *HLA-DRB1*01:03* in our analysis. Slezak et al. characterized several pp65 peptides in 20 healthy CMV-seropositive subjects,[69] but none of the peptides overlap with the SB peptides predicted in our analysis. The subjects included in their study presented diverse *HLA-DRB1* allotypes, which in part explain the different peptides predicted in our study. In their study, no IE-1 class II epitopes were identified,[69] while in our prediction analysis, IE-1 presented the lowest number of SBs—and no SBs for *HLA-DRB1*01:03*. The lack of naturally occurring $CD4^+$ T cell responses to IE-1 suggests that its epitopes may not be good candidates to trigger immune response.[69] In another study including individuals with diverse *HLA* alleles, CMV pp65 peptide-specific T cell lines selectively respond to a restricted number of CMV pp65 epitopes presented by a limited number of prevalent *HLA* alleles.[70] For instance, the epitopes presented by *HLA-B*07:02* and *HLA-A*02:01* alleles consistently elicit immunodominant CMV pp65 peptide-specific T-cells.[70] Yet, all immunodominant CMV pp65 peptide-specific T cells exhibiting *HLA*-restricted cytotoxicity against epitope-loaded targets, except those responding to epitopes presented by *HLA-B*35* alleles, were ineffective in controlling CMV infections.[70] Identifying immunodominant epitopes in major immunogenic CMV proteins and their *HLA* allelic restrictions could be useful for adoptive immune therapy and vaccine development.[71]

As a potential limitation of the study, we acknowledge that our reliance on self-reported ancestry may be less ideal than using genome-wide data to assess population substructure, for instance. However, we also note that we have addressed the question of the accuracy of self-reported ancestry in the NMDP cohort (discovery) in four published studies[72–75] that included consideration of genome-wide markers. While imperfect, we found that

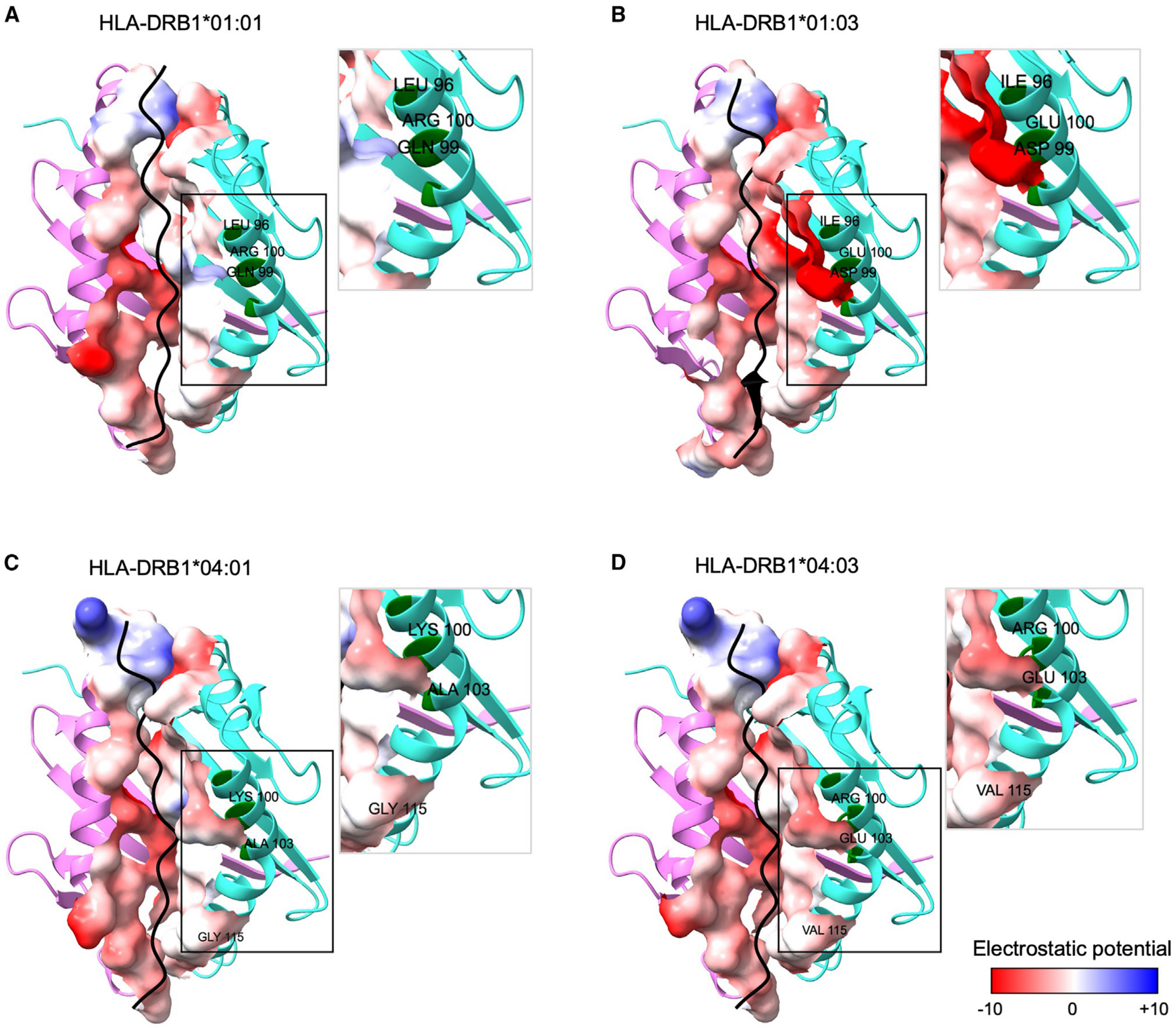

Figure 4. *HLA-DRB1* binding groove
HLA-DRB1 binding groove and CMV pp65 DTPVLPHETRLLQTG peptide for *HLA-DRB1*01:01* and *HLA-DRB1*01:03* (A and B) and pp65 VSQYTPDSTPCHRGD peptide for *HLA-DRB1*04:01* and *HLA-DRB1*04:03* (C and D).
(A) *HLA-DRB1*01:01*.
(B) *HLA-DRB1*01:03*.
(C) *HLA-DRB1*04:01*.
(D) *HLA-DRB1*04:03*.
α chain: violet. β chain: turquoise. CMV pp65 peptides: black. Blue: positive charges. White: neutral charges. Red: negative charges. Variant peptides between *HLA-DRB1*01:01* and *HLA-DRB1*01:03* as well as *HLA-DRB1*04:01* and *HLA-DRB1*04:03* are highlighted in green and labeled.

self-reported identification is a reasonable proxy for *HLA* genetic variation in this cohort, and these self-reported categories are fundamental to the matching algorithms used to locate suitable *HLA* matches for patients from among the millions of registered donors. We also note that we only report associations that replicated in a completely independent cohort from a second donor registry with different recruitment processes and demographic makeups, which we feel minimizes the likelihood that the reported associations are spurious or related to underlying population substructures.

An additional limitation is the lower rates of CMV seropositivity in our cohorts relative to the general US population, which may limit its generalizability. We note that several factors in donor recruitment may be responsible for this differential: donors are, on average, younger, healthier, and have higher SES status than the general population. Overall, recruitment is skewed toward young males (and European ancestry, despite efforts to improve diversity), who are known to have a much lower CMV-positive rate in all three dimensions (age, sex, and ancestry).[76]

In summary, we present highly significant associations of *HLA* class I and class II alleles with CMV serostatus in two large cohorts of healthy people. Our results contribute to our understanding of the role of *HLA* variation in CMV infection and may provide a basis for vaccine development or therapeutic targets in the future.

Data and code availability

The full raw data that support the findings of this study are available from the corresponding author upon reasonable request. Data are located in controlled access data storage at the University of California, San Francisco.

Acknowledgments

The authors wish to thank Rachel Rutihauser for helpful discussions. We also thank the volunteer donors in the NMDP and DKMS registries. This work was supported by NIH R01AI158861 (J.A.H.).

Author contributions

J.A.H. and M.M. conceived this work; J.A.B. and J.A.H. undertook the formal analysis and investigation and wrote the original draft; J.S., A.H.S., and M.M. undertook the dataset collection and curation; J.A.H. obtained resources, conducted project administration, and supervised the study; and all authors reviewed and edited the final manuscript.

Declaration of interests

The authors declare no competing interests.

Supplemental information

Supplemental information can be found online at https://doi.org/10.1016/j.ajhg.2025.02.007.

Web resources

GenBank, https://www.ncbi.nlm.nih.gov/genbank/
OMIM, https://www.omim.org

Received: September 12, 2024
Accepted: February 7, 2025
Published: March 5, 2025

References

1. Zuhair, M., Smit, G.S.A., Wallis, G., Jabbar, F., Smith, C., Devleesschauwer, B., and Griffiths, P. (2019). Estimation of the worldwide seroprevalence of cytomegalovirus: A systematic review and meta-analysis. Rev. Med. Virol. *29*, e2034. https://doi.org/10.1002/rmv.2034.
2. Rubin, R.H. (2007). The pathogenesis and clinical management of cytomegalovirus infection in the organ transplant recipient: the end of the "silo hypothesis". Curr. Opin. Infect. Dis. *20*, 399–407. https://doi.org/10.1097/QCO.0b013e328285a358.
3. Cannon, M.J., Schmid, D.S., and Hyde, T.B. (2010). Review of cytomegalovirus seroprevalence and demographic characteristics associated with infection. Rev. Med. Virol. *20*, 202–213. https://doi.org/10.1002/rmv.655.
4. Osawa, R., and Singh, N. (2009). Cytomegalovirus infection in critically ill patients: a systematic review. Crit. Care *13*, R68. https://doi.org/10.1186/cc7875.
5. Manicklal, S., Emery, V.C., Lazzarotto, T., Boppana, S.B., and Gupta, R.K. (2013). The "Silent" global burden of congenital cytomegalovirus. Clin. Microbiol. Rev. *26*, 86–102. https://doi.org/10.1128/CMR.00062-12.
6. Feinstein, L., Douglas, C.E., Stebbins, R.C., Pawelec, G., Simanek, A.M., and Aiello, A.E. (2016). Does cytomegalovirus infection contribute to socioeconomic disparities in all-cause mortality? Mech. Ageing Dev. *158*, 53–61. https://doi.org/10.1016/j.mad.2016.06.001.
7. Fowler, K., Mucha, J., Neumann, M., Lewandowski, W., Kaczanowska, M., Grys, M., Schmidt, E., Natenshon, A., Talarico, C., Buck, P.O., and Diaz-Decaro, J. (2022). A systematic literature review of the global seroprevalence of cytomegalovirus: possible implications for treatment, screening, and vaccine development. BMC Public Health *22*, 1659. https://doi.org/10.1186/s12889-022-13971-7.
8. Ssentongo, P., Hehnly, C., Birungi, P., Roach, M.A., Spady, J., Fronterre, C., Wang, M., Murray-Kolb, L.E., Al-Shaar, L., Chinchilli, V.M., et al. (2021). Congenital Cytomegalovirus Infection Burden and Epidemiologic Risk Factors in Countries With Universal Screening A Systematic Review and Meta-analysis. JAMA Netw. Open *4*, E2120736. https://doi.org/10.1001/jamanetworkopen.2021.20736.
9. Cheung, A.K.L., Abendroth, A., Cunningham, A.L., and Slobedman, B. (2006). Viral gene expression during the establishment of human cytomegalovirus latent infection in myeloid progenitor cells. Blood *108*, 3691–3699. https://doi.org/10.1182/blood-2005-12-026682.
10. Beersma, M.F., Bijlmakers, M.J., and Ploegh, H.L. (1993). Human cytomegalovirus down-regulates HLA class I expression by reducing the stability of class I H chains. J. Immunol. *151*, 4455–4464.
11. Shiina, T., Hosomichi, K., Inoko, H., and Kulski, J.K. (2009). The HLA genomic loci map: expression, interaction, diversity and disease. J. Hum. Genet. *54*, 15–39. https://doi.org/10.1038/jhg.2008.5.
12. Lenz, T.L., Spirin, V., Jordan, D.M., and Sunyaev, S.R. (2016). Excess of Deleterious Mutations around HLA Genes Reveals Evolutionary Cost of Balancing Selection. Mol. Biol. Evol. *33*, 2555–2564. https://doi.org/10.1093/molbev/msw127.
13. Martin, M.P., and Carrington, M. (2013). Immunogenetics of HIV disease. Immunol. Rev. *254*, 245–264. https://doi.org/10.1111/imr.12071.
14. Blackwell, J.M., Jamieson, S.E., and Burgner, D. (2009). HLA and infectious diseases. Clin. Microbiol. Rev. *22*, 370–385. https://doi.org/10.1128/CMR.00048-08.
15. Augusto, D.G., and Hollenbach, J.A. (2022). HLA variation and antigen presentation in COVID-19 and SARS-CoV-2 infection. Curr. Opin. Immunol. *76*, 102178. https://doi.org/10.1016/j.coi.2022.102178.
16. Augusto, D.G., Murdolo, L.D., Chatzileontiadou, D.S.M., Sabatino, J.J., Yusufali, T., Peyser, N.D., Butcher, X., Kizer, K., Guthrie, K., Murray, V.W., et al. (2023). A common allele of HLA is associated with asymptomatic SARS-CoV-2 infection. Nature *620*, 128–136. https://doi.org/10.1038/s41586-023-06331-x.

17. Palmer, W.H., and Norman, P.J. (2023). The impact of HLA polymorphism on herpesvirus infection and disease. Immunogenetics *75*, 231–247. https://doi.org/10.1007/s00251-022-01288-z.
18. Du, J., Liu, J., Gu, J., and Zhu, P. (2007). HLA-DRB1*09 Is Associated with Increased Incidence of Cytomegalovirus Infection and Disease after Allogeneic Hematopoietic Stem Cell Transplantation. Biol. Blood Marrow Transplant. *13*, 1417–1421. https://doi.org/10.1016/j.bbmt.2007.09.003.
19. Futohi, F., Saber, A., Nemati, E., Einollahi, B., and Rostami, Z. (2015). Human leukocyte antigen alleles and cytomegalovirus infection after renal transplantation. Nephrourol. Mon. *7*, e31635. https://doi.org/10.5812/numonthly.31635.
20. Kawase, T., Tanaka, H., Kojima, H., Uchida, N., Ohashi, K., Fukuda, T., Ozawa, Y., Ikegame, K., Eto, T., Mori, T., et al. (2019). Impact of High-Frequency HLA Haplotypes on Clinical Cytomegalovirus Reactivation in Allogeneic Hematopoietic Stem Cell Transplantation. Biol. Blood Marrow Transplant. *25*, 2482–2489. https://doi.org/10.1016/j.bbmt.2019.07.042.
21. Prem, S., Remberger, M., Alotaibi, A., Lam, W., Law, A.D., Kim, D.D.H., Michelis, F.V., Al-Shaibani, Z., Lipton, J.H., Mattsson, J., et al. (2022). Relationship between certain HLA alleles and the risk of cytomegalovirus reactivation following allogeneic hematopoietic stem cell transplantation. Transpl. Infect. Dis. *24*, e13879. https://doi.org/10.1111/tid.13879.
22. Lee, S.J., Klein, J., Haagenson, M., Baxter-Lowe, L.A., Confer, D.L., Eapen, M., Fernandez-Vina, M., Flomenberg, N., Horowitz, M., Hurley, C.K., et al. (2007). High-resolution donor-recipient HLA matching contributes to the success of unrelated donor marrow transplantation. Blood *110*, 4576–4583. https://doi.org/10.1182/blood-2007-06-097386.
23. Teira, P., Battiwalla, M., Ramanathan, M., Barrett, A.J., Ahn, K.W., Chen, M., Green, J.S., Saad, A., Antin, J.H., Savani, B.N., et al. (2016). Early cytomegalovirus reactivation remains associated with increased transplant-related mortality in the current era: a CIBMTR analysis. Blood *127*, 2427–2438. https://doi.org/10.1182/blood-2015-11-679639.
24. Nichols, W.G., Corey, L., Gooley, T., Davis, C., and Boeckh, M. (2002). High Risk of Death Due to Bacterial and Fungal Infection among Cytomegalovirus (CMV)–Seronegative Recipients of Stem Cell Transplants from Seropositive Donors: Evidence for Indirect Effects of Primary CMV Infection. J. Infect. Dis. *185*, 273–282. https://doi.org/10.1086/338624.
25. Cantoni, N., Hirsch, H.H., Khanna, N., Gerull, S., Buser, A., Bucher, C., Halter, J., Heim, D., Tichelli, A., Gratwohl, A., and Stern, M. (2010). Evidence for a Bidirectional Relationship between Cytomegalovirus Replication and acute Graft-versus-Host Disease. Biol. Blood Marrow Transplant. *16*, 1309–1314. https://doi.org/10.1016/j.bbmt.2010.03.020.
26. Centers for Disease Control and Prevention, Infectious Disease Society of America, and American Society of Blood and Marrow Transplantation (2000). Guidelines for preventing opportunistic infections among hematopoietic stem cell transplant recipients. MMWR Recomm. Rep. (Morb. Mortal. Wkly. Rep.) *49*, 1–125. CE1-7.
27. Madbouly, A., Gragert, L., Freeman, J., Leahy, N., Gourraud, P.-A., Hollenbach, J.A., Kamoun, M., Fernandez-Vina, M., and Maiers, M. (2014). Validation of statistical imputation of allele-level multilocus phased genotypes from ambiguous HLA assignments. Tissue Antigens *84*, 285–292. https://doi.org/10.1111/tan.12390.
28. Lange, V., Böhme, I., Hofmann, J., Lang, K., Sauter, J., Schöne, B., Paul, P., Albrecht, V., Andreas, J.M., Baier, D.M., et al. (2014). Cost-efficient high-throughput HLA typing by MiSeq amplicon sequencing. BMC Genom. *15*, 63. https://doi.org/10.1186/1471-2164-15-63.
29. Schöfl, G., Lang, K., Quenzel, P., Böhme, I., Sauter, J., Hofmann, J.A., Pingel, J., Schmidt, A.H., and Lange, V. (2017). 2.7 million samples genotyped for HLA by next generation sequencing: lessons learned. BMC Genom. *18*, 161. https://doi.org/10.1186/s12864-017-3575-z.
30. Diez Roux, A.V., and Mair, C. (2010). Neighborhoods and health. Ann. N. Y. Acad. Sci. *1186*, 125–145. Preprint at Blackwell Publishing Inc. https://doi.org/10.1111/j.1749-6632.2009.05333.x.
31. Andrews, M.R., Tamura, K., Claudel, S.E., Xu, S., Ceasar, J.N., Collins, B.S., Langerman, S., Mitchell, V.M., Baumer, Y., and Powell-Wiley, T.M. (2020). Geospatial analysis of neighborhood deprivation index (NDI) for the United States by county. J. Maps *16*, 101–112. https://doi.org/10.1080/17445647.2020.1750066.
32. Posit. (2024). RStudio: Integrated Development Environment for R (Boston, MA: Posit Software, PBC). http://www.posit.co/.
33. Schwarzer, G., Carpenter, J.R., and Rücker, G. (2015). Meta-Analysis with R (Springer International Publishing). https://doi.org/10.1007/978-3-319-21416-0.
34. Wickham, H. (2009). ggplot2 (Springer). https://doi.org/10.1007/978-0-387-98141-3.
35. Pappas, D.J., Marin, W., Hollenbach, J.A., and Mack, S.J. (2016). Bridging ImmunoGenomic Data Analysis Workflow Gaps (BIGDAWG): An integrated case-control analysis pipeline. Hum. Immunol. *77*, 283–287. https://doi.org/10.1016/j.humimm.2015.12.006.
36. Sylwester, A.W., Mitchell, B.L., Edgar, J.B., Taormina, C., Pelte, C., Ruchti, F., Sleath, P.R., Grabstein, K.H., Hosken, N.A., Kern, F., et al. (2005). Broadly targeted human cytomegalovirus-specific CD4+ and CD8+ T cells dominate the memory compartments of exposed subjects. J. Exp. Med. *202*, 673–685. https://doi.org/10.1084/jem.20050882.
37. Reynisson, B., Barra, C., Kaabinejadian, S., Hildebrand, W.H., Peters, B., Nielsen, M., Nielsen, M., and Nielsen, M. (2020). Improved Prediction of MHC II Antigen Presentation through Integration and Motif Deconvolution of Mass Spectrometry MHC Eluted Ligand Data. J. Proteome Res. *19*, 2304–2315. https://doi.org/10.1021/acs.jproteome.9b00874.
38. Abramson, J., Adler, J., Dunger, J., Evans, R., Green, T., Pritzel, A., Ronneberger, O., Willmore, L., Ballard, A.J., Bambrick, J., et al. (2024). Accurate structure prediction of biomolecular interactions with AlphaFold 3. Nature *630*, 493–500. https://doi.org/10.1038/s41586-024-07487-w.
39. Meng, E.C., Goddard, T.D., Pettersen, E.F., Couch, G.S., Pearson, Z.J., Morris, J.H., and Ferrin, T.E. (2023). UCSF ChimeraX: Tools for structure building and analysis. Protein Sci. *32*, e4792. https://doi.org/10.1002/pro.4792.
40. Barker, D.J., Maccari, G., Georgiou, X., Cooper, M.A., Flicek, P., Robinson, J., and Marsh, S.G.E. (2023). The IPD-IMGT/HLA Database. Nucleic Acids Res. *51*, D1053–D1060. https://doi.org/10.1093/nar/gkac1011.
41. Reinsmoen, N.L., and Bach, F.H. (1990). Structural model for T-cell recognition of HLA class II—associated alloepitopes. Hum. Immunol. *27*, 51–72. https://doi.org/10.1016/0198-8859(90)90095-7.

42. McFarland, B.J., and Beeson, C. (2002). Binding interactions between peptides and proteins of the class II Major Histocompatibility Complex. Med. Res. Rev. *22*, 168–203. https://doi.org/10.1002/med.10006.
43. Bondinas, G.P., Moustakas, A.K., and Papadopoulos, G.K. (2007). The spectrum of HLA-DQ and HLA-DR alleles, 2006: A listing correlating sequence and structure with function. Immunogenetics *59*, 539–553. https://doi.org/10.1007/s00251-007-0224-8.
44. Pommié, C., Levadoux, S., Sabatier, R., Lefranc, G., and Lefranc, M. (2004). IMGT standardized criteria for statistical analysis of immunoglobulin V-REGION amino acid properties. J. Mol. Recognit. *17*, 17–32. https://doi.org/10.1002/jmr.647.
45. Zamyatnin, A.A. (1972). Protein volume in solution. Prog. Biophys. Mol. Biol. *24*, 107–123. https://doi.org/10.1016/0079-6107(72)90005-3.
46. Yong, M.K., Ananda-Rajah, M., Cameron, P.U., Morrissey, C.O., Spencer, A., Ritchie, D., Cheng, A.C., Lewin, S.R., and Slavin, M. (2017). Cytomegalovirus Reactivation Is Associated with Increased Risk of Late-Onset Invasive Fungal Disease after Allogeneic Hematopoietic Stem Cell Transplantation: A Multicenter Study in the Current Era of Viral Load Monitoring. Biol. Blood Marrow Transplant. *23*, 1961–1967. https://doi.org/10.1016/j.bbmt.2017.07.025.
47. Dollard, S.C., Grosse, S.D., and Ross, D.S. (2007). New estimates of the prevalence of neurological and sensory sequelae and mortality associated with congenital cytomegalovirus infection. Rev. Med. Virol. *17*, 355–363. https://doi.org/10.1002/rmv.544.
48. Demmler-Harrison, G.J. (2009). Congenital cytomegalovirus: Public health action towards awareness, prevention, and treatment. J. Clin. Virol. *46*, S1–S5. https://doi.org/10.1016/j.jcv.2009.10.007.
49. Vaccines for the 21st Century (2000). (National Academies Press) 10.17226/5501.
50. Arvin, A.M., Fast, P., Myers, M., Plotkin, S., Rabinovich, R.; and National Vaccine Advisory Committee (2004). Vaccine development to prevent cytomegalovirus disease: report from the National Vaccine Advisory Committee. Clin. Infect. Dis. *39*, 233–239. https://doi.org/10.1086/421999.
51. Anderholm, K.M., Bierle, C.J., and Schleiss, M.R. (2016). Cytomegalovirus Vaccines: Current Status and Future Prospects. Drugs *76*, 1625–1645. https://doi.org/10.1007/s40265-016-0653-5.
52. Stern, L.J., Brown, J.H., Jardetzky, T.S., Gorga, J.C., Urban, R.G., Strominger, J.L., and Wiley, D.C. (1994). Crystal structure of the human class II MHC protein HLA-DR1 complexed with an influenza virus peptide. Nature *368*, 215–221. https://doi.org/10.1038/368215a0.
53. Fu, X.T., Bono, C.P., Woulfe, S.L., Swearingen, C., Summers, N.L., Sinigaglia, F., Sette, A., Schwartz, B.D., and Karr, R.W. (1995). Pocket 4 of the HLA-DR(α,β 1*0401) molecule is a major determinant of T cells recognition of peptide. J. Exp. Med. *181*, 915–926. https://doi.org/10.1084/jem.181.3.915.
54. Rovito, R., Claas, F.H.J., Haasnoot, G.W., Roelen, D.L., Kroes, A.C.M., and Vossen, A.C.T.M. (2018). Maternal and child human leukocyte antigens in congenital cytomegalovirus infection. J. Reprod. Immunol. *126*, 39–45. https://doi.org/10.1016/j.jri.2018.01.002.
55. Zhao, L.P., Papadopoulos, G.K., Lybrand, T.P., Moustakas, A.K., Bondinas, G.P., Carlsson, A., Larsson, H.E., Ludvigsson, J., Marcus, C., Persson, M., et al. (2021). The KAG motif of HLA-DRB1 (β71, β74, β86) predicts seroconversion and development of type 1 diabetes. EBioMedicine *69*, 103431. https://doi.org/10.1016/j.ebiom.2021.103431.
56. Hollenbach, J.A., Norman, P.J., Creary, L.E., Damotte, V., Montero-Martin, G., Caillier, S., Anderson, K.M., Misra, M.K., Nemat-Gorgani, N., Osoegawa, K., et al. (2019). A specific amino acid motif of HLA-DRB1 mediates risk and interacts with smoking history in Parkinson's disease. Proc. Natl. Acad. Sci. USA *116*, 7419–7424. https://doi.org/10.1073/pnas.1821778116.
57. Gregersen, P.K., Silver, J., and Winchester, R.J. (1987). The shared epitope hypothesis. an approach to understanding the molecular genetics of susceptibility to rheumatoid arthritis. Arthritis Rheum. *30*, 1205–1213. https://doi.org/10.1002/art.1780301102.
58. Turesson, C., Schaid, D.J., Weyand, C.M., Jacobsson, L.T.H., Goronzy, J.J., Petersson, I.F., Sturfelt, G., Nyhäll-Wåhlin, B.-M., Truedsson, L., Dechant, S.A., and Matteson, E.L. (2005). The impact of HLA-DRB1 genes on extra-articular disease manifestations in rheumatoid arthritis. Arthritis Res. Ther. *7*, R1386–R1393. https://doi.org/10.1186/ar1837.
59. Holoshitz, J. (2010). The rheumatoid arthritis HLA–DRB1 shared epitope. Curr. Opin. Rheumatol. *22*, 293–298. https://doi.org/10.1097/BOR.0b013e328336ba63.
60. Davignon, J.L., Combe, B., and Cantagrel, A. (2021). Cytomegalovirus infection: friend or foe in rheumatoid arthritis? Arthritis Res. Ther. *23*, 16. https://doi.org/10.1186/s13075-020-02398-3.
61. Sherina, N., Hreggvidsdottir, H.S., Bengtsson, C., Hansson, M., Israelsson, L., Alfredsson, L., and Lundberg, K. (2017). Low levels of antibodies against common viruses associate with anti-citrullinated protein antibody-positive rheumatoid arthritis; implications for disease aetiology. Arthritis Res. Ther. *19*, 219. https://doi.org/10.1186/s13075-017-1423-9.
62. Goyette, P., Boucher, G., Mallon, D., Ellinghaus, E., Jostins, L., Huang, H., Ripke, S., Gusareva, E.S., Annese, V., Hauser, S.L., et al. (2015). High-density mapping of the MHC identifies a shared role for HLA-DRB1*01:03 in inflammatory bowel diseases and heterozygous advantage in ulcerative colitis. Nat. Genet. *47*, 172–179. https://doi.org/10.1038/ng.3176.
63. Esposito, S., Bosis, S., Semino, M., and Rigante, D. (2014). Infections and systemic lupus erythematosus. Eur. J. Clin. Microbiol. Infect. Dis. *33*, 1467–1475. https://doi.org/10.1007/s10096-014-2098-7.
64. Nelson, P., Rylance, P., Roden, D., Trela, M., and Tugnet, N. (2014). Viruses as potential pathogenic agents in systemic lupus erythematosus. Lupus *23*, 596–605. https://doi.org/10.1177/0961203314531637.
65. Doaty, S., Agrawal, H., Bauer, E., and Furst, D.E. (2016). Infection and Lupus: Which Causes Which? Curr. Rheumatol. Rep. *18*, 13. https://doi.org/10.1007/s11926-016-0561-4.
66. Illescas-Montes, R., Corona-Castro, C.C., Melguizo-Rodríguez, L., Ruiz, C., and Costela-Ruiz, V.J. (2019). Infectious processes and systemic lupus erythematosus. Immunology *158*, 153–160. https://doi.org/10.1111/imm.13103.
67. Halenius, A., and Hengel, H. (2014). Human cytomegalovirus and autoimmune disease. BioMed. Res. Int. *2014*, 472978. https://doi.org/10.1155/2014/472978.
68. Braendstrup, P., Mortensen, B.K., Justesen, S., Østerby, T., Rasmussen, M., Hansen, A.M., Christiansen, C.B., Hansen, M.B., Nielsen, M., Vindeløv, L., et al. (2014). Identification and

HLA-tetramer-validation of human CD4+and CD8+ T cell responses against HCMV proteins IE1 and IE2. PLoS One *9*, e94892. https://doi.org/10.1371/journal.pone.0094892.

69. Slezak, S.L., Bettinotti, M., Selleri, S., Adams, S., Marincola, F.M., and Stroncek, D.F. (2007). CMV pp65 and IE-1 T cell epitopes recognized by healthy subjects. J. Transl. Med. *5*, 17. https://doi.org/10.1186/1479-5876-5-17.
70. Hasan, A.N., Doubrovina, E., Sottile, R., Prockop, S., Klatt, M.G., Heller, G., Selvakumar, A., Barnett, L., Hsu, K.C., and O'Reilly, R.J. (2022). Dominant epitopes presented by prevalent HLA alleles permit wide use of banked CMVpp65 T cells in adoptive therapy. Blood Adv. *6*, 4859–4872. https://doi.org/10.1182/bloodadvances.2022007005.
71. Diamond, D.J., York, J., Sun, J.Y., Wright, C.L., and Forman, S.J. (1997). Development of a candidate HLA A*0201 restricted peptide-based vaccine against human cytomegalovirus infection. Blood *90*, 1751–1767.
72. Damotte, V., Zhao, C., Lin, C., Williams, E., Louzoun, Y., Madbouly, A., Kotlarz, R., McDaniel, M., Norman, P.J., Wang, Y., et al. (2024). Multiple measures for self-identification improve matching donors with patients in unrelated hematopoietic stem cell transplant. Commun. Med. *4*, 189. https://doi.org/10.1038/s43856-024-00620-w.
73. Hollenbach, J.A., Saperstein, A., Albrecht, M., Vierra-Green, C., Parham, P., Norman, P.J., and Maiers, M. (2015). Race, Ethnicity and Ancestry in Unrelated Transplant Matching for the National Marrow Donor Program: A Comparison of Multiple Forms of Self-Identification with Genetics. PLoS One *10*, e0135960. https://doi.org/10.1371/journal.pone.0135960.
74. Horowitz, A.L., Saperstein, A., Little, J., Maiers, M., and Hollenbach, J.A. (2019). Consumer (dis-)interest in Genetic Ancestry Testing: The roles of race, immigration, and ancestral certainty. New Genet. Soc. *38*, 165–194. https://doi.org/10.1080/14636778.2018.1562327.
75. Johfre, S.S., Saperstein, A., and Hollenbach, J.A. (2021). Measuring Race and Ancestry in the Age of Genetic Testing. Demography *58*, 785–810. https://doi.org/10.1215/00703370-9142013.
76. Bate, S.L., Dollard, S.C., and Cannon, M.J. (2010). Cytomegalovirus seroprevalence in the United States: the national health and nutrition examination surveys, 1988-2004. Clin. Infect. Dis. *50*, 1439–1447. https://doi.org/10.1086/652438.

The American Journal of Human Genetics, Volume *112*

Supplemental information

Human leukocyte antigen variation is associated with cytomegalovirus serostatus in healthy individuals

Juliano A. Boquett, Jürgen Sauter, Alexander H. Schmidt, Martin Maiers, and Jill A. Hollenbach

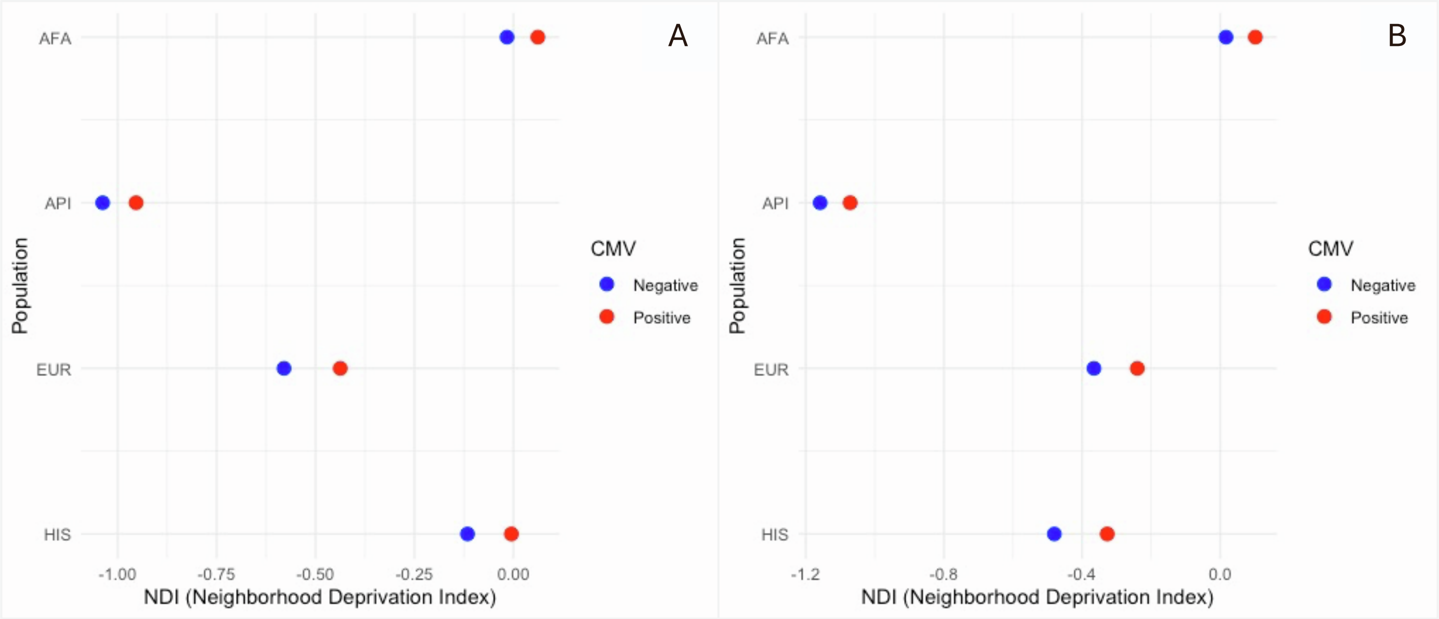

Figure S1. NDI (neighborhood deprivation index) mean among population subgroups. **A)** NMDP cohort. **B)** DKMS cohort. AFA: Black or African American; API: Asian or Pacific Islander; EUR: European descent; HIS: Hispanic or Latino. p-value < 0.05 in each population subgroup in both cohorts (Wilcoxon Rank Sum test).

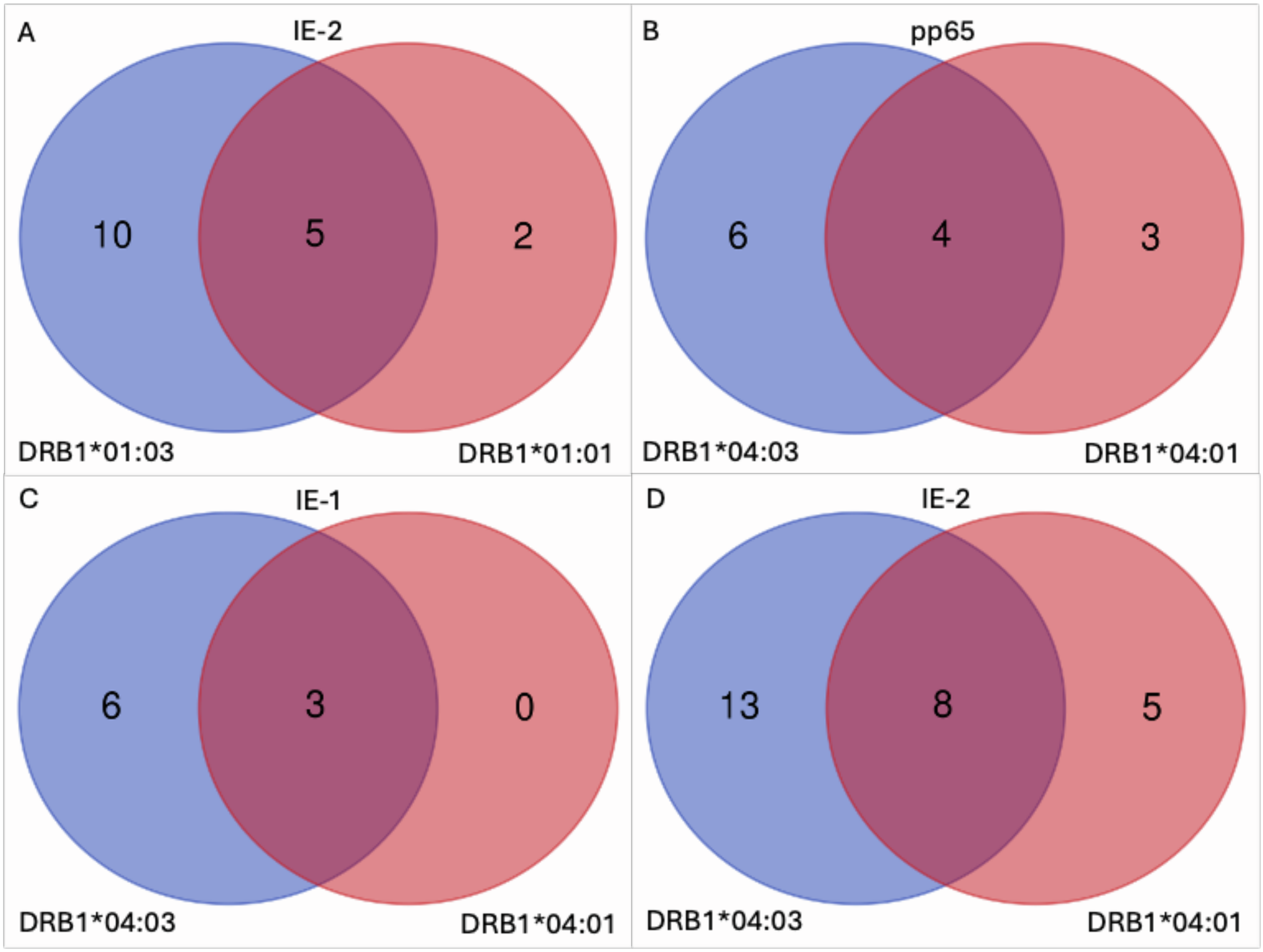

Figure S2. Venn diagrams for stronger binder (SB) peptides shared between *HLA-DRB1* alleles and CMV proteins. **A)** Number of SB for IE-2 protein and *HLA-DRB1*01:03* and *HLA-DRB1*01:01* alleles. **B)** Number of SB for pp65 protein and *HLA-DRB1*04:03* and *HLA-DRB1*04:01* alleles. **C)** Number of SB for IE-1 protein and *HLA-DRB1*04:03* and *HLA-DRB1*04:01* alleles. **D)** Number of SB for IE-2 protein and *HLA-DRB1*04:03* and *HLA-DRB1*04:01* alleles. *HLA-DRB1*01:01* showed no SB for pp65 protein and *HLA-DRB1*01:03* showed no SB for IE-1 protein.

Table S1. NDI quintiles distribution among population subgroups

NDI quintile	NMDP			DKMS		
	**Case** [n (%)]	**Control** [n (%)]	**p-value***	**Case** [n (%)]	**Control** [n (%)]	**p-value***
Black or African American						
1-Least deprivation	998 (17.8)	2208 (19.8)		280 (8.6)	247 (10.4)	
2-Below avg deprivation	1201 (21.4)	2476 (22.2)		479 (14.7)	373 (15.8)	
3-Average deprivation	1067 (19)	2259 (20.3)		581 (17.8)	423 (17.9)	
4-Above avg deprivation	1130 (20.2)	2086 (18.7)		738 (22.6)	535 (22.6)	
5-Most deprivation	1206 (21.5)	2121 (19)	<0.001	1185 (36.3)	787 (33.3)	<0.001
Asian or Pacific Islander						
1-Least deprivation	6919 (54.5)	9110 (57.4)		3246 (44.3)	1658 (47.3)	
2-Below avg deprivation	2929 (23.1)	3590 (22.6)		1724 (23.5)	835 (23.8)	
3-Average deprivation	1587 (12.5)	1767 (11.1)		1133 (15.4)	473 (13.5)	
4-Above avg deprivation	816 (6.4)	955 (6)		741 (10.1)	343 (9.8)	
5-Most deprivation	445 (3.5)	457 (2.9)	<0.001	490 (6.7)	199 (5.7)	<0.001
European descent						
1-Least deprivation	25306 (32.4)	73037 (37.6)		8128 (16.8)	16319 (21)	
2-Below avg deprivation	19881 (25.5)	50919 (26.2)		9349 (19.4)	16628 (21.3)	
3-Average deprivation	15353 (19.7)	35149 (18.1)		9839 (20.4)	16055 (20.6)	
4-Above avg deprivation	11439 (14.7)	24001 (12.3)		10321 (21.4)	15418 (19.8)	
5-Most deprivation	6077 (7.8)	11340 (5.8)	<0.001	10667 (22.1)	13468 (17.3)	<0.001
Hispanic or Latino						
1-Least deprivation	4010 (20.7)	7105 (24.3)		500 (8.8)	524 (13.2)	
2-Below avg deprivation	4073 (21)	6589 (22.5)		759 (13.3)	628 (15.8)	
3-Average deprivation	3677 (19)	5310 (18.1)		987 (17.3)	746 (18.8)	
4-Above avg deprivation	3495 (18)	4647 (15.9)		1314 (23)	832 (21)	
5-Most deprivation	4130 (21.3)	5616 (19.2)	<0.001	2149 (37.6)	1234 (31.1)	<0.001

*Chi-square test.

Table S2. Frequency of significant *HLA* alleles associated with CMV in the NMDP cohort that replicates in the DKMS cohort

	**NMDP - N (Freq%)**		**DKMS - N (Freq %)**	
***HLA* Allele**	**Control**	**Case**	**Control**	**Case**
Black or African American				
*DRB1*15:01*	643 (2.9)	248 (2.2)	162 (3.4)	157 (2.4)
Asian or Pacific Islander				
*A*01:01*	1890 (6)	1662 (6.5)	562 (8)	1425 (9.7)
*A*02:07*	1464 (4.6)	1023 (4)	297 (4.2)	428 (2.9)
*B*40:01*	2473 (7.8)	1768 (7)	511 (7.3)	683 (4.7)
*DRB1*14:04*	834 (2.6)	745 (2.9)	236 (3.4)	687 (4.7)
*DRB1*04:05*	1591 (5)	1132 (4.5)	304 (4.3)	420 (2.9)
*DQB1*04:01*	1243 (3.9)	835 (3.3)	230 (3.3)	317 (2.2)
*DQB1*06:09*	572 (1.8)	368 (1.4)	129 (1.8)	201 (1.4)
European descent				
*B*55:01*	7144 (1.8)	3065 (2)	2892 (1.9)	1960 (2)
*B*15:01*	22465 (5.8)	9706 (6.2)	9047 (5.8)	6017 (6.2)
*C*03:03*	20308 (5.2)	8568 (5.5)	8172 (5.2)	5640 (5.8)
*DRB1*01:03*	5238 (1.3)	1321 (0.8)	2138 (1.4)	903 (0.9)
*DRB1*04:03*	3534 (0.9)	1652 (1.1)	1355 (0.9)	1046 (1.1)
*DRB1*07:01*	53101 (13.7)	20634 (13.2)	21114 (13.6)	12725 (13.2)
*DQB1*06:03*	23942 (6.2)	9228 (5.9)	9571 (6.1)	6176 (6.4)
Hispanic or Latino				
*B*35:17*	763 (1.3)	651 (1.7)	108 (1.4)	214 (1.9)
*C*04:01*	9056 (15.5)	6554 (16.9)	1258 (15.9)	1963 (17.2)
*C*07:01*	6329 (10.8)	3835 (9.9)	879 (11.1)	1053 (9.2)
*DRB1*01:03*	565 (1)	182 (0.5)	66 (0.8)	60 (0.5)
*DRB1*04:03*	950 (1.6)	787 (2)	137 (1.7)	263 (2.3)
*DRB1*07:01*	6662 (11.4)	4057 (10.5)	869 (11)	1090 (9.5)
*DRB1*04:07*	3562 (6.1)	2668 (6.9)	518 (6.5)	893 (7.8)
*DQB1*03:02*	10266 (17.5)	7529 (19.4)	1465 (18.5)	2399 (21)
*DQB1*02:01*	10578 (18.1)	6695 (17.3)	1333 (16.8)	1803 (15.8)

Table S3. Significant associated *HLA* alleles in homozygosis

	NMDP		
***HLA* Allele**	**OR**	**$CI_{97.5\%}$**	**p-value**
*DRB1*01:03/01:03*			
European descent	0.259	0.076 – 0.661	**1.18E-02**
*DRB1*04:03/04:03*			
Asian or Pacific Islander	1.778	1.041 – 3.079	**3.63E-02**
European descent	3.083	1.642 – 5.874	**4.85E-04**
Hispanic or Latino	0.643	0.192 – 1.875	0.44
*DRB1*07:01/07:01*			
Black or African American	0.764	0.528 – 1.086	0.143
Asian or Pacific Islander	0.864	0.687 – 1.082	0.206
European descent	0.903	0.844 – 0.964	**2.58E-03**
Hispanic or Latino	0.818	0.684 – 0.975	**2.67E-02**

Logistic regression; OR: odds ratio; CI: confidence interval. Bold: significant values (α = 5.0E-02). There was not enough homozygous count for *HLA-DRB1*01:03* for population subgroups AFA, API and HIS, and for *HLA-DRB1*04:03* AFA population subgroup.

Table S4. *HLA-DRB1* amino acid association analysis with CMV serostatus

**Black or African American**					**Asian or Pacific Islander**					**European descent**					**Hispanic or Latino**				
Position	Residue	OR	$CI_{(95\%)}$	p-value	Position	Residue	OR	$CI_{(95\%)}$	p-value	Position	Residue	OR	$CI_{(95\%)}$	p-value	Position	Residue	OR	$CI_{(95\%)}$	p-value
Pos.-25	K	0.9	0.86 - 0.94	6.71E-06	Pos.-25	K	0.95	0.92 - 0.99	4.52E-03	Pos.-24	F	1.06	1.05 - 1.08	1.56E-14	Pos.-24	F	1.1	1.07 - 1.14	1.59E-09
Pos.-25	R	1.11	1.06 - 1.17	6.71E-06	Pos.-25	R	1.05	1.01 - 1.08	4.52E-03	Pos.-24	L	0.94	0.93 - 0.96	1.56E-14	Pos.-24	L	0.91	0.88 - 0.94	1.59E-09
Pos.-17	A	1.13	1.07 - 1.2	5.48E-06	Pos.-16	A	0.95	0.92 - 0.99	4.52E-03	Pos.-17	A	1.03	1.02 - 1.05	7.18E-07	Pos.-17	A	1.05	1.02 - 1.09	1.87E-03
Pos.-17	T	0.88	0.83 - 0.93	5.48E-06	Pos.-16	V	1.05	1.01 - 1.08	4.52E-03	Pos.-17	T	0.97	0.95 - 0.98	7.18E-07	Pos.-17	T	0.95	0.92 - 0.98	1.87E-03
Pos.-16	A	0.9	0.86 - 0.94	6.71E-06	Pos.-1	A	0.94	0.9 - 0.98	5.81E-03	Pos.4	Q	0.96	0.95 - 0.98	1.64E-05	Pos.-1	A	1.08	1.03 - 1.13	9.19E-04
Pos.-16	V	1.11	1.06 - 1.17	6.71E-06	Pos.-1	S	1.06	1.02 - 1.11	5.81E-03	Pos.4	R	1.04	1.02 - 1.06	1.64E-05	Pos.-1	S	0.93	0.88 - 0.97	9.19E-04
Pos.-1	A	1.14	1.06 - 1.21	1.37E-04	Pos.4	Q	0.94	0.9 - 0.98	3.19E-03	Pos.13	F	0.97	0.95 - 0.98	2.25E-04	Pos.4	Q	0.93	0.89 - 0.96	1.18E-04
Pos.-1	S	0.88	0.82 - 0.94	1.37E-04	Pos.4	R	1.07	1.02 - 1.12	3.19E-03	Pos.13	H	1.06	1.05 - 1.08	1.67E-14	Pos.4	R	1.08	1.04 - 1.12	1.18E-04
Pos.9	E	1.12	1.07 - 1.18	2.43E-06	Pos.9	E	1.03	1 - 1.07	4.92E-02	Pos.13	R	0.98	0.96 - 1	1.55E-02	Pos.9	E	1.08	1.05 - 1.11	9.72E-08
Pos.9	W	0.88	0.84 - 0.93	5.79E-07	Pos.10	Q	0.95	0.92 - 0.99	4.82E-03	Pos.13	S	1.01	1 - 1.02	4.40E-02	Pos.9	W	0.92	0.9 - 0.95	1.12E-08
Pos.10	Q	0.9	0.86 - 0.94	7.23E-06	Pos.10	Y	1.06	1.02 - 1.09	1.65E-03	Pos.13	Y	0.96	0.95 - 0.98	2.21E-05	Pos.11	G	0.91	0.87 - 0.95	7.48E-06
Pos.10	Y	1.1	1.05 - 1.16	2.36E-05	Pos.11	G	0.92	0.87 - 0.98	6.19E-03	Pos.14	E	1.04	1.02 - 1.06	2.21E-05	Pos.11	P	0.96	0.92 - 1	3.00E-02
Pos.11	P	0.87	0.82 - 0.93	2.49E-05	Pos.11	L	0.87	0.78 - 0.98	1.58E-02	Pos.14	K	0.96	0.95 - 0.98	2.21E-05	Pos.11	V	1.1	1.06 - 1.13	3.04E-09
Pos.11	S	1.1	1.05 - 1.16	2.36E-05	Pos.11	S	1.06	1.02 - 1.09	1.65E-03	Pos.25	Q	0.96	0.95 - 0.98	2.20E-05	Pos.14	E	1.1	1.05 - 1.15	7.48E-06
Pos.12	K	0.91	0.86 - 0.95	2.36E-05	Pos.12	K	0.95	0.92 - 0.98	1.65E-03	Pos.25	R	1.04	1.02 - 1.06	2.20E-05	Pos.14	K	0.91	0.87 - 0.95	7.48E-06
Pos.12	T	1.1	1.05 - 1.16	2.36E-05	Pos.12	T	1.06	1.02 - 1.09	1.65E-03	Pos.26	L	0.97	0.95 - 0.99	4.71E-04	Pos.25	Q	0.91	0.87 - 0.95	7.48E-06
Pos.13	R	0.87	0.82 - 0.93	2.49E-05	Pos.13	F	0.94	0.9 - 0.99	1.63E-02	Pos.26	Y	1.02	1 - 1.04	2.87E-02	Pos.25	R	1.1	1.05 - 1.15	7.48E-06
Pos.13	S	1.09	1.04 - 1.14	3.55E-04	Pos.13	S	1.06	1.02 - 1.1	4.33E-03	Pos.28	D	1.04	1.03 - 1.05	4.79E-09	Pos.26	L	0.96	0.92 - 1	4.89E-02
Pos.26	Y	1.15	1.06 - 1.24	3.67E-04	Pos.13	Y	0.92	0.87 - 0.98	6.19E-03	Pos.28	E	0.96	0.95 - 0.97	6.96E-09	Pos.28	D	1.04	1.01 - 1.07	3.86E-03
Pos.30	H	0.93	0.87 - 0.99	1.53E-02	Pos.14	E	1.08	1.02 - 1.15	6.19E-03	Pos.30	C	0.96	0.94 - 0.98	2.84E-05	Pos.28	E	0.95	0.93 - 0.98	1.09E-03
Pos.30	Y	1.08	1.03 - 1.13	2.25E-03	Pos.14	K	0.92	0.87 - 0.98	6.19E-03	Pos.30	L	0.96	0.95 - 0.98	2.21E-05	Pos.30	L	0.91	0.87 - 0.95	7.48E-06
Pos.32	H	1.07	1.02 - 1.12	5.27E-03	Pos.25	Q	0.92	0.87 - 0.98	6.19E-03	Pos.30	Y	1.04	1.03 - 1.05	5.20E-09	Pos.30	Y	1.06	1.03 - 1.09	8.02E-05
Pos.32	Y	0.93	0.89 - 0.98	5.27E-03	Pos.25	R	1.08	1.02 - 1.15	6.19E-03	Pos.31	F	1.03	1.02 - 1.05	2.25E-04	Pos.33	H	1.1	1.07 - 1.14	1.79E-09
Pos.37	N	1.08	1.03 - 1.14	2.34E-03	Pos.30	C	0.87	0.78 - 0.98	1.58E-02	Pos.31	I	0.96	0.94 - 0.98	2.63E-05	Pos.33	N	0.91	0.88 - 0.94	1.79E-09
Pos.37	S	0.88	0.84 - 0.93	6.56E-06	Pos.30	H	1.06	1.01 - 1.12	2.45E-02	Pos.32	H	1.01	1 - 1.03	4.40E-02	Pos.37	F	0.9	0.86 - 0.93	4.21E-08
Pos.37	Y	1.06	1.01 - 1.11	2.27E-02	Pos.30	L	0.92	0.87 - 0.98	6.19E-03	Pos.32	Y	0.99	0.97 - 1	4.40E-02	Pos.37	S	0.95	0.92 - 0.98	9.10E-04
Pos.67	I	0.93	0.89 - 0.97	1.94E-03	Pos.31	F	1.06	1.01 - 1.11	1.63E-02	Pos.33	H	1.06	1.05 - 1.08	1.67E-14	Pos.37	Y	1.09	1.07 - 1.12	9.60E-12
Pos.67	L	1.06	1.01 - 1.12	1.27E-02	Pos.31	I	0.94	0.89 - 0.99	2.50E-02	Pos.33	N	0.94	0.93 - 0.96	1.67E-14	Pos.47	F	0.94	0.92 - 0.97	3.48E-05
Pos.71	A	0.88	0.82 - 0.94	1.41E-04	Pos.32	H	1.04	1.01 - 1.08	2.04E-02	Pos.37	F	0.97	0.96 - 0.99	6.58E-04	Pos.47	Y	1.06	1.03 - 1.09	3.48E-05
Pos.71	K	1.12	1.06 - 1.19	7.60E-05	Pos.32	Y	0.96	0.93 - 0.99	2.04E-02	Pos.37	S	0.97	0.95 - 0.98	7.76E-07	Pos.57	A	0.87	0.8 - 0.95	2.62E-03
Pos.73	A	0.95	0.9 - 1	4.16E-02	Pos.37	L	1.06	1.01 - 1.12	2.28E-02	Pos.37	Y	1.04	1.03 - 1.05	3.46E-10	Pos.57	D	1.04	1.01 - 1.07	1.76E-02
Pos.73	G	1.06	1 - 1.12	4.16E-02	Pos.47	F	1.1	1.07 - 1.14	8.61E-09	Pos.57	D	1.03	1.01 - 1.04	2.84E-04	Pos.57	S	1.08	1.03 - 1.13	2.68E-03
Pos.74	A	0.93	0.89 - 0.97	2.40E-03	Pos.47	Y	0.91	0.88 - 0.94	8.61E-09	Pos.57	V	0.97	0.95 - 0.98	3.48E-05	Pos.57	V	0.93	0.89 - 0.96	1.06E-04
Pos.74	R	1.14	1.07 - 1.22	1.06E-04	Pos.50	A	1.33	1.04 - 1.7	1.89E-02	Pos.71	E	0.94	0.93 - 0.96	1.32E-11	Pos.60	H	0.87	0.8 - 0.96	2.86E-03
Pos.77	N	1.14	1.07 - 1.22	1.06E-04	Pos.50	V	0.75	0.59 - 0.96	1.89E-02	Pos.71	K	1.06	1.05 - 1.08	<2.22E-16	Pos.60	S	0.93	0.89 - 0.96	7.21E-05
Pos.77	T	0.88	0.82 - 0.94	1.06E-04	Pos.58	A	0.86	0.8 - 0.92	1.13E-05	Pos.85	A	0.96	0.92 - 0.99	9.80E-03	Pos.60	Y	1.09	1.06 - 1.13	8.33E-07
Pos.96	H	1.1	1.05 - 1.16	1.44E-04	Pos.58	E	1.16	1.09 - 1.24	1.13E-05	Pos.85	V	1.05	1.01 - 1.08	9.80E-03	Pos.67	I	0.9	0.87 - 0.92	8.02E-15
Pos.96	Q	0.89	0.84 - 0.95	1.90E-04	Pos.60	H	1.08	1 - 1.16	4.24E-02	Pos.96	E	0.96	0.94 - 0.98	2.96E-05	Pos.67	L	1.08	1.06 - 1.11	7.01E-10
Pos.133	L	0.87	0.82 - 0.93	2.43E-05	Pos.67	F	1.07	1.03 - 1.11	6.86E-04	Pos.96	Q	0.98	0.97 - 1	4.36E-02	Pos.70	D	0.95	0.93 - 0.98	1.91E-04
Pos.133	R	1.14	1.07 - 1.22	2.43E-05	Pos.71	A	1.06	1.02 - 1.1	6.48E-03	Pos.96	Y	1.06	1.05 - 1.08	1.81E-14	Pos.70	Q	1.06	1.03 - 1.09	1.97E-05
Pos.140	A	0.89	0.85 - 0.94	2.31E-06	Pos.71	R	0.96	0.93 - 1	2.55E-02	Pos.98	E	1.02	1.01 - 1.03	4.36E-03	Pos.71	A	0.92	0.88 - 0.97	7.34E-04

Pos.140	T	1.12	1.07 - 1.18	2.31E-06	Pos.74	L	0.89	0.82 - 0.96	1.31E-03	Pos.98	K	0.98	0.97 - 0.99	4.36E-03	Pos.71	E	0.92	0.89 - 0.96	6.08E-05
Pos.142	M	0.87	0.82 - 0.93	2.43E-05	Pos.74	Q	0.92	0.87 - 0.98	6.19E-03	Pos.104	A	1.02	1.01 - 1.03	4.36E-03	Pos.71	R	1.08	1.05 - 1.11	9.14E-09
Pos.142	V	1.14	1.07 - 1.22	2.43E-05	Pos.78	V	0.94	0.9 - 0.98	3.12E-03	Pos.104	S	0.98	0.97 - 0.99	4.36E-03	Pos.73	A	1.06	1.03 - 1.1	2.72E-04
Pos.149	H	1.1	1.05 - 1.16	2.23E-05	Pos.78	Y	1.07	1.02 - 1.12	3.12E-03	Pos.120	N	1.06	1.05 - 1.08	4.68E-15	Pos.73	G	0.94	0.91 - 0.97	2.72E-04
Pos.149	Q	0.91	0.86 - 0.95	2.23E-05	Pos.85	A	1.05	1 - 1.11	4.87E-02	Pos.120	S	0.94	0.93 - 0.95	4.68E-15	Pos.74	A	0.97	0.94 - 0.99	7.44E-03
Pos.233	R	1.08	1.03 - 1.13	7.70E-04	Pos.85	V	0.95	0.9 - 1	4.87E-02	Pos.133	L	0.98	0.96 - 1	1.56E-02	Pos.74	E	1.11	1.07 - 1.16	1.38E-08
Pos.233	T	0.92	0.88 - 0.97	7.70E-04	Pos.86	G	0.94	0.91 - 0.98	6.88E-04	Pos.133	R	1.02	1 - 1.04	1.56E-02	Pos.74	L	1.07	1.02 - 1.11	4.71E-03
					Pos.86	V	1.06	1.02 - 1.1	6.88E-04	Pos.140	A	0.96	0.94 - 0.97	5.46E-14	Pos.74	Q	0.91	0.87 - 0.95	7.20E-06
					Pos.96	E	0.87	0.78 - 0.98	1.60E-02	Pos.140	T	1.05	1.03 - 1.06	5.46E-14	Pos.78	V	0.93	0.89 - 0.96	1.32E-04
					Pos.98	E	0.94	0.91 - 0.97	5.92E-04	Pos.142	M	0.98	0.96 - 1	1.56E-02	Pos.78	Y	1.08	1.04 - 1.12	1.32E-04
					Pos.98	K	1.07	1.03 - 1.1	5.92E-04	Pos.142	V	1.02	1 - 1.04	1.56E-02	Pos.96	H	0.97	0.94 - 0.99	1.28E-02
					Pos.104	A	0.94	0.91 - 0.97	5.92E-04	Pos.180	L	1.06	1.05 - 1.08	1.81E-14	Pos.96	Q	0.96	0.93 - 1	4.71E-02
					Pos.104	S	1.07	1.03 - 1.1	5.92E-04	Pos.180	V	0.94	0.93 - 0.96	1.81E-14	Pos.96	Y	1.1	1.07 - 1.14	1.60E-09
					Pos.149	H	1.06	1.02 - 1.09	1.62E-03	Pos.181	M	0.97	0.95 - 0.98	1.22E-04	Pos.98	E	1.04	1.01 - 1.06	1.08E-02
					Pos.149	Q	0.95	0.92 - 0.98	1.62E-03	Pos.181	T	1.03	1.02 - 1.05	1.22E-04	Pos.98	K	0.97	0.94 - 0.99	1.08E-02
					Pos.181	M	0.94	0.9 - 0.98	2.03E-03						Pos.104	A	1.04	1.01 - 1.06	1.08E-02
					Pos.181	T	1.07	1.02 - 1.11	2.03E-03						Pos.104	S	0.97	0.94 - 0.99	1.08E-02
					Pos.189	R	1.13	1.05 - 1.22	1.01E-03						Pos.120	N	1.1	1.06 - 1.13	2.63E-09
					Pos.189	S	0.88	0.82 - 0.95	1.01E-03						Pos.120	S	0.91	0.88 - 0.94	2.63E-09
					Pos.233	R	1.09	1.05 - 1.12	2.08E-06						Pos.133	L	0.96	0.92 - 1	3.02E-02
					Pos.233	T	0.92	0.89 - 0.95	2.08E-06						Pos.133	R	1.05	1 - 1.09	3.02E-02
															Pos.140	A	0.93	0.9 - 0.95	8.26E-08
															Pos.140	T	1.08	1.05 - 1.11	8.26E-08
															Pos.142	M	0.96	0.92 - 1	3.02E-02
															Pos.142	V	1.05	1 - 1.09	3.02E-02
															Pos.180	L	1.1	1.07 - 1.14	1.60E-09
															Pos.180	V	0.91	0.88 - 0.94	1.60E-09
															Pos.181	M	0.93	0.9 - 0.97	3.17E-04
															Pos.181	T	1.07	1.03 - 1.11	3.17E-04
															Pos.189	R	0.94	0.9 - 0.98	4.32E-03
															Pos.189	S	1.07	1.02 - 1.12	4.32E-03

Highlighted in red: residues that lies in the binding pocket P4 (residues 71 and 74) and P7 (residues 67 and 71).

Table S5. Peptide binding prediction among the most immunogenic CMV proteins and *HLA* class II associated with CMV in this study.

CMV protein	Peptide	*HLA* Allele	Score_EL	%Rank_EL	Bind Level	*HLA* Allele	Score_EL	%Rank_EL	Bind Level
pp65	DTPVLPHETRLLQTG	*DRB1*01:03*	0.545284	1.41	1 SB	*DRB1*01:01*	0.07773	12.44	52
pp65	ASVLGPISGHVLKAV	*DRB1*01:03*	0.528303	1.51	2 SB	*DRB1*01:01*	0.488896	2.78	3 WB
pp65	PKNMIIKPGKISHIM	*DRB1*01:03*	0.504027	1.68	3 SB	*DRB1*01:01*	0.039001	18.18	81
pp65	CPKNMIIKPGKISHI	*DRB1*01:03*	0.481895	1.87	4 SB	*DRB1*01:01*	0.033076	19.79	94
pp65	RGRLKAESTVAPEED	*DRB1*04:01*	0.828983	0.43	1 SB	*DRB1*04:03*	0.743531	0.77	3 SB
pp65	VSQYTPDSTPCHRGD	*DRB1*04:01*	0.820568	0.46	2 SB	*DRB1*04:03*	0.231153	9.44	52 WB
pp65	TRGRLKAESTVAPEE	*DRB1*04:01*	0.750475	0.74	3 SB	*DRB1*04:03*	0.663426	1.38	6 SB
pp65	LVSQYTPDSTPCHRG	*DRB1*04:01*	0.72639	0.87	4 SB	*DRB1*04:03*	0.15702	13.47	73
pp65	GGAMAGASTSAGRKR	*DRB1*04:01*	0.671587	1.19	5 SB	*DRB1*04:03*	0.836002	0.3	1 SB
pp65	SQYTPDSTPCHRGDN	*DRB1*04:01*	0.574326	1.89	6 SB	*DRB1*04:03*	0.085767	20.69	108
pp65	GGGAMAGASTSAGRK	*DRB1*04:01*	0.56122	1.99	7 SB	*DRB1*04:03*	0.78398	0.55	2 SB
IE1	LSSVIVAENSDQEES	*DRB1*04:01*	0.757261	0.71	1 SB	*DRB1*04:03*	0.873976	0.16	1 SB
IE1	SSVIVAENSDQEESE	*DRB1*04:01*	0.743833	0.78	2 SB	*DRB1*04:03*	0.845421	0.26	2 SB
IE1	PLSSVIVAENSDQEE	*DRB1*04:01*	0.608957	1.6	3 SB	*DRB1*04:03*	0.816709	0.39	3 SB
IE2	GIQIIYTRNHEVKSE	*DRB1*01:03*	0.859046	0.17	1 SB	*DRB1*01:01*	0.685628	1.47	5 SB
IE2	STPFLMEHTMPVTHP	*DRB1*01:03*	0.853592	0.19	2 SB	*DRB1*01:01*	0.95804	0.13	1 SB
IE2	KGIQIIYTRNHEVKS	*DRB1*01:03*	0.801849	0.32	3 SB	*DRB1*01:01*	0.561059	2.22	8 WB
IE2	LSTPFLMEHTMPVTH	*DRB1*01:03*	0.755569	0.46	4 SB	*DRB1*01:01*	0.897276	0.41	2 SB
IE2	IQIIYTRNHEVKSEV	*DRB1*01:03*	0.691367	0.69	5 SB	*DRB1*01:01*	0.357264	4.06	17 WB
IE2	DYRNMIIHAATPVDL	*DRB1*01:03*	0.683552	0.72	6 SB	*DRB1*01:01*	0.263396	5.36	28 WB
IE2	SDYRNMIIHAATPVD	*DRB1*01:03*	0.666465	0.79	7 SB	*DRB1*01:01*	0.304748	4.72	21WB
IE2	YRNMIIHAATPVDLL	*DRB1*01:03*	0.658343	0.82	8 SB	*DRB1*01:01*	0.272926	5.2	25 WB
IE2	TPFLMEHTMPVTHPP	*DRB1*01:03*	0.654846	0.83	9 SB	*DRB1*01:01*	0.798646	0.88	3 SB
IE2	NKGIQIIYTRNHEVK	*DRB1*01:03*	0.650678	0.85	10 SB	*DRB1*01:01*	0.333038	4.35	18 WB
IE2	SSDYRNMIIHAATPV	*DRB1*01:03*	0.617826	0.99	11 SB	*DRB1*01:01*	0.534337	2.41	10 WB
IE2	RSSDYRNMIIHAATP	*DRB1*01:03*	0.554302	1.35	12 SB	*DRB1*01:01*	0.507004	2.63	11 WB
IE2	ALSTPFLMEHTMPVT	*DRB1*01:03*	0.53811	1.45	13 SB	*DRB1*01:01*	0.715405	1.3	4 SB
IE2	RNMIIHAATPVDLLG	*DRB1*01:03*	0.494868	1.76	14 SB	*DRB1*01:01*	0.3818	3.78	14 WB
IE2	TRPFKVIIKPPVPPA	*DRB1*01:03*	0.492674	1.78	15 SB	*DRB1*01:01*	0.283	5.03	23 WB
IE2	LGDILAQAVNHAGID	*DRB1*04:01*	0.847132	0.36	1 SB	*DRB1*04:03*	0.882893	0.13	1 SB
IE2	STPFLMEHTMPVTHP	*DRB1*04:01*	0.84588	0.36	2 SB	*DRB1*04:03*	0.523165	2.76	26 WB
IE2	GDILAQAVNHAGIDS	*DRB1*04:01*	0.776221	0.63	3 SB	*DRB1*04:03*	0.840081	0.28	6 SB
IE2	ELGDILAQAVNHAGI	*DRB1*04:01*	0.755582	0.72	4 SB	*DRB1*04:03*	0.813539	0.41	7 SB
IE2	LSTPFLMEHTMPVTH	*DRB1*04:01*	0.740686	0.79	5 SB	*DRB1*04:03*	0.406763	4.5	45 WB
IE2	SSDYRNMIIHAATPV	*DRB1*04:01*	0.715173	0.93	6 SB	*DRB1*04:03*	0.467666	3.52	32 WB
IE2	RSSDYRNMIIHAATP	*DRB1*04:01*	0.709733	0.97	7 SB	*DRB1*04:03*	0.466951	3.53	33 WB
IE2	KQVMVRIFSTNQGGF	*DRB1*04:01*	0.68533	1.11	8 SB	*DRB1*04:03*	0.875435	0.16	2 SB
IE2	QVMVRIFSTNQGGFM	*DRB1*04:01*	0.655529	1.28	9 SB	*DRB1*04:03*	0.85674	0.22	3 SB
IE2	AELGDILAQAVNHAG	*DRB1*04:01*	0.65254	1.3	10 SB	*DRB1*04:03*	0.72912	0.85	13 SB
IE2	NKGIQIIYTRNHEVK	*DRB1*04:01*	0.613346	1.57	11 SB	*DRB1*04:03*	0.797372	0.49	8 SB
IE2	PKQVMVRIFSTNQGG	*DRB1*04:01*	0.57969	1.85	12 SB	*DRB1*04:03*	0.849283	0.25	4 SB
IE2	TPFLMEHTMPVTHPP	*DRB1*04:01*	0.561179	1.99	13 SB	*DRB1*04:03*	0.263329	8.17	61 WB

EL: Eluted Ligand mass spectrometry; SB: strong binder; WB: weak binder; Threshold for strong binder (% Rank): 2; Threshold for weak binder (% Rank): 10

Table S6. Charge and volume of the polymorphic residues in the HVR3 for *HLA-DRB1*01:01*, *HLA-DRB1*01:03*, *HLA-DRB1*04:01* and *HLA-DRB1*04:03*

	*HLA-DRB1*01:01*	*HLA-DRB1*01:03*	*HLA-DRB1*04:01*	*HLA-DRB1*04:03*
**Residue 67**				
Amino acid	L - leucine	I - isoleucine	L - leucine	L - leucine
Charge	uncharged	uncharged	uncharged	uncharged
Volume Å3	large 166.7	large 166.7	large 166.7	large 166.7
**Residue 70**				
Amino acid	Q - glutamine	D - aspartic acid	Q - glutamine	Q - glutamine
Charge	uncharged	negative	uncharged	uncharged
Volume Å3	medium 143.8	small 111.1	medium 143.8	medium 143.8
**Residue 71**				
Amino acid	R - arginine	E - glutamic acid	K - lysine	R - arginine
Charge	positive	negative	positive	positive
Volume Å3	large 173.4	medium 138.4	large 168.6	large 173.4
**Residue 74**				
Amino acid	A - alanine	A - alanine	A - alanine	E - glutamic acid
Charge	uncharged	uncharged	uncharged	negative
Volume Å3	very small 88.6	very small 88.6	very small 88.6	medium 138.4
**Residue 86**				
Amino acid	G - glycine	G - glycine	G - glycine	V - valine
Charge	uncharged	uncharged	uncharged	uncharged
Volume Å3	very small 60.1	very small 60.1	very small 60.1	medium 140

Blue: denotes a change in the amino acid residue;
Red: denotes changes in charge and volume.
